# Indium Tribromide‐Catalysed Transfer‐Hydrogenation: Expanding the Scope of the Hydrogenation and of the Regiodivergent DH or HD Addition to Alkenes

**DOI:** 10.1002/chem.202101259

**Published:** 2021-06-21

**Authors:** Luomo Li, Gerhard Hilt

**Affiliations:** ^1^ Institut für Chemie Oldenburg University Carl-von-Ossietzky-Str. 9–11 26111 Oldenburg Germany

**Keywords:** alkenes, catalysis, indium tribromide, regioselectivity, transfer-hydrogenation

## Abstract

The transfer‐hydrogenation as well as the regioselective and regiodivergent addition of H−D from regiospecific deuterated dihydroaromatic compounds to a variety of 1,1‐di‐ and trisubstituted alkenes was realised with InBr_3_ in dichloro(m)ethane. In comparison with the previously reported BF_3_⋅Et_2_O‐catalysed process, electron‐deficient aryl‐substituents can be applied reliably and thereby several restrictions could be lifted, and new types of substrates could be transformed successfully in hydrodeuterogenation as well as deuterohydrogenation transfer‐hydrogenation reactions.

## Graphical abstract

**More is better**: The InBr_3_‐catalysed transfer‐hydrogenation from dihydroaromatic compounds to di‐ and trisubstituted alkenes was realised utilising cyclohexa‐1,4‐diene as H_2_ surrogate in chlorinated solvents, such as DCM and DCE. The scope of the reactions could be expanded towards electron‐deficient aryl‐substituted alkenes and even to a dialkyl‐substituted alkene. The regiodivergent hydrodeuterogenation as well as the deuterohydrogenation were realised with two regiospecifically deuterium‐labelled HD surrogates to access site selective deuterium incorporation in the products.

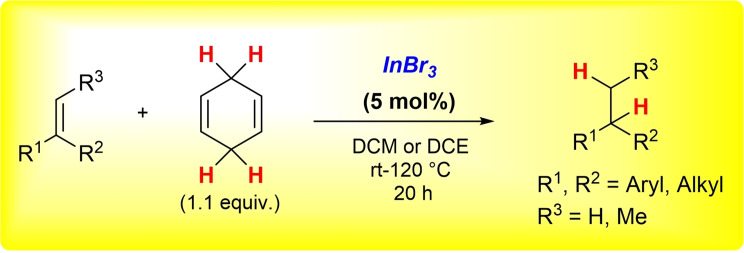

## Introduction

Dihydroaromatic compounds are relatively sensitive towards air and other mild oxidising agents and can be converted easily into the corresponding arenes.[^1^, ^2^, ^3^, ^4^, ^5^, ^6^, ^7^, ^8^, ^9^] Based on these characteristics, dihydroaromatic compounds have found various applications[^10^, ^11^, ^12^, ^13^, ^14^, ^15^] and the use as a surrogate for molecular hydrogen is one of the most prominent examples.[^16^, ^17^, ^18^, ^19^, ^20^, ^21^, ^22^, ^23^] Other applications of substituted 1,4‐cyclohexadienes are the transfer of HBr,^[24]^ HCN[^25^, ^26^] and many other hydrofunctionalisation reactions to transfer various groups[^27^, ^28^, ^29^, ^30^, ^31^, ^32^, ^33^, ^34^, ^35^] to unsaturated starting materials, which have been summarised recently.[^36^, ^37^, ^38^, ^39^]

**Scheme 1:**
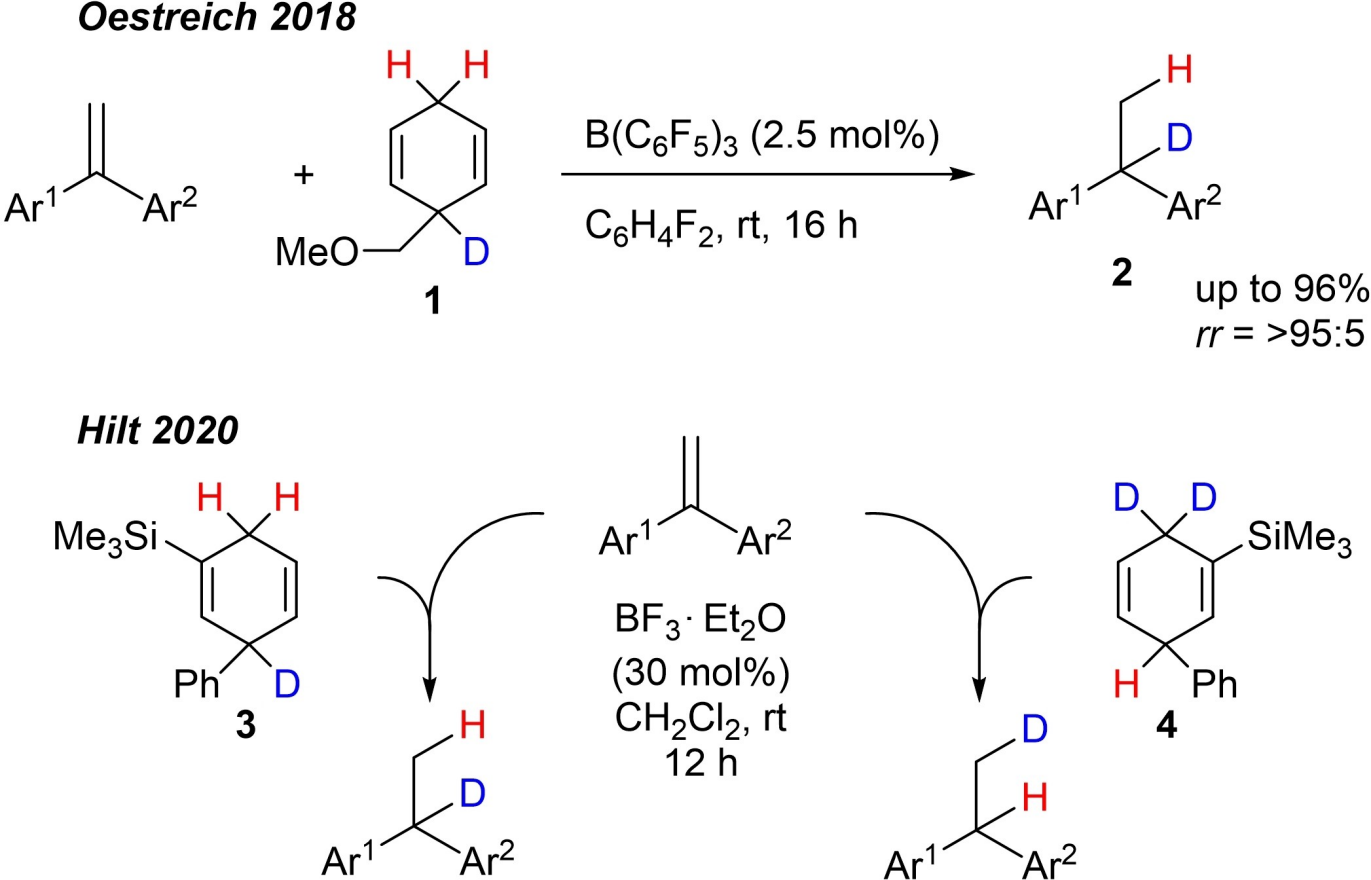
Previous work concerning regioselective hydrodeutero‐genation/deuterohydrogenation reactions.

The Oestreich group pioneered in the regioselective hydrodeuterogenation of 1,1‐diarylalkenes in 2018 by using an unsymmetrical surrogate where the hydrogen and the deuterium atoms are bound differently, so that the hydrogen acts as a hydride equivalent (H^+^) and the deuterium atom as a “heavy” proton (D^−^).^[40]^ The reactions were catalysed by B(C_6_F_5_)_3_ and the desired reduced products were obtained in good to excellent yields and regioisomeric ratios (*rr*=>95 : 5) with the deuterium attached to the higher substituted carbon atom (Scheme 1). In 2020 our group reported the regiodiverse hydrodeuterogenation and the deuterohydrogenation utilising two regioselectively substituted dihydroaromatic compounds **3** and **4** (Scheme 1) which originated from the cobalt‐catalysed Diels‐Alder reaction of selectively deuterated 1,3‐dienes with trimethylsilyl acetylene.^[41]^ For the reduction of 1,1‐disubstituted diaryl alkenes, the catalyst was altered from B(C_6_F_5_)_3_ to the less air‐ and moisture sensitive BF_3_ ⋅ OEt_2_ complex, which had to be applied in a higher catalyst loading of 30 mol%. Nevertheless, good yields and a high incorporation of deuterium at the desired positions was observed. However, some functional groups, such as 1,2‐dimethoxyaryl or electron‐deficient aryl substituents, were not compatible with the reaction conditions and prompted us to search for alternative catalysts with a higher reactivity.

The regioselective labelling of organic molecules is useful in organic, organometallic and biochemistry, because labelled compounds can be used to elucidate reaction mechanisms and biosynthetic pathways.^[42]^ Kinetic isotope effects can be measured when a deuterium‐carbon bond cleavage is incorporated in the rate determining step of a reaction.^[43]^ Also, ^2^H NMR can be used as probe for the determination of Lewis acidities.^[44]^

## Results and Discussion

**Scheme 2:**
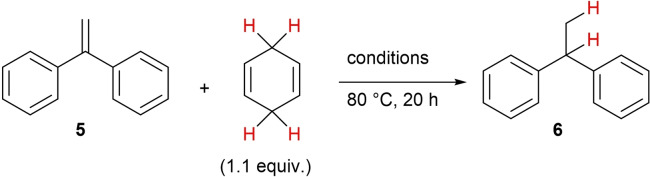
Hydrogenation of alkene **5** utilising CHD as reducing agent and indium‐based catalysts.

In light of these preliminary works, we decided to investigate the transfer‐hydrogenation from dihydroaromatic compounds to alkenes, also with indium‐based catalysts in order to identify substrates which will be accepted by those catalysts to enlarge the scope of the reaction. The optimization for an indium‐salt catalysed process started with the readily available 1,1‐diphenylethene **5** and 1,4‐cyclohexadiene (=CHD) as commercially and readily available starting materials under modified conditions (Scheme 2).

First, we investigated the influence of different solvents in the transfer‐hydrogenation. Donor‐based solvents, such as THF and acetonitrile revealed to be less favourable (Table 1, entries 1 and 2), while surprisingly the non‐polar solvent *n*‐heptane (entry 3) and the weakly coordinating solvent 1,2‐dichloroethane (DCE, entry 4) proved to be very suitable for the reaction and gave the desired product **6** in excellent to quantitative yields. The catalyst loading of 5 mol% seems to be efficient while lower as well as higher catalyst loadings resulted in lower yields (entries 5/6) for no obvious reason. As a commercially available alternative to InBr_3_, also indium triflate was tested, which resulted in a lower yield of **6** (75 %).

**Table 1:** Results of the InBr_3_/In(OTf)_3_‐catalysed hydrogenation of **5** with CHD.^[a]^

no.	Catalyst^[a]^	solvent	yield **6**
1	InBr_3_ (5 mol%)	THF	trace
2	InBr_3_ (5 mol%)	acetonitrile	8 %
3	InBr_3_ (5 mol%)	*n*‐heptane	92 %
4	InBr_3_ (5 mol%)	(CH_2_Cl)_2_	99 % (97 %)^[b]^
5	InBr_3_ (3 mol%)	(CH_2_Cl)_2_	88 %
6	InBr_3_ (10 mol%)	(CH_2_Cl)_2_	84 %
7	In(OTf)_3_ (5 mol%)	(CH_2_Cl)_2_	75 %

[a] The yields were determined by GC‐FID analysis of the crude mixture with mesitylene as internal standard, added after the reaction from a stock solution. [b] Isolated yield.

**Scheme 3:**
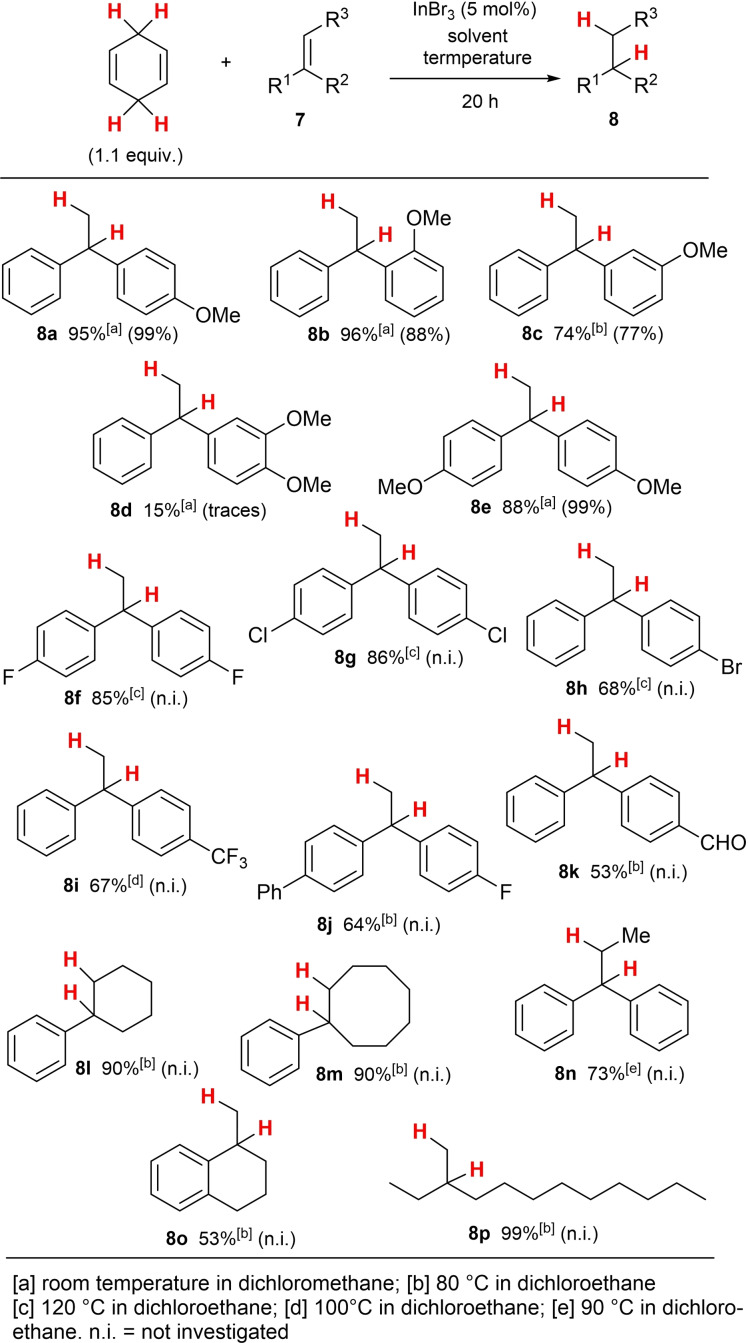
InBr_3_‐catalysed transfer‐hydrogenation to alkenes.

The hitherto best results were obtained, when 5 mol% InBr_3_ was used as a Lewis acid catalyst in DCE as solvent at elevated temperatures (80 °C) for 12 h reaction time, which resulted in an almost quantitative yield of **6** (by GC analysis) and an isolated yield of 97 %. With these reaction conditions in hand, we started the investigation of the InBr_3_‐catalysed transfer‐hydrogenation reaction from CHD to suitable alkenes with a strong focus upon the previously reported results of the corresponding BF_3_ ⋅ OEt_2_‐catalysed hydrogenation reaction to identify similarities or improvements. In this respect, we first applied well‐accepted substrates in the BF_3_ ⋅ OEt_2_‐catalysed reaction, such as several methoxy‐substituted 1,1‐diarylethenes, in the indium tribromide‐catalysed reaction. Later, also substrates with electron‐deficient substituents and tri‐substituted alkenes were tested, which were not applied in the BF_3_ ⋅ OEt_2_‐catalysed transfer‐hydrogenation reaction because of the lower reactivity of the boron‐based catalyst system. The results of these reactions and of the ongoing substrate screening process are summarised in Scheme 3. For direct comparison of the indium‐ and the boron‐catalysed reactions, the yields in brackets refer to the previously reported yields when BF_3_ ⋅ OEt_2_ was applied.[^40^, ^41^]

As can be seen from Scheme 3, the first results of the InBr_3_‐catalysed process are as efficient as the BF_3_ ⋅ OEt_2_‐catalysed reaction for the electron‐rich substrates (**8 a**–**8 c**). Therefore, we continued to direct our attention towards those substrates which proved to be unreactive under the BF_3_ ⋅ OEt_2_‐catalysed conditions and skipped most of the previously positive reported substrates. Early on in this investigation, the previously unreactive substrate **8 d** led to a low yield of 15 %, which could be attributed to the stronger interaction of the 1,2‐dimethoxy subunit with BF_3_ compared to the much more inert InBr_3_ for ligand exchange and inactivation of the catalyst. On the other hand, a slightly lower yield for **8 e** was obtained for the InBr_3_‐ compared to the BF_3_ ⋅ OEt_2_‐catalysed reaction. Even more interesting results were obtained, when halide‐substituted electron‐deficient 1,1‐diaryl‐substituted diaryl ethenes were tested. These types of substrates were very unreactive in the BF_3_ ⋅ OEt_2_‐catalysed reactions (yields <1%) and consequently not investigated in more detail in the previous report.^[41]^

The 4,4'‐difluoro‐derivative **8 f** and the 4,4'‐dichloro‐derivative **8 g** were obtained in good yields of >85 %, however, elevated temperatures had to be applied (120 °C in DCE). The mono‐bromide‐substituted staring material **7 h**, which was unreactive under the boron‐catalysed conditions still shows a good reactivity and resulted in the formation of the product **8 h** in 68 % yield. Also, the application of the 4‐trifluoromethyl‐substituted product **8 i** and the 4‐fluoro‐substituted derivative **8 j** expanded the scope of the reaction and led to the product in 67 % yield.

For the functional group compatibility, we also applied an aldehyde functionalised starting material **7 k** to determine the compatibility of redox‐active functional groups under the reaction conditions. Although the desired aldehyde **8 k** was only isolated in 58 % yield, the proof‐of‐principle was successful, in that respect that the ionic mechanism outlined below is more receptible towards the more stabilised cation intermediate formed upon protonation of the diaryl‐substituted double bound with respect to the aldehyde functionality.

Then we turned our attention towards trisubstituted alkenes and fortunately the indium‐catalysed transfer‐hydrogenation proved to be superior to the boron‐based transformation. Indeed, the products **8 l** and **8 m** could be obtained in 90 %, whereas product **8 n** was isolated in good 73 % yield.

In the BF_3_ ⋅ OEt_2_‐catalysed transfer‐hydrogenations, an aryl substituent proved to be essential for the overall transfer‐hydrogenation process because an efficient mesomeric carbenium ion stabilisation by the arene is needed and an exocyclic carbon‐carbon double bond was not applicable. When InBr_3_ was used as catalyst, the formation of **8 o** could be realised with moderate success. Finally, we also tested a 1,1‐dialky‐substituted alkene, namely 3‐methylene‐dodecane **7 p** in the InBr_3_‐catalysed process. While such substrates were completely unreactive in the BF_3_ ⋅ OEt_2_‐catalysed process, complete conversion and an excellent yield of **8 p** was obtained when InBr_3_ was applied as catalyst.

After these significant extensions of the scope of the transfer‐hydrogenation reaction from CHD to other alkenes via indium‐based catalysts, we turned our attention towards the selectivity of a regiodiverse hydrodeuterogenation/deuterohydrogenation reaction when indium tribromide is applied as catalyst.

**Scheme 4:**
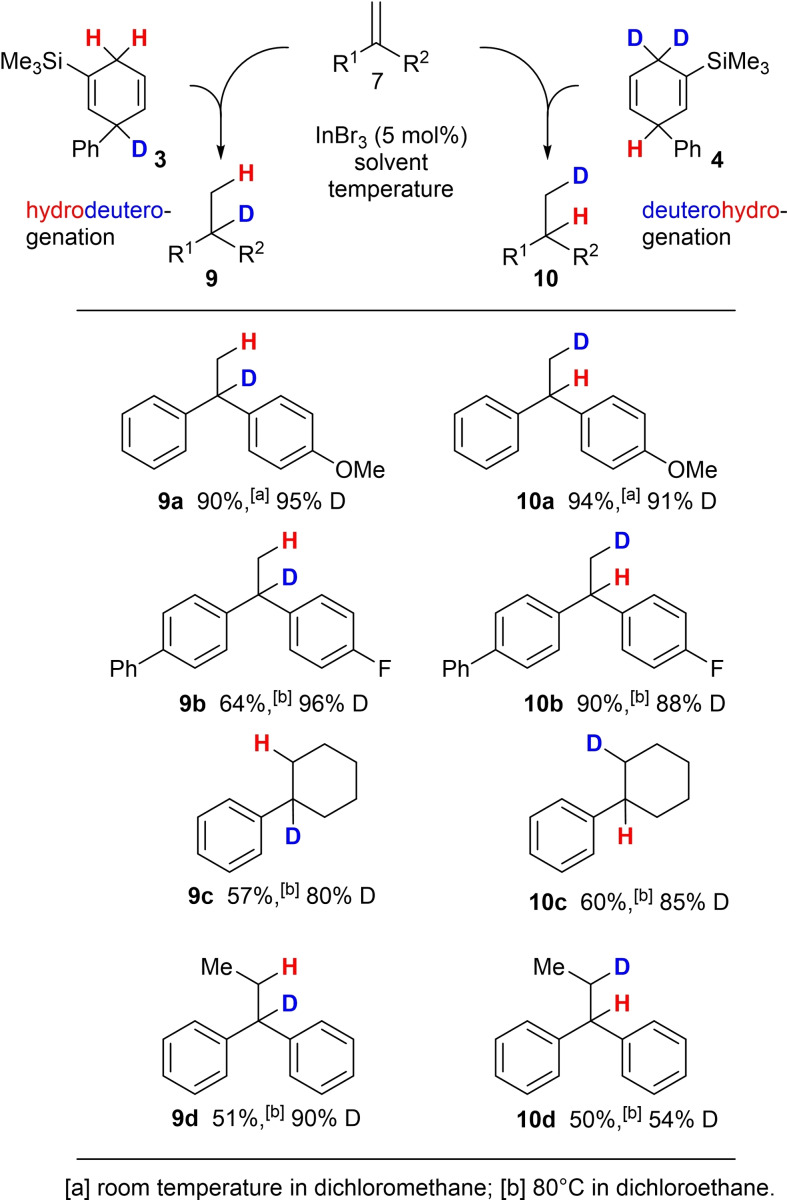
Investigation of the regiodiverse HD/DH addition to selected alkenes utilising the HD surrogates **3** and **4**.

**Scheme 5:**
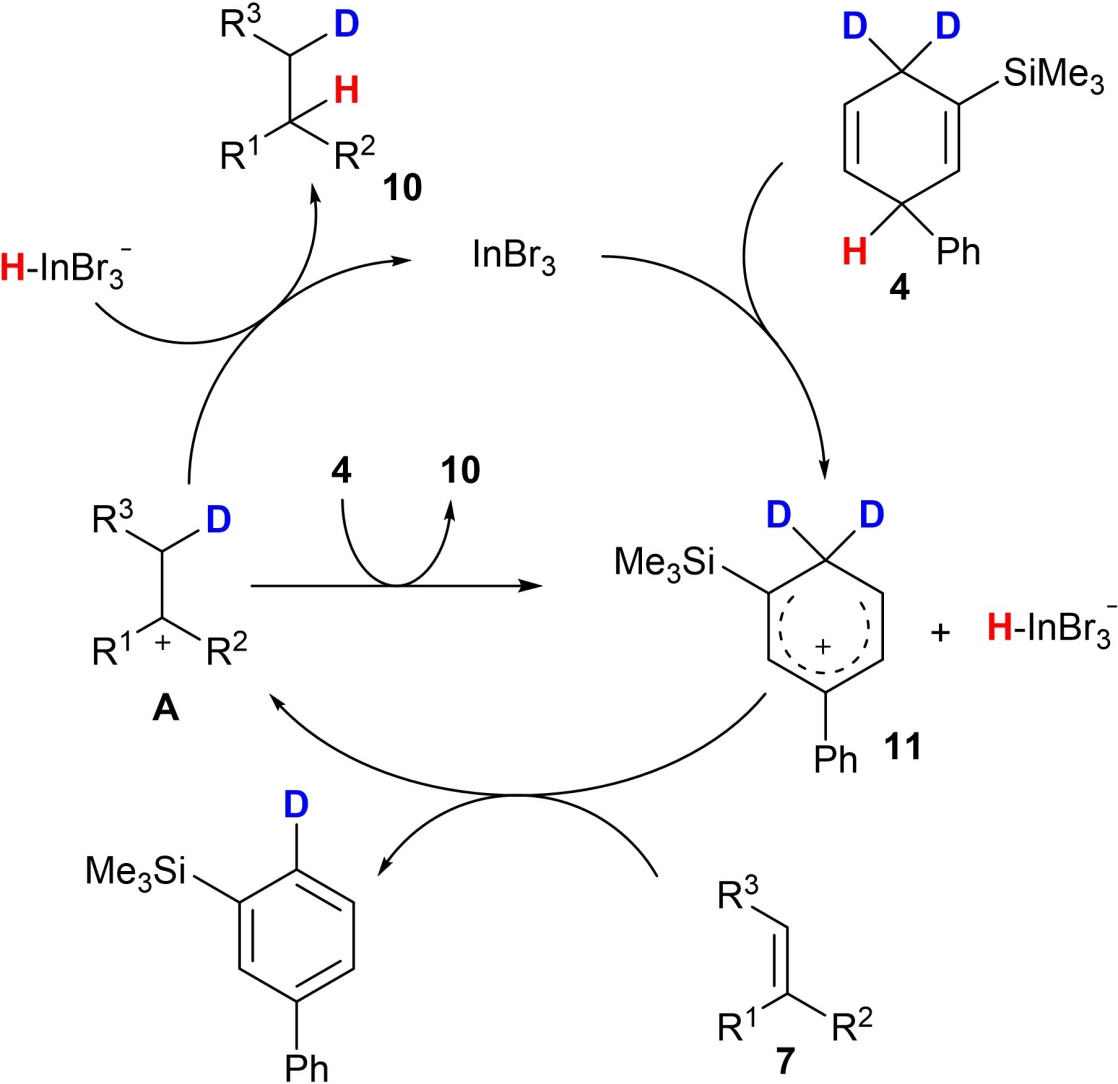
Proposed mechanism of the deuterohydrogenation of alkenes of type **7** utilising **4** as HD surrogate and InBr_3_ as catalyst.

For this purpose, we used the deuterated dihydroaromatic compounds **3** (99 % D incorporation) and **4** (96 % D incorporation, see Scheme 1) to investigate the efficiency of regioselective deuterium transfer to the substrates, outlined in Scheme 4. Similar to the result presented in Scheme 3, the electron‐rich substrate **7 a** showed a good reactivity in the hydrodeuterogenation as well as the deuterohydrogenation with excellent yields and deuterium incorporation at room temperature. The electron‐deficient starting material **7 j** gave product **9 b** with a similar yield compared to **8 j** and an excellent deuterium incorporation, whereas product **10 b** was formed in higher yield. On the other hand, products **9 c** and **10 c** were both formed in reduced yields but acceptable deuterium incorporation. One should not forget that the reactivities of the dihydroaromatic compounds, **3** and **4** for the hydride transfer to InBr_3_ or to intermediate **A** (see Scheme 5) are most likely different, so that the yields may differ from substrate to substrate.

However, the most puzzling result was obtained for product **10 d**. While the deuterium content of **9 d** was still high, only 54 % deuterium incorporation could be detected for **10 d**. Also, small amounts of protodesilylated surrogate **4** (m/z=158) as well as biphenyl (m/z=154) were detected by GCMS analysis of the crude reaction mixture. In the latter case both deuterium atoms of **4** were removed and only in this reaction with this particular substrate.

As proposed by Oestreich and in our previous report, the InBr_3_ acts as Lewis acid and abstracts a hydride anion from the surrogate **4** to initiate the reaction. The Wheland complex **11** acts as strong Brønsted acid and protonates the starting material **7** to afford intermediate **A**. The catalytic cycle is either completed upon reaction of **A** with the HD surrogate **4** or by hydride transfer from [HInBr_3_]^−^. When comparing the results for the synthesis of the deuterium‐labelled compounds **10 c** and **10 d** the loss of 31 % of deuterium‐labelling does not seem to be related to the proposed mechanism. Unfortunately, until now we were not able to identify the proton source which caused the relatively low deuterium labelling in product **10 d**.

## Conclusion

In conclusion, indium tribromide proved to be a suitable Lewis‐acid based catalyst for the transfer‐hydrogenation from dihydroaromatic compounds to alkenes. The scope of the reaction could be significantly expanded not only to electron‐deficient starting materials but also towards alkyl‐substituted alkenes. The hydrodeuterogenation as well as the deuterohydro‐genation with suitable dihydroaromatic compounds could be realised for selected examples in acceptable to good yields and incorporation of deuterium at the desired position of the hydrocarbon product.

## Conflict of interest

The authors declare no conflict of interest.

## Supporting information

As a service to our authors and readers, this journal provides supporting information supplied by the authors. Such materials are peer reviewed and may be re‐organized for online delivery, but are not copy‐edited or typeset. Technical support issues arising from supporting information (other than missing files) should be addressed to the authors.

Supporting Information
