# Supplementary material for: Indium Tribromide‐Catalysed Transfer‐Hydrogenation: Expanding the Scope of the Hydrogenation and of the Regiodivergent DH or HD Addition to Alkenes
Source: Chemistry. 2021 Jun 21;27(43):11221–5. doi: 10.1002/chem.202101259 (PMC8453857; doi:10.1002/chem.202101259)
Supplement: Supplementary file 1 — Supporting Information [file CHEM-27-11221-s001.pdf]

Chemistry–A European Journal

Supporting Information

Indium Tribromide-Catalysed Transfer-Hydrogenation: Expanding the Scope of the Hydrogenation and of the Regiodivergent DH or HD Addition to Alkenes

Luomo Li and Gerhard Hilt*

Content

1 General Information....1
2 Preparation of Deuterated Cyclohexa-1,4-dienes....2
3 Indium Tribromide-Catalysed Transfer-Hydrogenation....5
4 Indium Tribromide-Catalysed Hydrodeuterogenation and Deuterohydrogenation....15
5 References....19
6 Spectra....21

1 General Information

All solvents were purified prior to use by common techniques. All anhydrous solvents were dried over molecular sieve (3 Å) and stored under nitrogen atmosphere. All reactions with air and/or moisture sensitive substances were carried out under a nitrogen atmosphere using standard Schlenk techniques with magnetic stirring. Reagents obtained from commercial sources were used without further purifications.

Non-commercial reagents were prepared according to literature-known procedures. The known substrates **7a**-**7p** were synthesized according to the published methods.[1] The analytical data of each substrate are in accordance with the literature.

Zinc iodide was dried at 200 °C in vacuo and stored under nitrogen atmosphere. The cobalt-based pre-catalyst $CoBr_2$(dppe) was prepared following a literature-known procedure.[2]

Thin layer chromatography (TLC) was carried out on prefabricated plates (silica gel 60, F254 with fluorescence indicator) by Macherey-Nagel and visualised by fluorescence quenching under UV-light. In addition, TLC-plates were stained using a cerium sulfate/phosphomolybdic acid stain ($Ce(SO_4)_2$: 2.0 g, conc. H_2SO_4: 50 mL, $(NH_4)_6Mo_7O_{24}\cdot 4H_2O$: 50 g, H_2O: 400 mL), potassium permanganate stain (K_2CO_3: 60.0 g, $KMnO_4$: 9.0 g, H_2O: 900 mL, acetic acid: 1 mL) or an I_2 stain.

Column chromatography was performed with silica gel 60 M (40-63 µm, 230-400 mesh) from Macherey-Nagel as solid phase with the indicated solvent system.

High resolution mass spectra (EI) were recorded on a Thermo Scientific DFS spectrometer. The ionization was accomplished by electron ionization (EI) at an energy of 70 eV. IR spectra were recorded on a Shimadzu IR Spirit T spectrophotometer equipped with a diamond ATR unit. The absorption bands are given in wave number (cm^{-1}).

^{1}H NMR, ^{19}F NMR and proton decoupled ^{13}C NMR spectra were recorded on *Bruker Fourier* 300HD, or a Bruker Avance III 500HD spectrometer at ambient temperature utilizing pre-set pulse programs. The chemical shifts are given in parts per million

(ppm). NMR standards were used as follows: ^{1}H NMR spectroscopy: δ = 7.26 ppm ($CDCl_3$), δ = 2.50 ppm (DMSO-d_6). ^{19}F NMR spectroscopy: δ = 0.0 ppm ($CFCl_3$). ^{13}C NMR spectroscopy: δ = 77.16 ppm ($CDCl_3$), δ = 39.52 ppm (DMSO-d_6). Data are reported as follows: s = singlet, d = doublet, t = triplet, q = quartet, m = multiplet.

2 Preparation of Deuterated Cyclohexa-1,4-dienes

(*E*)-(4,4-Dideuterobuta-1,3-dien-1-yl)benzene

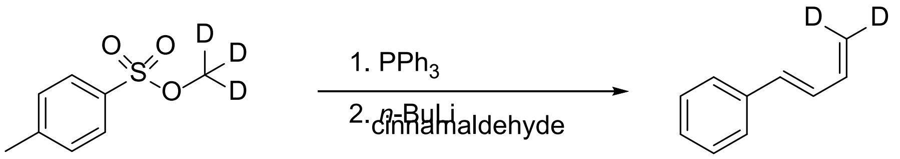

A mixture of triphenylphosphine (4.72 g, 18.0 mmol, 1.0 equiv.) and methyl-d_3 4-methylbenzenesulfonate (3.40 g, 45.1 mmol) was heated to 140 °C for 6 h. Then the mixture was cooled to room temperature, diluted with dry THF (40 mL) and cooled to –78 °C. A solution of *n*-BuLi in hexane (2.5 M, 8.0 mL, 20 mmol, 1.1 equiv.) was added dropwise. The mixture was slowly warmed to room temperature and stirred for 1 h, then cooled to 0 °C. The cinnamaldehyde (2.5 mL, 20 mmol, 1.1 equiv.) was added dropwise. The mixture was warmed to 20 °C and stirred for 20 h. The mixture was filtered through a small plug of silica gel and eluted with diethyl ether. The solvent was evaporated under reduced pressure and the crude product was purified by flash column chromatography on silica gel (*n*-pentane) to afford (*E*)-(4,4-dideuterobuta-1,3-dien-1-yl)benzene (1.59 g, 12.0 mmol, 67%) as a colourless liquid.

R_f = 0.40 (*n*-pentane).

^{1}H NMR (300 MHz, $CDCl_3$): δ = 7.49 – 7.36 (m, 2H), 7.36 – 7.28 (m, 2H), 7.27 – 7.19 (m, 1H), 6.80 (dd, *J* = 15.6, 10.4 Hz, 1H), 6.63 – 6.45 (m, 2H). **^{2}H NMR** (77 MHz, $CDCl_3$): δ = 5.42 (s, 1D), 5.26 (s, 1D) ppm.

The analytical data are in accordance with the literature.[1a]

General Procedure 1: Cyclohexa-1,4-diene Synthesis by the Diels–Alder Reaction

A suspension of $CoBr_2$(dppe) (154 mg, 0.25 mmol, 2.5 mol%), Zn powder (32.5 mg, 0.5 mmol, 10 mol%), and ZnI_2 (319 mg, 0.5 mmol, 5 mol%) in CH_2Cl_2 (5 mL) was charged with the 1,3-diene (5 mmol, 1.0 eq.) and trimethylsilyl acetylene (776 μL, 5.5 mmol, 1.1 equiv.). The reaction mixture was stirred for 5 h. The reaction mixture was purified by flash column chromatography on silica gel (*n*-pentane) to afford the corresponding cyclohexa-1,4-diene.

(1,4-Dihydro-[1,1'-biphenyl]-3-yl)trimethylsilane

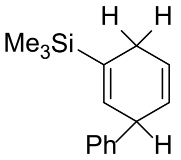

According to the general procedure the title compound was prepared using buta-1,3-dien-1-ylbenzene (651 mg, 5.0 mmol, 1.0 equiv.) and trimethylsilyl acetylene (776 μL, 5.5 mmol, 1.1 equiv.). The product was obtained as pale-yellow liquid (1.03 g, 4.5 mmol, 90%).

$\mathbf{R_f}$ = 0.72 (*n*-pentane).

^{1}H NMR (300 MHz, $CDCl_3$): δ = 7.35 – 7.28 (m, 2H), 7.25 – 7.13 (m, 3H), 6.01 (dt, *J* = 3.8, 2.0 Hz, 1H), 5.88 (dt, *J* = 8.9, 2.7 Hz, 1H), 5.76 (ddd, *J* = 10.1, 3.4, 2.0 Hz, 1H), 4.02 – 3.82 (m, 1H), 2.89 – 2.66 (m, 2H), 0.08 (s, 9H) ppm. **^{13}C NMR** (75 MHz, $CDCl_3$): δ = 145.4, 136.3, 135.1, 128.7, 128.3, 128.2, 126.4, 124.4, 43.0, 27.2, -2.1 ppm.

The analytical data are in accordance with the literature.[1a]

(3-Deutero-3-phenylcyclohexa-1,4-dienyl)trimethylsilane (3)

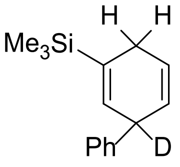

A solution of (1,4-dihydro-[1,1'-biphenyl]-3-yl)trimethylsilane (685 mg, 3.0 mmol, 1.0 equiv.) in dry THF (12 mL) was cooled to −78 °C and *n*-BuLi in hexane (2.5 M, 1.32 mL, 3.3 mmol, 1.1 equiv.) was added. The reaction mixture was slowly warmed to 0 °C and stirred for 2 h. Then the mixture was quenched by the addition of D_2O (0.5 mL, 27.6 mmol, 9.2 equiv.). The mixture was diluted by diethyl ether (12 mL), dried over Na_2SO_4 and filtered. The filtrate was concentrated under reduced pressure. The residue was stirred under air for 1d, and then purified by flash column chromatography on silica gel to afford **4** (344 mg, 1.5 mmol, 50%).

R*f* = 0.72 (*n*-pentane).

^{1}H NMR (300 MHz, $CDCl_3$): δ = 7.37 – 7.30 (m, 2H), 7.25 – 7.13 (m, 3H), 6.01 (q, *J* = 2.0 Hz, 1H), 5.90 (dt, *J* = 10.1, 3.3 Hz, 1H), 5.83 – 5.70 (m, 1H), 2.78 (s, 2H), 0.09 (s, 9H) ppm. **^{2}H NMR** (77 MHz, $CDCl_3$): δ = 3.93 (bs, 1D) ppm. **^{13}C NMR** (75 MHz, $CDCl_3$): δ = 145.4, 136.2, 135.1, 128.7, 128.2, 127.3, 126.4, 124.4, 42.5 (t, *J* = 19.5 Hz), 27.2, -2.1 ppm.

The analytical data are in accordance with the literature.[1a]

(6,6-Dideutero-3-hydro-3-phenylcyclohexa-1,4-dienyl)trimethylsilane (4)

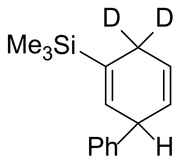

According to the general procedure the title compound was prepared using (*E*)-(4,4-dideuterobuta-1,3-dien-1-yl)benzene (660 mg, 5.0 mmol, 1.0 equiv.) and trimethylsilyl acetylene (776 *μ*L, 5.5 mmol, 1.1 equiv.). The product was obtained as pale-yellow liquid (1.10 g, 4.8 mmol, 96%).

R*f* = 0.72 (*n*-pentane).

^{1}H NMR (500 MHz, $CDCl_3$): δ = 7.33 – 7.29 (m, 2H), 7.24 – 7.17 (m, 3H), 6.00 (dd, *J* = 3.3, 1.9 Hz, 1H), 5.87 (dd, *J* = 10.1, 2.0 Hz, 1H), 5.75 (ddd, *J* = 10.1, 3.3, 2.0 Hz, 1H), 3.92 (s, 1H), 0.07 (s, 9H) ppm. **^{2}H NMR** (77 MHz, $CDCl_3$): δ = 2.75 (2D) ppm. **^{13}C NMR** (126 MHz, $CDCl_3$): δ = 145.4, 136.4, 135.0, 128.7, 128.5, 128.2, 126.4, 124.3, 43.0 ppm. **HRMS** (EI^+) m/z for $C_{15}H_{18}D_2Si[M]^+$: calc: 230.1454, found: 230.1443.

The analytical data are in accordance with the literature.[1a]

3 Indium Tribromide-Catalysed Transfer-Hydrogenation

Preparation of Substrate **7j**

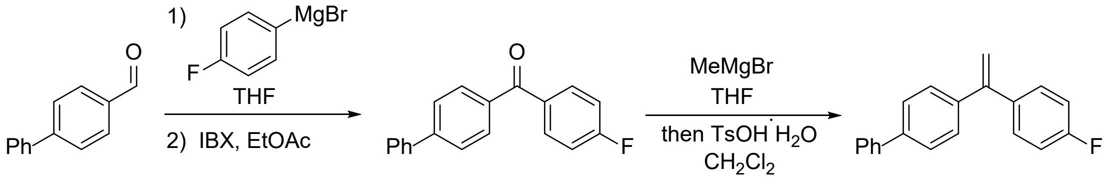

Magnesium (267 mg, 11 mmol, 1.1 equiv.) was added to a 100 mL 3-neck-flask containing 10 mL anhydrous THF and a few crystals of iodine under argon pressure. A solution of 4-fluorobromobenzene (10 mmol, 1.0 eq.) in anhydrous THF (10 mL) was dropped into the suspension of magnesium. After initiation of the reaction, the dropping rate of 4-flouorobromobenzene solution was adjusted to maintain the reaction system refluxing. After the dropwise addition, heating of the mixture continued to reflux for 2 h. The reaction mixture was cooled to 0 °C before the [1,1'-biphenyl]-4-carbaldehyde was added dropwise. Then the mixture was heated to reflux for 10 h. After the mixture was cooled to room temperature, the mixture was quenched with water (20 mL). The mixture was extracted with ethyl acetate (3 × 20 mL). The combined organic layer was washed with brine, dried over Na_2SO_4 and filtered. The filtrate was concentrated under reduced pressure.

Then the residue was diluted with ethyl acetate (20 mL). IBX (3.08 g, 11 mmol, 1.1 equiv.) was added. The mixture was heated to 80 °C and stirred at this temperature for 20 h before the mixture was filtered. The filtrate was concentrated under reduced pressure to afford the product [1,1'-biphenyl]-4-yl(4-fluorophenyl)methanone (1.96 g, 7.1 mmol, 71%) as colourless solid.

R*f* = 0.20 (*n*-pentane:diethyl ether = 20:1).

^{1}H NMR (500 MHz, $CDCl_3$): δ = 7.92 – 7.83 (m, 4H), 7.75 – 7.69 (m, 2H), 7.68 – 7.63 (m, 2H), 7.50 – 7.47 (m, 2H), 7.45 – 7.39 (m, 1H), 7.23 – 7.14 (m, 2H) ppm. **^{13}C NMR** (126 MHz, $CDCl_3$): δ = 194.9, 165.5 (d, *J* = 254.1 Hz), 145.4, 140.0, 136.3, 134.1 (d, *J* = 3.0 Hz), 132.7 (d, *J* = 9.1 Hz), 130.6, 129.1, 128.3, 127.4, 127.1, 115.6 (d, *J* = 21.9 Hz) ppm. **^{19}F NMR** (470 MHz, $CDCl_3$): δ = -106.11 ppm.

IR (ATR, neat): $\tilde{\nu}$ = 1640, 1596, 1557, 1502, 1484, 1449, 1403, 1343, 1317, 1304, 1284, 1226, 1182, 1149, 1096, 1013, 1004, 961, 956, 930, 859, 841, 814, 783, 756, 744, 723, 690, 677, 677, 663, 639, 629, 614, 601, 563, 531, 507 cm^{-1}.

HRMS: (EI^+) m/z for $C_{19}H_{13}FO$ [M^+]: calc.: 276.0945, found: 276.0941.

Under nitrogen atmosphere [1,1'-biphenyl]-4-yl(4-fluorophenyl)methanone (1.38 g, 5.0 mmol, 1.0 equiv.) was dissolved in 10.0 mL THF. The solution was cooled to 0 °C and a solution of methyl magnesium bromide (3.0 M in diethyl ether, 2.0 mL, 6.0 mmol, 1.2 equiv.) was added dropwise. Then the mixture was warmed slowly to room temperature and stirred for 20 h before it was quenched by 20 mL sat. NH_4Cl. The phases were separated. The aqueous phase was extracted by 3 × 20 mL diethyl ether. Then the combined organic phase was dried over Na_2SO_4 and filtered. The filtrate was concentrated under reduced pressure. The residue was dissolved in 20 mL CH_2Cl_2. To this solution TsOH·H_2O (396 mg, 2.0 mmol, 40 mol%) was added and the mixture was stirred until the reaction was complete (monitored by GC-MS). The mixture was washed with 20 mL sat. $NaHCO_3$, and dried over Na_2SO_4, concentrated under reduced pressure. The crude product was purified by flash column chromatography to afford the product 4-(1-(4-fluorophenyl)vinyl)-1,1'-biphenyl (1.18 g, 4.3 mmol, 86%) as light yellow solid.

$\mathbf{R}_f$ = 0.70 (*n*-pentane:diethyl ether = 20:1).

^{1}H NMR (500 MHz, $CDCl_3$): δ = 7.65 – 7.61 (m, 2H), 7.60 – 7.56 (m, 2H), 7.48 – 7.44 (m, 2H), 7.44 – 7.38 (m, 2H), 7.39 – 7.33 (m, 3H), 7.10 – 7.01 (m, 2H), 5.52 (d, J = 1.1 Hz, 1H), 5.44 (d, J = 1.1 Hz, 1H) ppm. **^{13}C NMR** (126 MHz, $CDCl_3$): δ = 162.7 (d, *J* = 247 Hz) 148.9, 140.9, 140.8, 140.4, 137.7 (d, *J* = 3.3 Hz), 137.7, 130.1 (d, *J* = 8.0 Hz), 129.0, 128.7, 127.5, 127.2, 127.1, 115.2 (d, *J* = 21.4 Hz), 114.3 ppm. ^{19}F NMR (470 MHz, $CDCl_3$) δ = -114.65 ppm.

IR (ATR, neat): $\tilde{\nu}$ = 3033, 1657, 1597, 1504, 1486, 1447, 1403, 1327, 1274, 1222, 1156, 1096, 1074, 1013, 1004, 929, 900, 846, 804, 771, 744, 730, 690, 664, 638, 610, 601, 580, 559, 521 cm^{-1}.

HRMS: (EI^+) m/z for $C_{20}H_{15}F$ [M^+]: calc.: 274.1152, found: 274.1149.

General Procedure 2: Indium Tribromide-Catalysed Transfer-Hydrogenation

A sealed tube was charged with a stir bar and indium bromide (17.7 mg, 0.05 mmol, 5 mol%) and heat to 80 °C for 30 min under vacuum, then flushed with nitrogen and cooled to room temperature. Then 1 mL DCE or CH_2Cl_2, the corresponding alkene (1 mmol, 1.0 equiv.) and 1,4-cyclohexadiene (104 μL, 1.1 mmol, 1.1 equiv.) were added in sequence. The mixture was heated to the appropriate temperature and the progress of the reaction was monitored by GC-MS. Then the reaction mixture was purified directly by flash column chromatography (n-pentane to *n*-pentane:diethyl ether = 10:1) to afford the pure product.

Ethane-1,1-diyldibenzene (6)

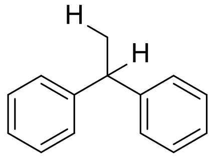

According to the general procedure 2 the title compound was prepared using ethene-1,1-diyldibenzene (177 μL, 1.0 mmol, 1.0 equiv.) at 80 °C in DCE. The product was obtained as a colourless liquid (177 mg, 0.97 mmol, 97%).

R*f* = 0.35 (*n*-pentane).

^{1}H NMR (300 MHz, $CDCl_3$): δ = 7.69 – 6.89 (m, 10H), 4.20 (q, J = 7.2 Hz, 1H), 1.69 (d, J = 7.3 Hz, 3H) ppm. **^{13}C NMR** (75 MHz, $CDCl_3$): δ = 146.5, 128.5, 127.8, 126.2, 44.9, 22.0 ppm.

The analytical data are in accordance with the literature.[1a]

1-Methoxy-4-(1-phenylethyl)benzene (8a)

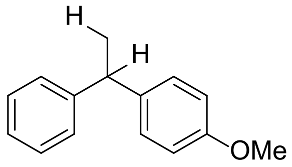

According to the general procedure 2 the title compound was prepared using 1-methoxy-4-(1-phenylvinyl)benzene (210 mg, 1.0 mmol, 1.0 equiv.) at rt in CH_2Cl_2. The product was obtained as a colourless liquid (199 mg, 0.94 mmol, 94%).

R*f* = 0.74 (*n*-pentane:diethyl ether = 20:1).

^{1}H NMR (500 MHz, $CDCl_3$): δ = 7.30 – 7.23 (m, 2H), 7.22 – 7.18 (m, 2H), 7.17 – 7.10

(m, 3H), 6.85 – 6.77 (m, 2H), 4.09 (q, *J* = 7.2 Hz, 1H), 3.76 (s, 3H), 1.60 (d, *J* = 7.2 Hz, 3H) ppm. **^{13}C NMR** (126 MHz, $CDCl_3$): δ = 158.0, 146.9, 138.7, 128.6, 128.5, 127.7, 126.1, 113.9, 77.4, 77.2, 76.9, 55.4, 44.1, 22.2 ppm.

The analytical data are in accordance with the literature.[1a]

1-Methoxy-2-(1-phenylethyl)benzene(8b)

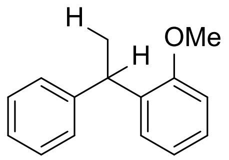

According to the general procedure 2 the title compound was prepared using ethene-1-methoxy-2-(1-phenylvinyl)benzene (210 mg, 1.0 mmol, 1.0 equiv.) at rt in CH_2Cl_2. The product was obtained as a colourless liquid (203 mg, 0.96 mmol, 96%).

R*f* = 0.22 (*n*-pentane).

^{1}H NMR (500 MHz, $CDCl_3$): δ = 7.31 – 7.22 (m, 4H), 7.21 – 7.12 (m, 3H), 6.92 (td, *J* = 7.5, 1.2 Hz, 1H), 6.85 (dd, *J* = 8.1, 1.1 Hz, 1H), 4.59 (q, *J* = 7.3 Hz, 1H), 3.78 (s, 3H), 1.59 (d, J = 7.3 Hz, 3H) ppm. **^{13}C NMR** (126 MHz, $CDCl_3$): δ = 157.0, 146.6, 135.1, 128.2, 127.9, 127.8, 127.2, 125.8, 120.7, 110.8, 55.6, 37.59, 21.0 ppm.

The analytical data are in accordance with the literature.[1a]

1-Methoxy-3-(1-phenylethyl)benzene (8c)

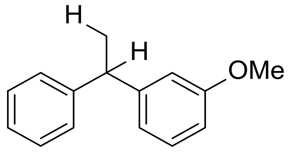

According to the general procedure 2 the title compound was prepared using ethene-1-methoxy-3-(1-phenylvinyl)benzene (210 mg, 1.0 mmol, 1.0 equiv.) at 80 °C in DCE. The product was obtained as a pale-yellow liquid (158 mg, 0.74 mmol, 74%).

R*f* = 0.10 (*n*-pentane).

^{1}H NMR (500 MHz, $CDCl_3$): δ = 7.31 – 7.27 (m, 2H), 7.25 – 7.16 (m, 4H), 6.83 (ddt, *J* = 7.6, 1.5, 0.8 Hz, 1H), 6.79 (t, *J* = 2.2 Hz, 1H), 6.74 (ddd, *J* = 8.2, 2.6, 0.9 Hz, 1H), 4.13 (q, *J* = 7.2 Hz, 1H), 3.78 (s, 3H), 1.65 (d, J = 7.2 Hz, 3H) ppm. **^{13}C NMR** (126 MHz, $CDCl_3$): δ = 159.8, 148.2, 146.3, 129.4, 128.5, 127.7, 126.2, 120.3, 114.0, 111.1, 55.3, 45.0, 21.9 ppm.

The analytical data are in accordance with the literature.[1a]

1,2-Dimethoxy-4-(1-phenylethyl)benzene (8d)

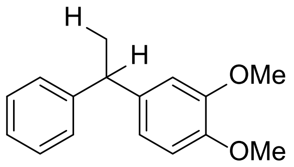

According to the general procedure 2 the title compound was prepared using ethene-1,2-dimethoxy-4-(1-phenylvinyl)-benzene (240 mg, 1.0 mmol, 1.0 equiv.) at rt in CH_2Cl_2. The product was obtained as a dull red liquid (35.9 mg, 0.15 mmol, 15%).

R*f* = 0.15 (*n*-pentane:diethyl ether = 20:1).

^{1}H NMR (500 MHz, $CDCl_3$): δ = 7.38 – 7.13 (m, 5H), 6.90 – 6.67 (m, 3H), 4.15 (q, J = 7.3 Hz, 1H), 3.90 (s, 3H), 3.87 (s, 3H), 1.67 (d, *J* = 7.2 Hz, 3H) ppm. **^{13}C NMR** (126 MHz, $CDCl_3$): δ = 149.2, 147.8, 147.0, 139.4, 128.8, 127.9, 126.4, 119.8, 111.7, 111.5, 56.3, 56.3, 44.8, 22.5 ppm.

The analytical data are in accordance with the literature.[3]

4,4'-(ethane-1,1-diyl)bis(methoxybenzene) (8e)

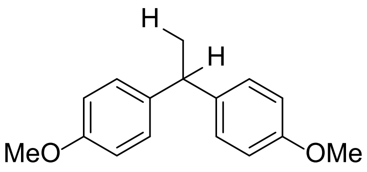

According to the general procedure 2 the title compound was prepared using 4,4'-(ethene-1,1-diyl)bis(methoxybenzene) (242 mg, 1.0 mmol, 1.0 equiv.) at rt in CH_2Cl_2. The product was obtained as a colourless solid (214 mg, 0.88 mmol, 88%).

R*f* = 0.52 (*n*-pentane:diethyl ether = 20:1).

^{1}H NMR (300 MHz, $CDCl_3$): δ = 7.20 – 6.96 (m, 4H), 6.96 – 6.66 (m, 4H), 4.06 (q, *J* = 7.3 Hz, 1H), 3.78 (s, 6H), 1.59 (d, *J* = 7.2 Hz, 3H) ppm. **^{13}C NMR** (126 MHz, $CDCl_3$): δ = 157.9, 139.1, 128.5, 113.8, 55.4, 55.3, 43.2, 22.4 ppm.

The analytical data are in accordance with the literature.[1a]

4,4'-(Ethane-1,1-diyl)bis(fluorobenzene) (8f)

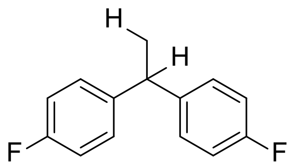

According to the general procedure 2 the title compound was prepared using ethene-4,4'-(ethene-1,1-diyl)bis(fluorobenzene) (216 mg, 1.0 mmol, 1.0 equiv.) at 120 °C in DCE. The product was obtained as a colourless liquid (185 mg, 0.85 mmol, 85%).

R*f* = 0.47 (*n*-pentane:diethyl ether = 20:1)

^{1}H NMR (500 MHz, $CDCl_3$): δ = 7.19 – 7.10 (m, 4H), 7.02 – 6.93 (m, 4H), 4.12 (q, *J* = 7.2 Hz, 1H), 1.59 (s, 3H). **^{13}C NMR** (126 MHz, $CDCl_3$): δ = 162.5 (d, *J* = 224.1 Hz), 142.0 (d, *J* = 2.8 Hz), 129.0 (d, *J* = 7.9 Hz), 115.3 (d, *J* = 21.2 Hz), 43.5, 22.3. **^{19}F NMR** (470 MHz, $CDCl_3$): δ = -117.24 ppm.

The analytical data are in accordance with the literature.[4]

4,4'-(Ethane-1,1-diyl)bis(chlorobenzene) (8g)

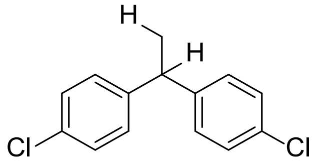

According to the general procedure 2 the title compound was prepared using ethene-4,4'-(ethene-1,1-diyl)bis(chlorobenzene) (249 mg, 1.0 mmol, 1.0 equiv.) at 120 °C in DCE. The product was obtained as a colourless solid (216 mg, 0.86 mmol, 86%).

R*f* = 0.50 (*n*-pentane).

^{1}H NMR (500 MHz, $CDCl_3$): δ = 7.31 – 7.24 (m, 4H), 7.17 – 7.10 (m, 4H), 4.12 (q, *J* = 7.2 Hz, 1H), 1.62 (d, *J* = 7.2 Hz, 3H) ppm. **^{13}C NMR** (75 MHz, $CDCl_3$): δ = 144.4, 132.1, 129.0, 128.7, 43.7, 21.9 ppm.

The analytical data are in accordance with the literature.[5]

1-Bromo-4-(1-phenylethyl)benzene (8h)

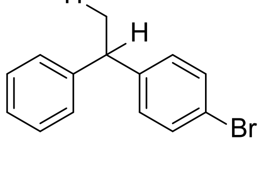

According to the general procedure 2 the title compound was prepared using ethene-1-bromo-4-(1-phenylvinyl)benzene (259 mg, 1.0 mmol, 1.0 equiv.) at 120 °C in DCE. The product was obtained as a colourless liquid (178 mg, 0.68 mmol, 68%).

R*f* = 0.44 (*n*-pentane).

^{1}H NMR (500 MHz, $CDCl_3$): δ = 7.46 – 7.37 (m, 2H), 7.35 – 7.26 (m, 2H), 7.25 – 7.16 (m, 3H), 7.13 – 7.08 (m, 2H), 4.12 (q, *J* = 7.2 Hz, 1H), 1.63 (dd, *J* = 7.2, 1.2 Hz, 3H) ppm. **^{13}C NMR** (126 MHz, $CDCl_3$): δ = 145.8, 145.5, 131.6, 129.5, 128.6, 127.7, 126.4, 120.0, 44.4, 21.9 ppm.

The analytical data are in accordance with the literature.[4]

1-(1-Phenylethyl)-4-(trifluoromethyl)benzene (8i)

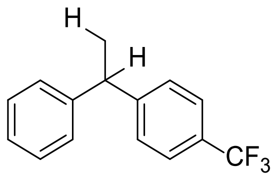

According to the general procedure 2 the title compound was prepared using ethene-1-(1-phenylvinyl)-4-(trifluoromethyl)-benzene (248 mg, 1.0 mmol, 1.0 equiv.) at 100 °C in DCE. The product was obtained as a pale-yellow liquid (168 mg, 0.67 mmol, 67%).

R*f* = 0.44 (*n*-pentane).

^{1}H NMR (500 MHz, $CDCl_3$): δ = 7.57 – 7.54 (m, 2H), 7.37 – 7.29 (m, 4H), 7.25 – 7.18 (m, 3H), 4.22 (q, *J* = 7.2 Hz, 1H), 1.67 (d, *J* = 7.2 Hz, 3H) ppm. **^{13}C NMR** (126 MHz, $CDCl_3$): δ = 150.6, 145.4, 128.7, 128.1 (q, *J* = 33 Hz), 128.1, 127.7, 126.6, 125.5 (q, *J* = 3.6 Hz), 44.8, 21.8 ppm. The signal at 124.5 ppm for the CF_3 carbon was not detectable. **^{19}F NMR** (470 MHz, $CDCl_3$): δ = -62.38 ppm.

The analytical data are in accordance with the literature.[6]

4-(1-(4-Fluorophenyl)ethyl)-1,1'-biphenyl (8j)

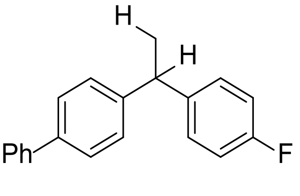

According to the general procedure 2 the title compound was prepared using 4-(1-(4-fluorophenyl)vinyl)-1,1'-biphenyl (274 mg, 1.0 mmol, 1.0 equiv.) at 80 °C in DCE. The product was obtained as a colourless liquid (178 mg, 0.64 mmol, 64%).

R*f* = 0.76 (*n*-pentane:diethyl ether = 20:1).

^{1}H NMR (500 MHz, $CDCl_3$): δ = 7.61 – 7.57 (m, 2H), 7.57 – 7.52 (m, 2H), 7.44 (dd, *J* = 8.4, 6.9 Hz, 2H), 7.36 – 7.32 (m, 1H), 7.31 – 7.27 (m, 2H), 7.26 – 7.21 (m, 2H), 7.03 – 6.98 (m, 2H), 4.20 (q, *J* = 7.2 Hz, 1H), 1.68 (d, *J* = 7.2 Hz, 3H) ppm. **^{13}C NMR**

(126 MHz, $CDCl_3$): δ = 161.5 (d, *J* = 3.1 Hz), 145.4, 142.1 (d, *J* = 224.1 Hz), 141.1, 139.3, 129.2 (d, *J* = 7.8 Hz), 128.9, 128.1, 127.3, 127.3, 127.2, 115.3 (d, *J* = 21.1 Hz), 43.92, 22.18 ppm. **^{19}F NMR** (470 MHz, $CDCl_3$): δ = -117.33 ppm.

IR (ATR, neat): $\tilde{\nu}$ = 3030, 1597, 1504, 1484,1446, 1403, 1236, 1216, 1160, 1153, 1113, 1074, 1009, 999, 907, 841, 830, 823, 796, 764, 739, 721, 694, 604, 599, 561, 553, 504 cm^{-1}.

HRMS (EI^+) m/z for $C_{20}H_{17}F$ [M^+]: calc.: 276.1305, found: 276.1301.

4-(1-Phenylethyl)benzaldehyde (8k)

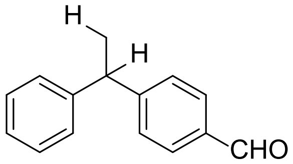

According to the general procedure 2 the title compound was prepared using ethene-4-(1-phenylvinyl)benzaldehyde (208 mg, 1.0 mmol, 1.0 equiv.) at 80 °C in DCE. The product was obtained as a pale-yellow solid (111 mg, 0.53 mmol, 53%).

R*f* = 0.20 (*n*-pentane:diethyl ether = 20:1).

^{1}H NMR (500 MHz, $CDCl_3$): δ = 9.97 (s, 1H), 7.83 – 7.77 (m, 2H), 7.41 – 7.37 (m, 2H), 7.33 – 7.29 (m, 2H), 7.24 – 7.20 (m, 3H), 4.24 (q, *J* = 7.2 Hz, 1H), 1.68 (d, *J* = 7.2 Hz, 3H) ppm. **^{13}C NMR** (126 MHz, $CDCl_3$): δ = 192.0, 153.8, 145.2, 134.8, 130.1, 128.7, 128.5, 127.7, 126.6, 45.2, 21.7 ppm.

The analytical data are in accordance with the literature.[7]

Cyclohexylbenzene (8l)

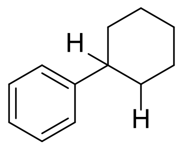

According to the general procedure 2 the title compound was prepared using ethene-2,3,4,5-tetrahydro-1,1'-biphenyl (158 mg, 1.0 mmol, 1.0 equiv.) at 80 °C in DCE. The product was obtained as a colourless liquid (145 mg, 0.90 mmol, 90%).

R*f* = 0.74 (*n*-pentane).

^{1}H NMR (500 MHz, $CDCl_3$): δ = 7.31 – 7.27 (m, 2H), 7.24 – 7.20 (m, 2H), 7.20 – 7.16 (m, 1H), 2.51 (tt, *J* = 11.5, 3.4 Hz, 1H), 1.87 (ddt, *J* = 21.3, 9.5, 3.2 Hz, 4H), 1.76 (dqd, *J* = 12.7, 3.0, 1.5 Hz, 1H), 1.48 – 1.36 (m, 4H), 1.32 – 1.22 (m, 1H) ppm. **^{13}C NMR** (126 MHz, $CDCl_3$): δ = 148.2, 128.4, 127.0, 125.9, 44.8, 34.6, 27.1, 26.4 ppm.

The analytical data are in accordance with the literature.[8]

Phenylcyclooctane (8m)

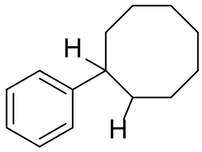

According to the general procedure 2 the title compound was prepared using 1-phenylcyclooct-1-ene (186 mg, 1.0 mmol, 1.0 equiv.) at 80 °C in DCE. The product was obtained as a colourless liquid (169 mg, 0.90 mmol, 90%).

R*f* = 0.74 (*n*-pentane).

^{1}H NMR (500 MHz, $CDCl_3$): δ = 7.33 – 7.27 (m, 2H), 7.24 – 7.15 (m, 3H), 2.79 (tt, *J* = 9.7, 3.6 Hz, 1H), 1.92 – 1.85 (m, 2H), 1.85 – 1.75 (m, 4H), 1.73 – 1.67 (m, 3H), 1.67 – 1.55 (m, 5H) ppm. **^{13}C NMR** (126 MHz, $CDCl_3$): δ = 150.6, 128.4, 127.1, 125.6, 44.8, 34.8, 27.1, 26.5, 26.2 ppm.

The analytical data are in accordance with the literature.[9]

Propane-1,1-diyldibenzene (8n)

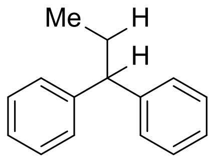

According to the general procedure 2 the title compound was prepared using ethene-prop-1-ene-1,1-diyldibenzene (194 mg, 1.0 mmol, 1.0 equiv.) at 80 °C in DCE. The product was obtained as a colourless liquid (144 mg, 0.73 mmol, 73%).

R*f* = 0.32 (*n*-pentane).

^{1}H NMR (500 MHz, $CDCl_3$): δ = 7.39 – 7.27 (m, 8H), 7.27 – 7.17 (m, 2H), 3.84 (t, *J* = 7.8 Hz, 1H), 2.13 (p, *J* = 7.3 Hz, 2H), 0.96 (t, *J* = 7.3 Hz, 3H). **^{13}C NMR** (126 MHz, $CDCl_3$): δ = 145.3, 128.5, 128.1, 126.1, 53.4, 28.7, 12.9 ppm.

The analytical data are in accordance with the literature.[10]

1-Methyl-1,2,3,4-tetrahydronaphthalene (8o)

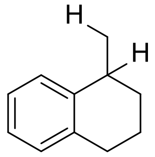

According to the general procedure 2 the title compound was prepared using 1-methylene-1,2,3,4-tetrahydronaphthalene (144 mg, 1.0 mmol, 1.0 equiv.) at 80 °C in DCE. The product was obtained as a colourless

liquid (78.0 mg, 0.53 mmol, 53%).

R*f* = 0.50 (*n*-pentane).

^{1}H NMR (500 MHz, $CDCl_3$): δ = 7.39 – 7.28 (m, 1H), 7.26 (s, 3H), 3.09 – 2.94 (m, 1H), 2.93 – 2.76 (m, 2H), 2.14 – 1.72 (m, 3H), 1.71 – 1.55 (m, 1H), 1.40 (d, J = 7.0 Hz, 3H) ppm. **^{13}C NMR** (75 MHz, $CDCl_3$): δ = 142.3, 137.0, 129.1, 128.2, 125.7, 125.5, 32.6, 31.6, 30.1, 23.0, 20.6 ppm.

The analytical data are in accordance with the literature.[11]

3-Methyldodecane (8p)

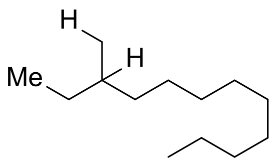

According to the general procedure 2 the title compound was prepared using ethene-3-methyleneundecane (168 mg, 1.0 mmol, 1.0 equiv.) at 80 °C in DCE. The product was obtained as a colourless liquid (173 mg, 1.0 mmol, 99%).

R*f* = 0.90 (*n*-pentane).

^{1}H NMR (500 MHz, $CDCl_3$): δ = 1.38 – 1.19 (m, 17H), 0.94 – 0.79 (m, 9H) ppm. **^{13}C NMR** (126 MHz, $CDCl_3$): δ = 36.8, 34.6, 32.1, 30.2, 29.9, 29.7, 29.5, 27.3, 22.9, 19.4, 14.3, 11.6 ppm.

The analytical data are in accordance with the literature.[12]

4 Indium Tribromide-Catalysed Hydrodeuterogenation and Deuterohydrogenation

General Procedure 3

A sealed tube was charged with a stir bar and indium tribromide (5.3 mg, 0.015 mmol, 5 mol%) and heated to 80 °C for 30 min under vacuum, then flushed with nitrogen and cooled to room temperature. 1.0 mL DCE or CH_2Cl_2, alkene (0.3 mmol, 1.0 equiv.) and corresponding deuterated 1,4-cyclohexadiene (0.39 mmol, 1.3 equiv.) was added in sequence. The reaction mixture was heated to the corresponding temperature and the mixture was stirred until the reaction was complete (monitored by GC-MS or TLC). Then the reaction mixture was purified directly by flash column chromatography (*n*-pentane to n-pentane:diethyl ether = 10:1) to afford the title compound.

1-Methoxy-4-(1-phenylethyl-1-deutero)benzene (9a)

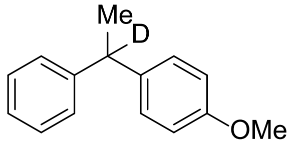

According to the general procedure 3 the title compound was prepared using 1-methoxy-4-(1-phenylvinyl)benzene (63.1 mg, 0.3 mmol, 1.0 equiv.) and (3-deutero-3-phenyl-cyclohexa-1,4-dienyl)trimethylsilane in CH_2Cl_2 at rt. The product was obtained as colourless solid.

R*f* = 0.74 (*n*-pentane:diethyl ether = 20:1).

^{1}H NMR (500 MHz, $CDCl_3$): δ = 7.31 – 7.26 (m, 2H), 7.24 – 7.20 (m, 2H), 7.20 – 7.16 (m, 1H), 7.16 – 7.11 (m, 2H), 6.99 – 6.69 (m, 2H), 3.79 (s, 3H), 1.62 (s, 3H) ppm. **^{2}H NMR** (77 MHz, $CHCl_3$:$CDCl_3$ = 20:1): δ = 4.12 (bs, 1D) ppm. **^{13}C NMR** (126 MHz, $CDCl_3$): δ = 158.0, 146.9, 138.7, 128.6, 128.5, 127.7, 126.1, 113.9, 55.4, 43.7 (t, *J* = 19.4), 22.1 ppm.

HRMS (EI^+) m/z for $C_{16}H_{17}{}^2HO_2$ [M^+]: calc.: 243.1364, found: 243.1368.

The analytical data are in accordance with the literature.[1a]

4-(1-(4-Fluorophenyl)ethyl-1-deutero)-1,1'-biphenyl (9b)

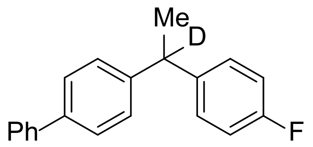

According to the general procedure 3 the title compound was prepared using 1-methoxy-4-(1-phenylvinyl)benzene (63.1 mg, 0.3 mmol, 1.0 equiv.) and (3-deutero-3-phenyl-cyclohexa-1,4-dienyl)trimethylsilane in DCE at 80 °C. The product was obtained as a colourless solid.

R*f* = 0.76 (*n*-pentane:diethyl ether = 20:1).

^{1}H NMR (500 MHz, $CDCl_3$): δ 7.66 – 7.60 (m, 2H), 7.60 – 7.52 (m, 2H), 7.47 (dd, J = 8.4, 7.0 Hz, 2H), 7.41 – 7.35 (m, 1H), 7.35 – 7.30 (m, 2H), 7.29 – 7.23 (m, 2H), 7.11 – 6.96 (m, 2H), 1.70 (s, 3H) ppm. **^{2}H NMR** (77 MHz, $CHCl_3$:$CDCl_3$ = 20:1): δ = 4.18 (bs, 1D) ppm. **^{13}C NMR** (126 MHz, $CDCl_3$): δ = 162.5 (d, *J* = 244.1 Hz), 160.5, 145.4, 142.6 (d, *J* = 3.0 Hz), 141.1, 139.3, 129.1 (d, *J* = 7.8 Hz), 128.9, 128.0, 127.32, 127.30, 127.2, 115.3 (t, *J* = 21.1 Hz), 43.9, 43.5 (t, *J* = 19.6 Hz), 22.1 ppm.

HRMS (EI^+) m/z for $C_{20}H_{16}{}^2HF$ [M^+]: calc.: 277.1372, found: 277.1369.

(Cyclohexyl-1-deutero)benzene (9c)

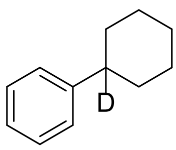

According to the general procedure 3 the title compound was prepared using 2,3,4,5-tetrahydro-1,1'-biphenyl (47.5 mg, 0.3 mmol, 1.0 equiv.) and (3-deutero-3-phenylcyclo-hexa-1,4-dienyl)trimethylsilane in DCE at 80 °C. The product was obtained as a colourless liquid.

R*f* = 0.74 (*n*-pentane).

^{1}H NMR (500 MHz, $CDCl_3$): δ = 7.33 – 7.27 (m, 2H), 7.25 – 7.13 (m, 3H), 2.07 – 1.81 (m, 4H), 1.77 (dddd, *J* = 13.8, 6.1, 3.7, 2.0 Hz, 1H), 1.49 – 1.15 (m, 5H) ppm. **^{2}H NMR** (77 MHz, $CHCl_3$:$CDCl_3$ = 20:1): δ = 2.50 (bs, 1D) ppm. **^{13}C NMR** (126 MHz, $CDCl_3$): δ = 148.2, 128.4, 127.0, 125.9, 44. 8, 44.3 (t, *J* = 19.1 Hz), 34.7, 34.6, 27.1, 26.4 ppm.

HRMS (EI^+) m/z for $C_{12}H_{15}{}^2H$ [M^+]: calc.: 161.1309, found: 161.1307.

(Propane-1,1-diyl-1- deutero)dibenzene (9d)

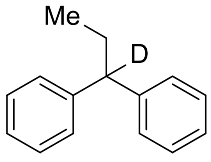

According to the general procedure 3 the title compound was prepared using 1-methoxy-4-(1-phenylvinyl)benzene (63.1 mg, 0.3 mmol, 1.0 equiv.) and (3-deutero-3-phenylcyclohexa-1,4-dienyl)trimethylsilane in DCE at 80 °C. The product was obtained as a colourless liquid.

R*f* = 0.32 (*n*-pentane).

^{1}H NMR (500 MHz, $CDCl_3$): δ = 7.42 – 7.20 (m, 8H), 7.19 – 7.11 (m, 2H), 2.08 (q, *J* = 7.4 Hz, 2H), 0.91 (t, *J* = 7.3 Hz, 3H) ppm. **^{2}H NMR** (77 MHz, $CHCl_3$:$CDCl_3$ = 20:1): δ = 3.80 (bs, 1D) ppm. **^{13}C NMR** (126 MHz, $CDCl_3$): δ = 145.3, 128.5, 128.1, 126.2, 53.0 (t, *J* = 19.4 Hz), 28.7, 12.9 ppm. An additional signal was observed at 53.4 ppm for CH-Et derivative.

HRMS (EI$^+$) m/z for $C_{15}H_{15}{}^2H$ [M^+]: calc.: 197.1309, found: 197.1311.

1-Methoxy-4-(2-deutero-1-phenylethyl)benzene (10a)

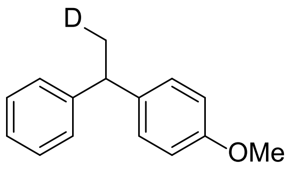

According to the general procedure 3 the title compound was prepared using 1-methoxy-4-(1-phenylvinyl)benzene (63.1 mg, 0.3 mmol, 1.0 equiv.) and 6,6-dideutero-3-hydro-3-phenylcyclohexa-1,4-dienyl)trimethylsilane in CH_2Cl_2 at rt. The product was obtained as a pale-yellow liquid (60.1 mg, 0.28 mmol, 94%).

R*f* = 0.74 (*n*-pentane:diethyl ether = 20:1).

^{1}H NMR (500 MHz, DMSO-*d$_6$*): δ = 7.49 – 7.05 (m, 7H), 7.05 – 6.61 (m, 2H), 4.30 – 3.96 (t, *J* = 7.1 Hz, 1H), 3.96 – 3.59 (m, 3H), 1.67 – 1.55 (t, *J* = 7.2 Hz, 2H) ppm. **^{2}H NMR** (77 MHz, $CHCl_3$:$CDCl_3$ = 20:1): δ = 1.65 (bs, 1D) ppm. **^{13}C NMR** (126 MHz, DMSO-*d$_6$*): δ = 158.0, 146.9, 138.7, 128.7, 128.5, 127.7, 126.1, 113.9, 55.4, 44.0 (t, *J* = 9.1 Hz), 22.2, 21.9 (t, *J* = 19.2 Hz) ppm.

HRMS (EI$^+$) m/z for $C_{15}H_{15}{}^2HO$ [M]$^+$: calc: 213.1258, found: 213.1261.

The analytical data are in accordance with the literature.[1a]

Cyclohexyl-2-deutero)benzene (10b)

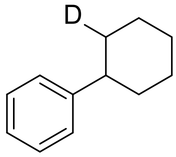

According to the general procedure 3 the title compound was prepared using 2,3,4,5-tetrahydro-1,1'-biphenyl (63.1 mg, 0.3 mmol, 1.0 equiv.) and 6,6-dideutero-3-hydro-3-phenyl-cyclohexa-1,4-dienyl)trimethyl-silane in DCE at 80 °C. The product was obtained as a colourless liquid (60.1 mg, 0.28 mmol, 94%).

R*f* = 0.74 (*n*-pentane).

^{1}H NMR (500 MHz, $CDCl_3$): δ = 7.30 (m, 2H), 7.24 – 7.16 (m, 3H), 2.51 (m, 1H), 1.94 – 1.81 (m, 3.6H), 1.77 (m, 1H), 1.49 – 1.35 (m, 3.6H), 1.32 – 1.20 (m, 1H) ppm. **^{2}H NMR** (77 MHz, $CHCl_3$:$CDCl_3$ = 20:1): δ = 1.90 (bs, 0.5D), 1.45 (bs, 0.5D) ppm. **^{13}C NMR** (126 MHz, $CDCl_3$): δ = 148.2, 128.4, 127.0, 125.9, 44.8, 44.7, 34.6 (d, *J* = 5.0 Hz), 34.3 (t, *J* = 9.9 Hz), 34.1 (t, *J* = 10.4 Hz), 27.1 (d, *J* = 13.3 Hz), 26.4 (d, *J* = 1.9 Hz) ppm.

HRMS (EI^+) m/z for $C_{12}H_{15}{}^2H$ [M^+]: calc.: 161.1309, found: 161.1305.

4-(1-(4-Fluorophenyl)ethyl-2-deutero)-1,1'-biphenyl (10c)

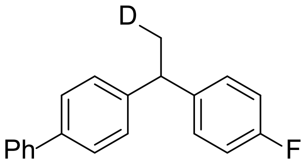

According to the general procedure 3 the title compound was prepared using 1-methoxy-4-(1-phenylvinyl)benzene (63.1 mg, 0.3 mmol, 1.0 equiv.) and 6,6-dideutero-3-hydro-3-phenylcyclohexa-1,4-dienyl)trimethylsilane in DCE at 80 °C. The product was obtained as a colourless solid (60.1 mg, 0.28 mmol, 94%).

R*f* = 0.76 (*n*-pentane:diethyl ether = 20:1).

^{1}H NMR (500 MHz, $CDCl_3$): δ = 7.61 – 7.57 (m, 2H), 7.57 – 7.51 (m, 2H), 7.46 – 7.41 (m, 2H), 7.36 – 7.31 (m, 1H), 7.31 – 7.27 (m, 2H), 7.26 – 7.19 (m, 2H), 7.04 – 6.97 (m, 2H), 4.19 (t, J = 7.1 Hz, 1H), 1.67 (t, J = 7.3 Hz, 2H) ppm. **^{2}H NMR** (77 MHz, $CDCl_3$): δ = 1.67 (bs, 1D) ppm. **^{13}C NMR** (126 MHz, $CDCl_3$): δ = 161.5 (d, *J* = 244.2 Hz), 145.4, 142.1 (d, *J* = 2.9 Hz), 141.1, 139.3, 129.2 (d, *J* = 7.8 Hz), 128.9, 128.1, 127.3, 127.3, 127.2, 115.3 (d, *J* = 21.1 Hz), 43.9 (t, *J* = 9.1 Hz), 22.2, 21.9 (t, *J* = 19.6 Hz) ppm. **^{19}F NMR** (470 MHz, $CDCl_3$): δ = -117.35 ppm.

HRMS (EI^+) m/z for $C_{20}H_{16}{}^2HF$ [M^+]: calc.: 277.1372, found: 277.1361.

(Propane-1,1-diyl-2-deutero)dibenzene (10d)

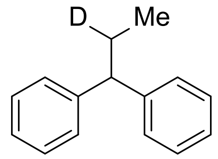

According to the general procedure 3 the title compound was prepared using 1-methoxy-4-(1-phenylvinyl)benzene (63.1 mg, 0.3 mmol, 1.0 equiv.) and 6,6-dideutero-3-hydro-3-phenyl-cyclohexa-1,4-dienyl)trimethylsilane in DCE at 80 °C. The product was obtained as a colourless liquid (60.1 mg, 0.28 mmol, 94%).

$\mathbf{R}_f$ = 0.32 (*n*-pentane).

^{1}H NMR (500 MHz, $CDCl_3$): δ = 7.30 – 7.26 (m, 4H), 7.26 – 7.22 (m, 4H), 7.20 – 7.15 (m, 2H), 3.80 (t, *J* = 7.5 Hz, 1H), 2.14 – 2.03 (m, 1.43 H), 0.91 (t, J = 7.4 Hz, 3H) ppm. **^{2}H NMR** (77 MHz, $CHCl_3$:$CDCl_3$ = 20:1): δ = 2.09 (bs, 1D) ppm. **^{13}C NMR** (126 MHz, $CDCl_3$): δ = 145.3, 128.5, 128.1, 126.2, 53.4, 28.4 (t, *J* = 19.5 Hz), 12.8 ppm. Additional peaks were observed at 53.4, 28.8 and 12.9 ppm for the CHCHDMe derivative.

HRMS (EI^+) m/z for $C_{15}H_{15}{}^2H$ [M^+]: calc.: 197.1309, found: 197.1310.

5 References

[1] (a) L. Li, G. Hilt, *Org. Lett.* **2020**, *22*, 1628–1632; (b) J. C. L. Walker, M. Oestreich, *Org. Lett.* **2018**, *20*, 6411–6414. (c) G. Zhang, R.-X. Bai, C.-H. Li, C.-G. Feng, G.-Q. Lin, *Tetrahedron* **2019**, *75*, 1658–1662. (d) J. Li, Q. Liu, H. Shen, R. Huang, X. Zhang, Y. Xiong, C. Chen, *RSC Adv.* **2015**, *5*, 85291–85295. (e) G. A. Molander, T. Fumagalli, *J. Org. Chem.* **2006**, *71*, 5743–5747. A. D. Strickland, R. A. Caldwell, *J. Phys. Chem.* **1993**, *97*, 13394–13402.

[2] G. Hilt, S. Lüers, *Synthesis* **2002**, 609–618.

[3] T. Aoyama, M. Hayakawa, S. Kubota, S. Ogawa, E. Nakajimaa, E. Mitsuyama, T. Iwabuchi, H. Kaneko, R. Obara, T. Takido, M. Kodomari, A. Ouchi, *Synthesis* **2015**, *47*, 2945–2956.

[4] I. Chatterjee, M. Oestreich, *Angew. Chem. Int. Ed.* **2015**, *54*, 1965–1968.

[5] S. J. Mahoney, T. Lou, G. Bondarenko, E. Fillion, *Org. Lett.* **2012**, *14*, 3473–3477.

[6] W. M. Dean, M. Šiaučiulis, T. E. Storr, W. Lewis, R. A. Stockman, *Angew. Chem. Int. Ed.* **2016**, *55*, 10013–10016.

[7] Q. Zhang, X. Wang, X. Wang, Q. Qian, H. Gong, *Synthesis* **2016**, *48*, 2829–2836.

[8] D. Liu, Y. Li, X. Qi, C. Liu, Y. Lan, A. Lei, *Org. Lett*. **2015**, *17*, 998–1001.

[9] D. A. Powell, G. Fu, *J. Am. Chem. Soc.* **2004**, *126*, 7788–7789.

[10] X. Li, Y. Feng, L. Lin, G. Zou, *J. Org. Chem.* **2012**, *77*, 10991–10995.

[11] N. G. Léonard P. J. Chirik, *ACS Catal*. **2018**, *8*, 342–348.

[12] T. Iwasaki, K. Yamashita, H. Kuniyasu, N. Kambe, *Org. Lett*. **2017**, *19*, 3691–3694.

6 Spectra

(*E*)-(4,4-Dideuteerobuta-1,3-dien-1-yl)benzene

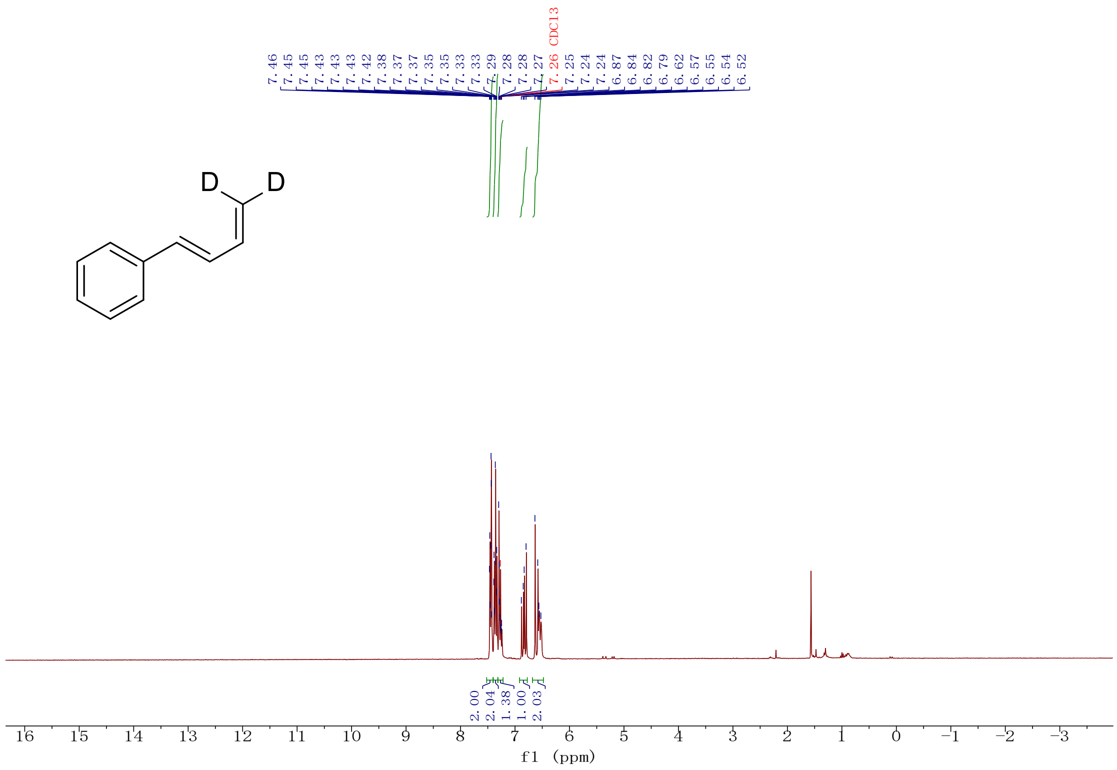

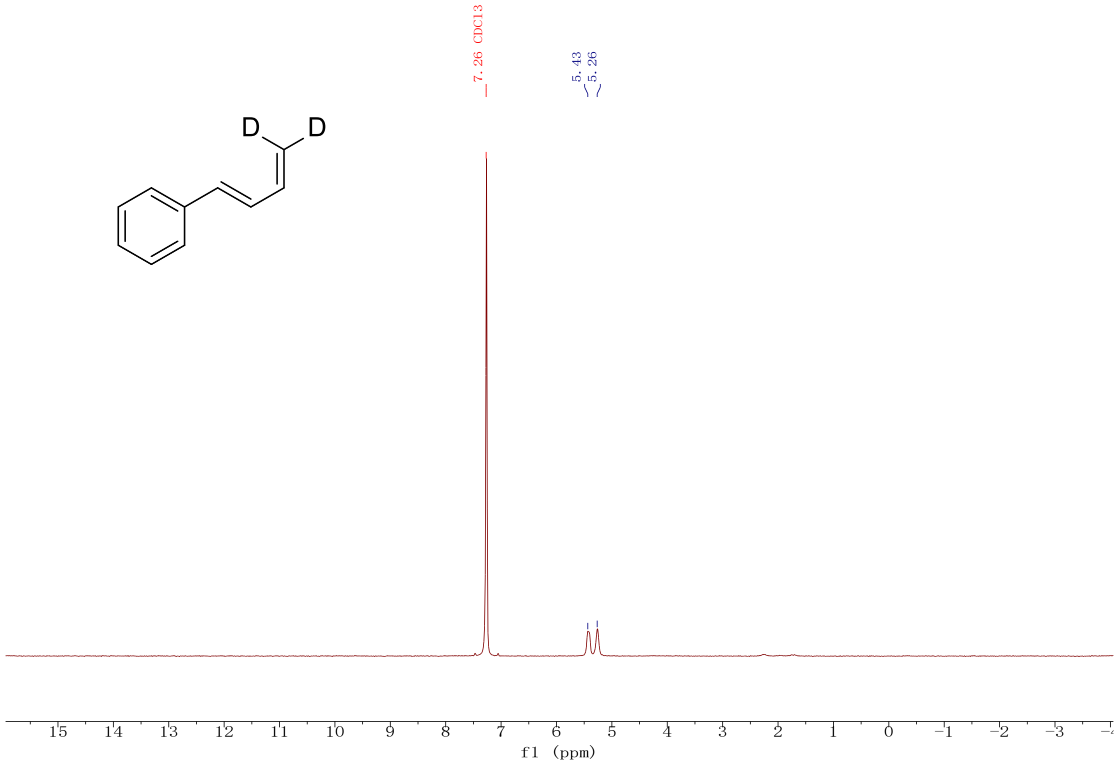

(1,4-Dihydro-[1,1'-biphenyl]-3-yl)trimethylsilane

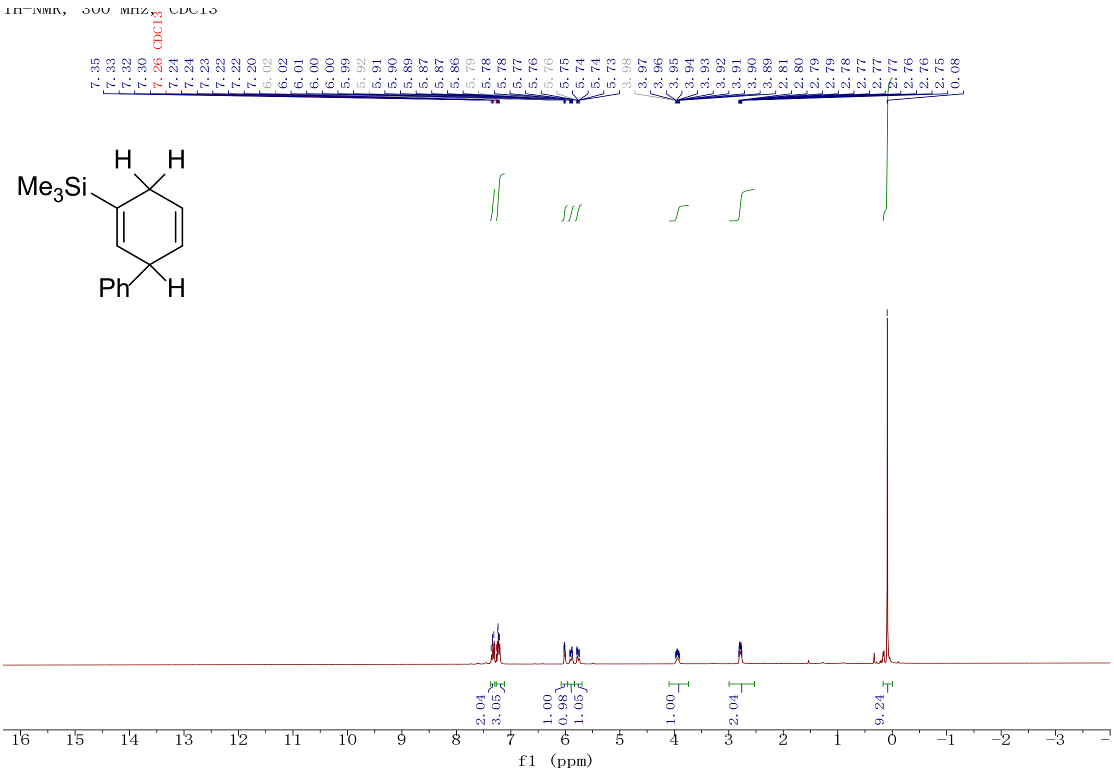

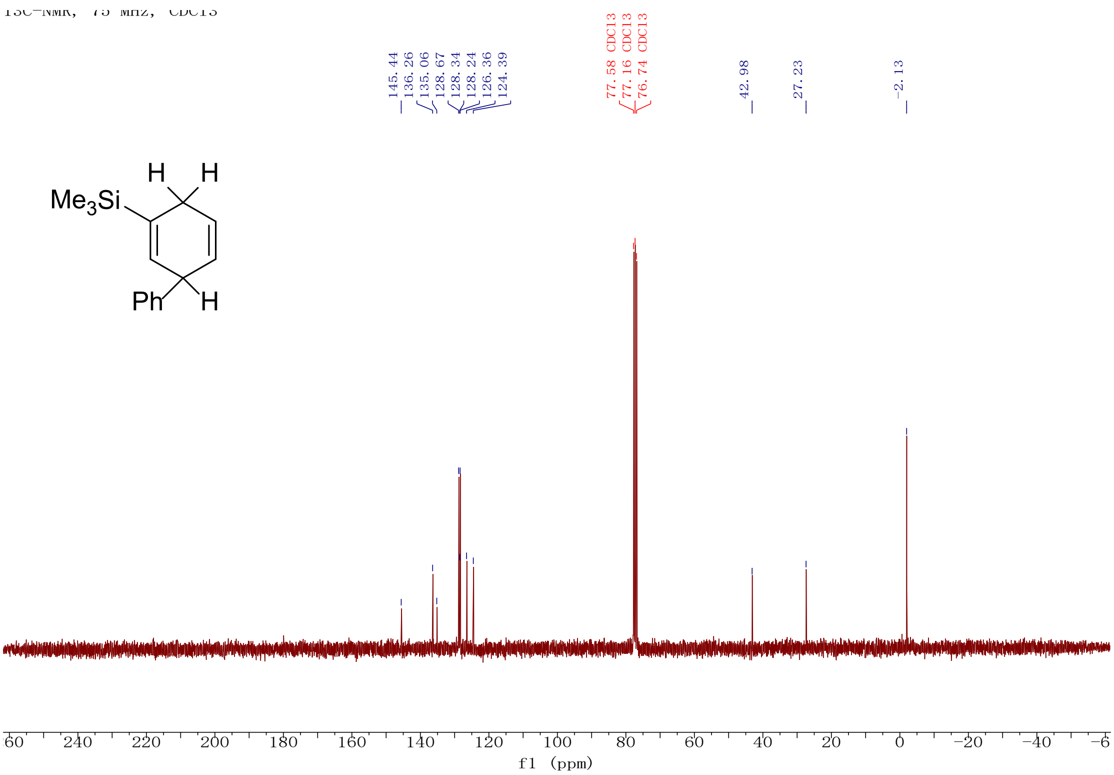

(3-Deutero-3-phenylcyclohexa-1,4-dienyl)trimethylsilane (3)

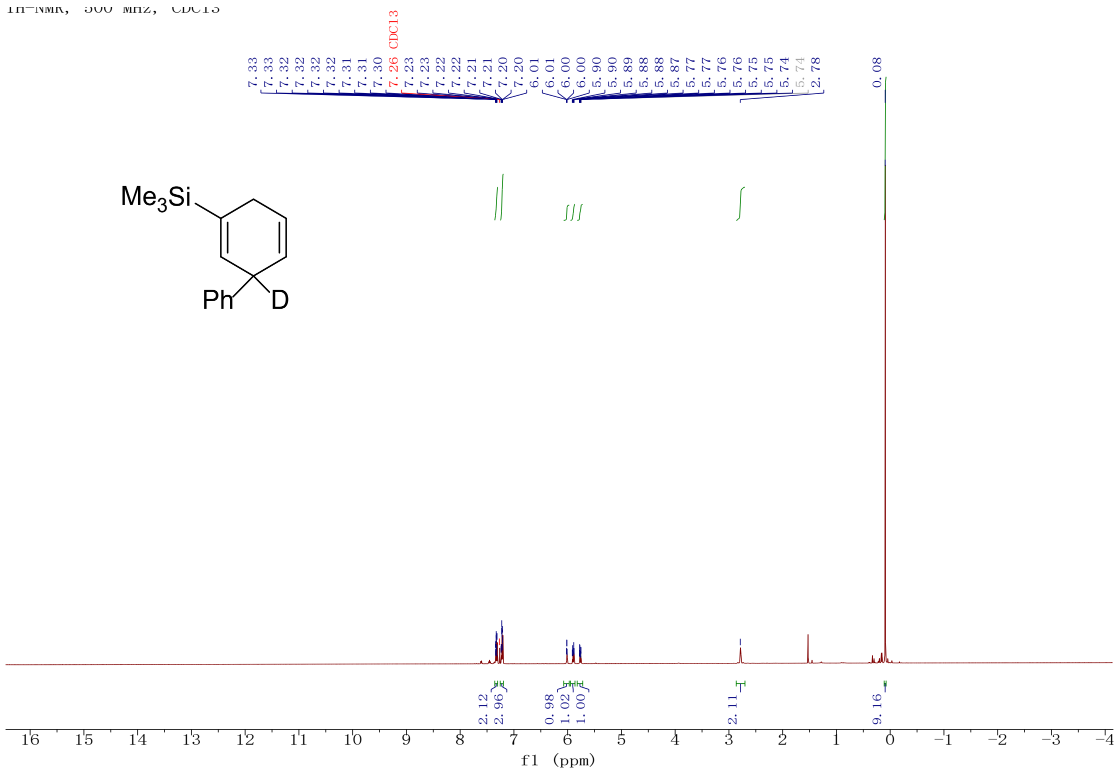

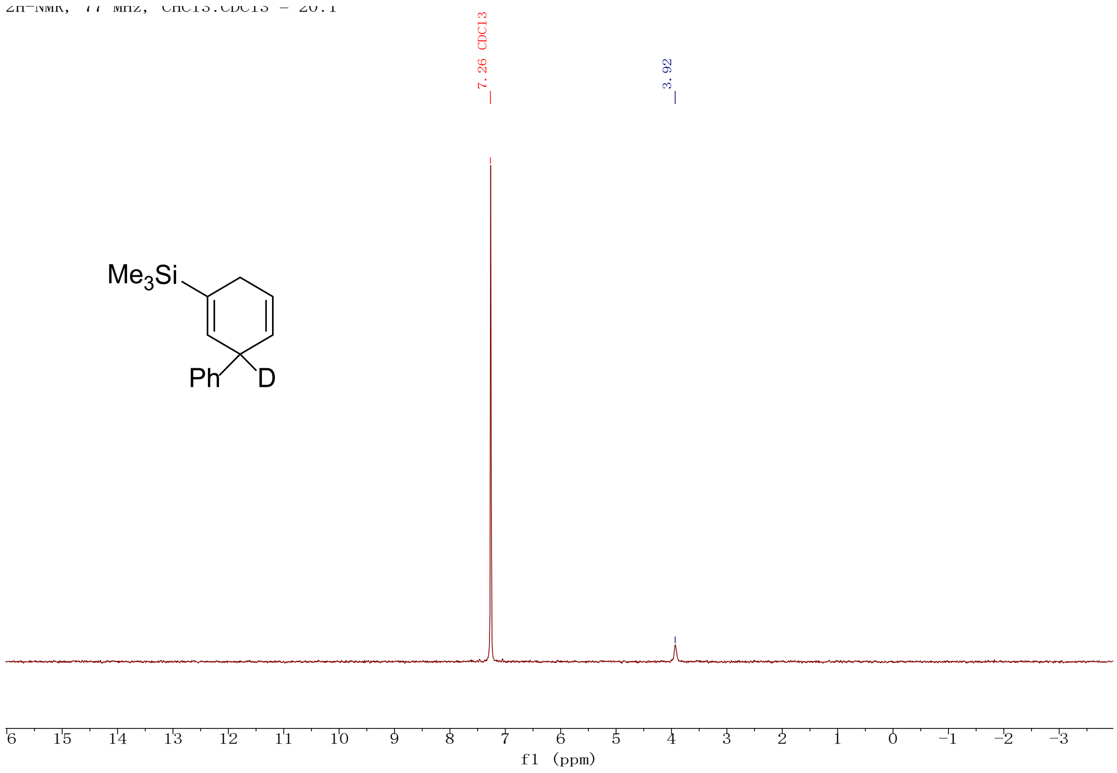

13C-NMR, 75 MHz, CDCl3

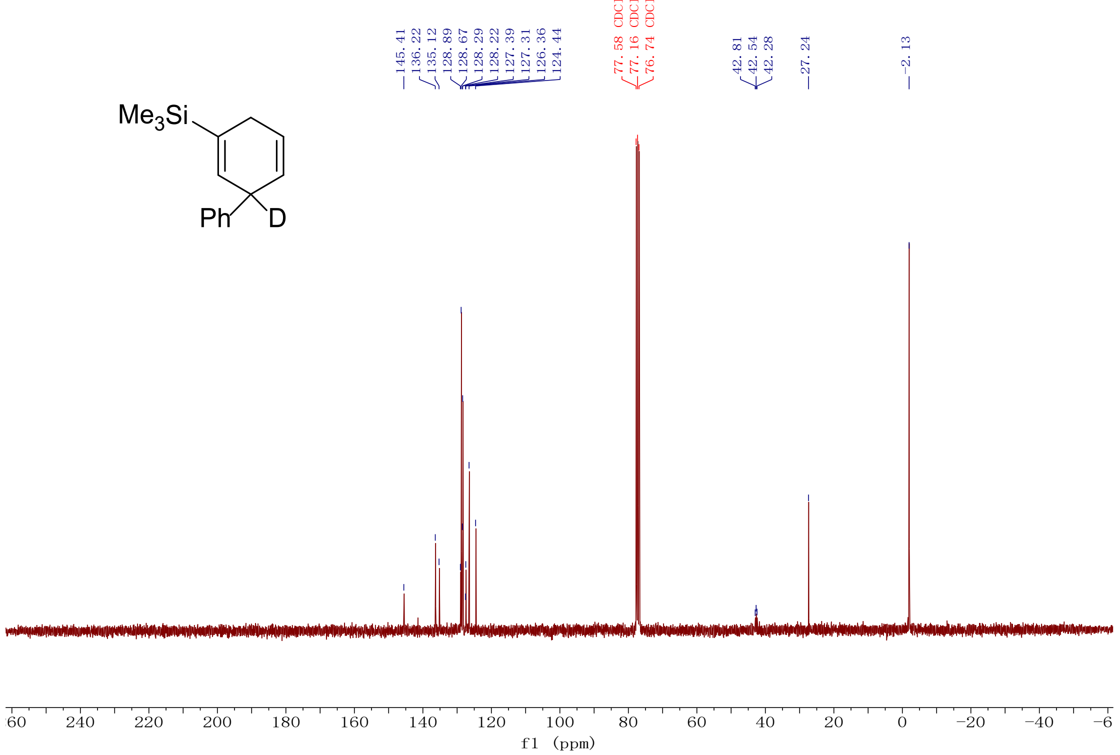

(6,6-Dideutero-3-hydro-3-phenylcyclohexa-1,4-dienyl)trimethylsilane (4)

1H-NMR, 500 MHz, CDCl3

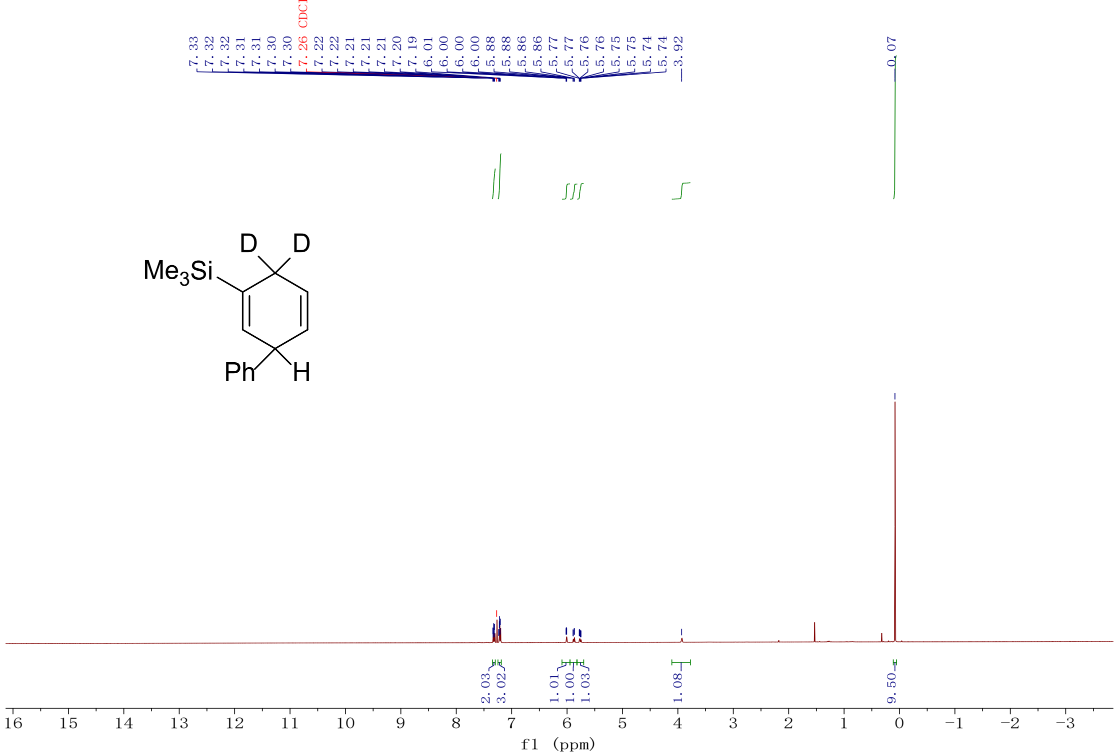

2H-NMR, 77 MHz, CHCl3:CDCl3 = 20:1

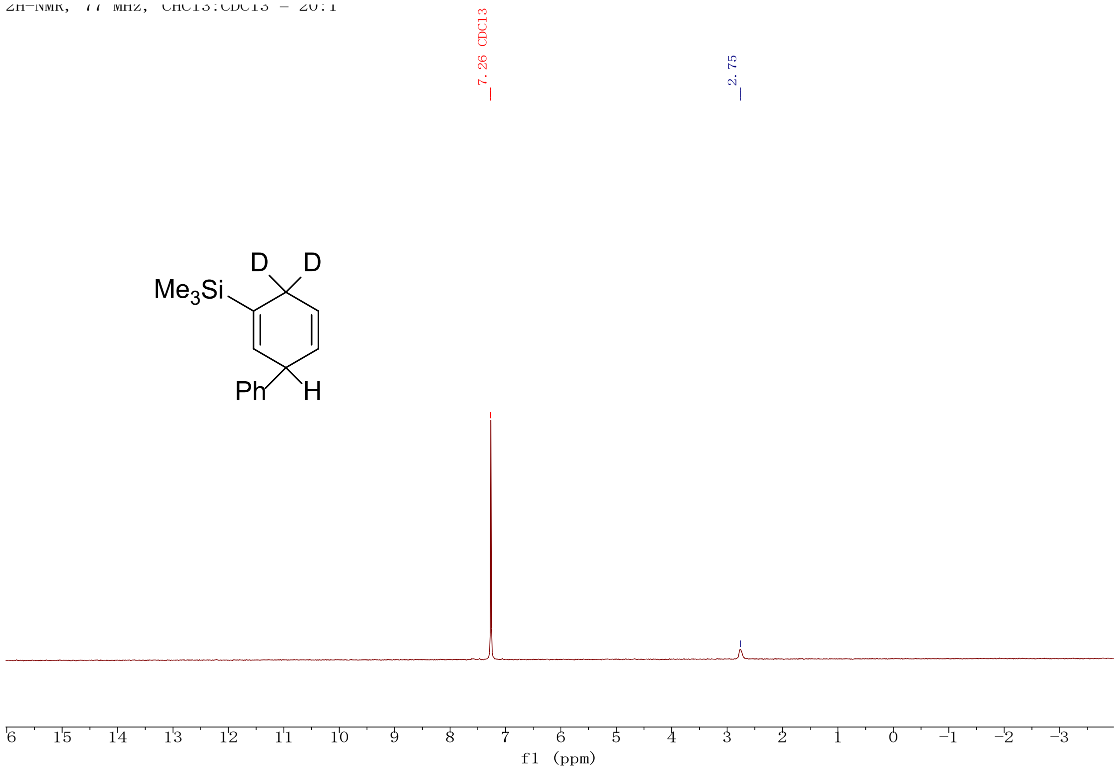

13C-NMR, 126 MHz, CDCl3

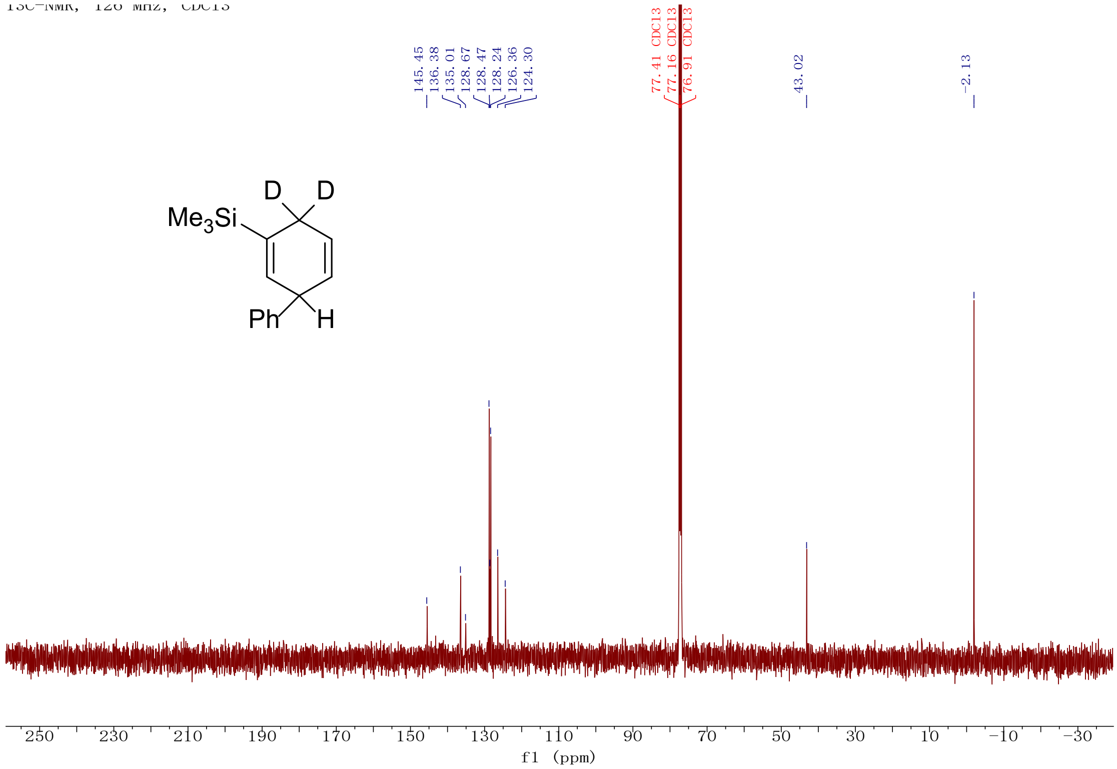

[1,1'-Biphenyl]-4-yl(4-fluorophenyl)methanone

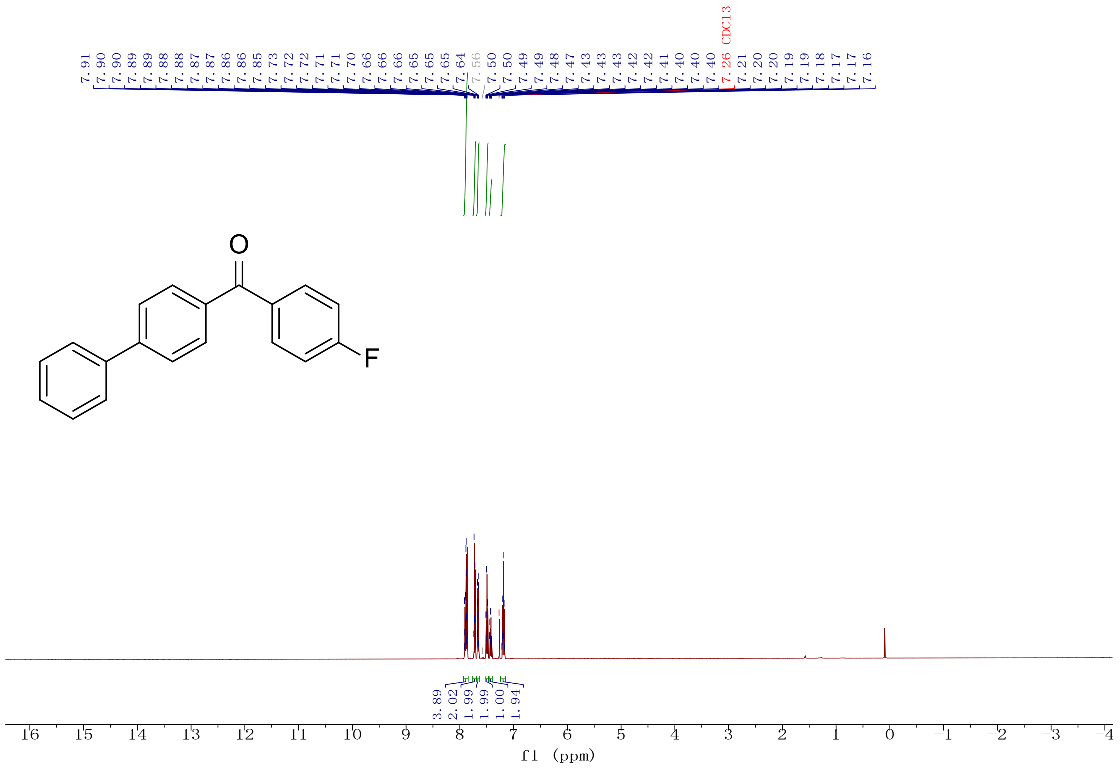

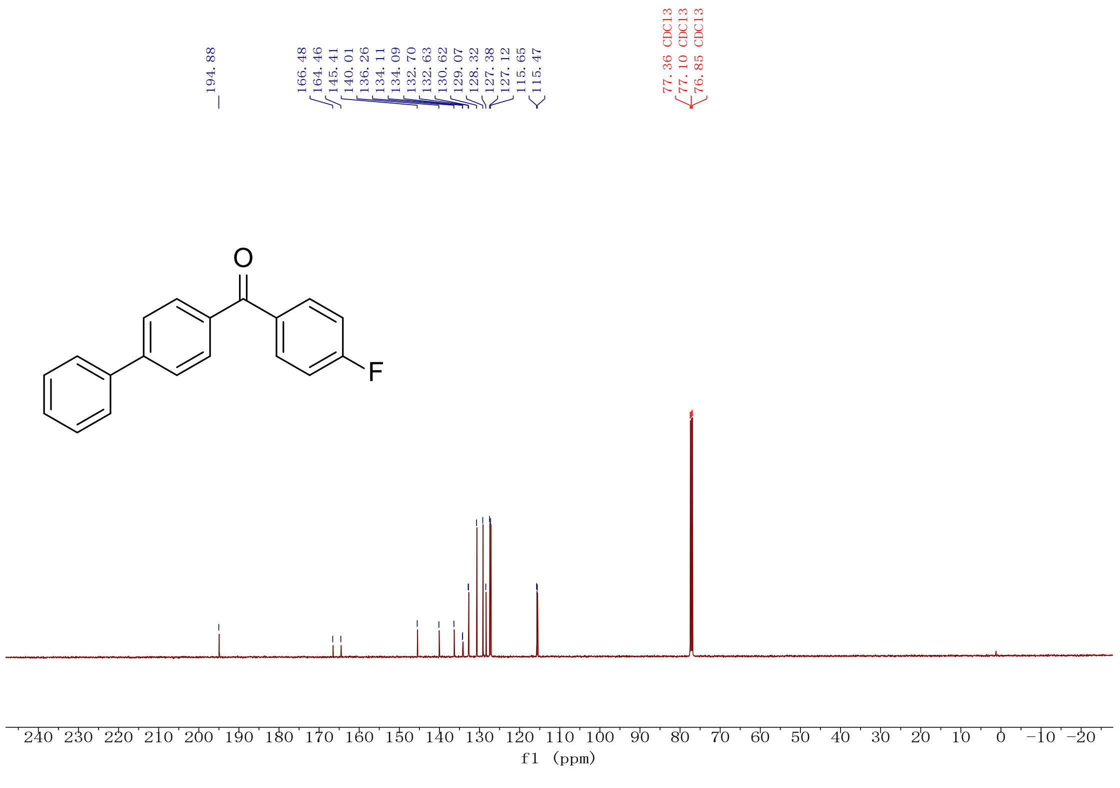

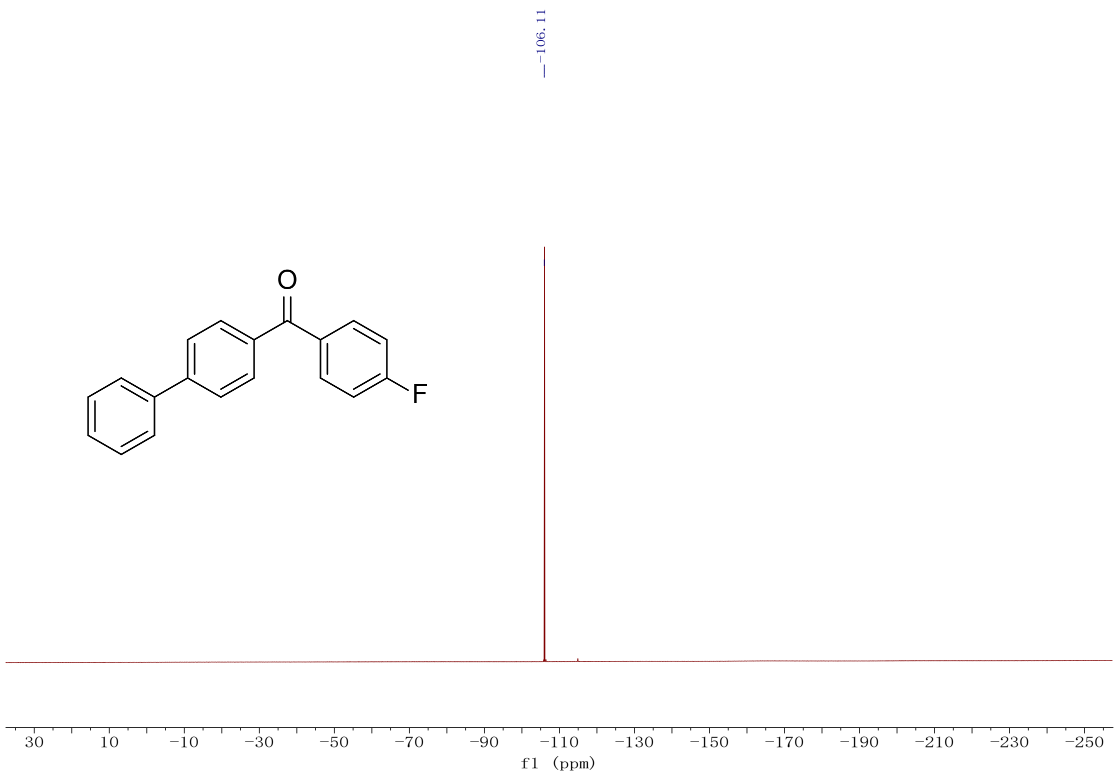

4-(1-(4-Fluorophenyl)vinyl)-1,1'-biphenyl (7j)

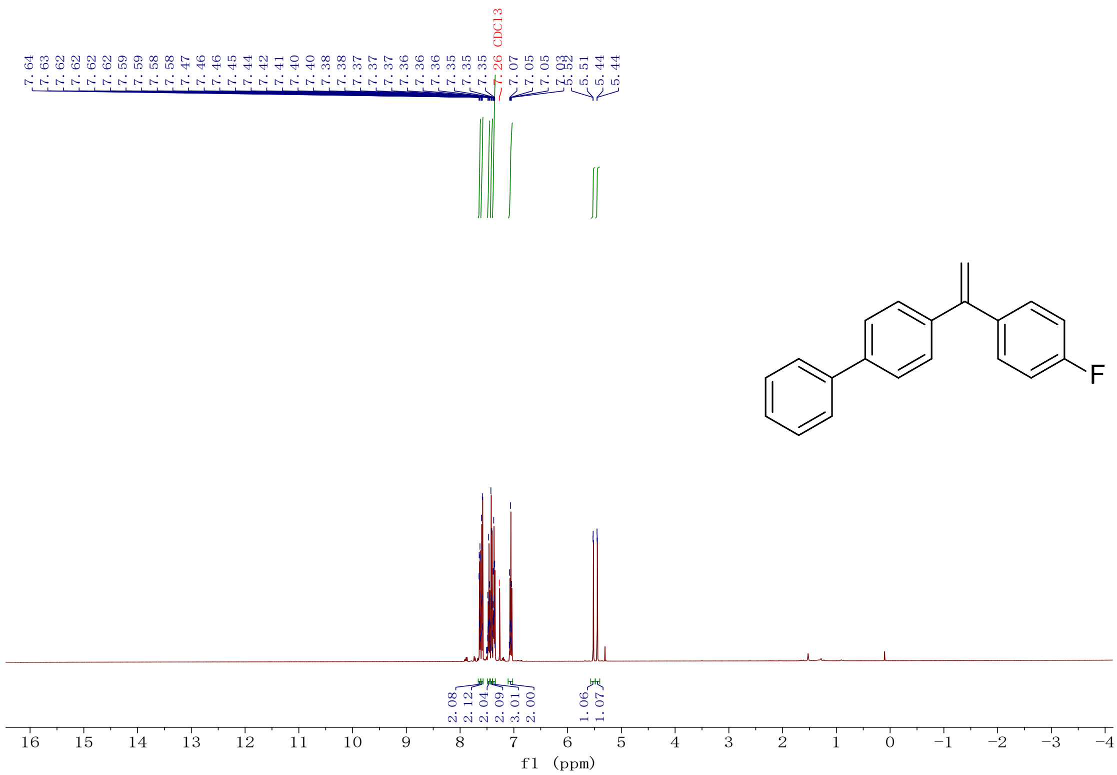

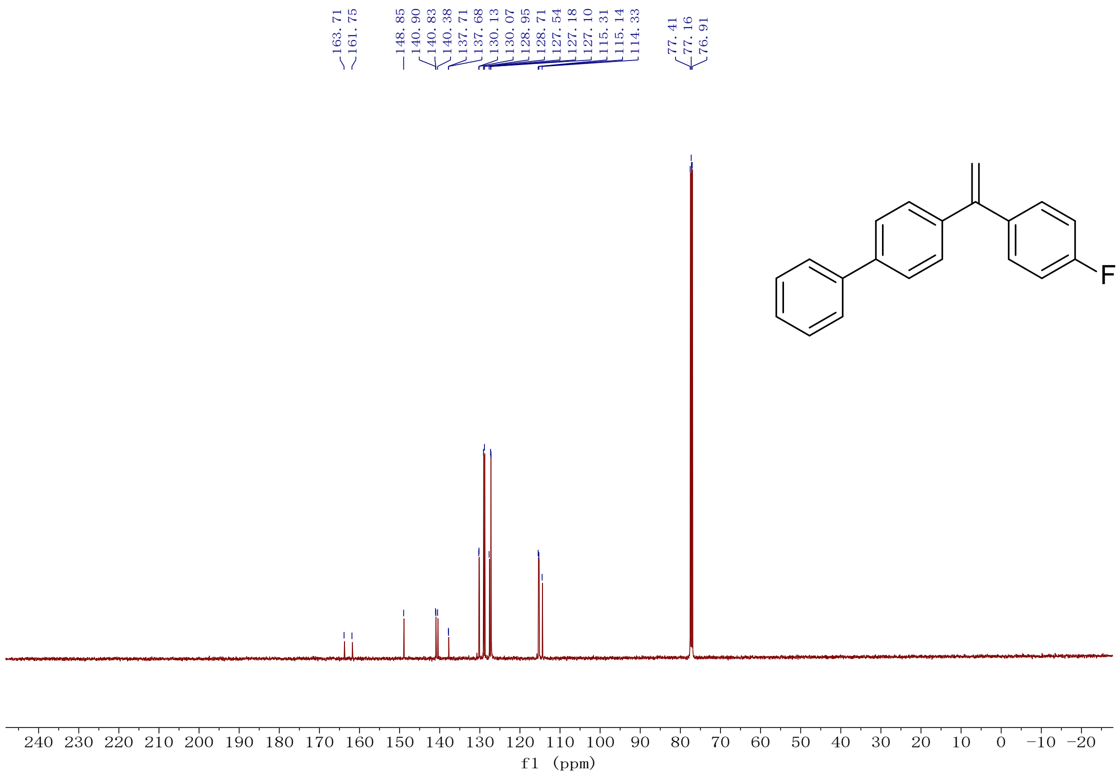
163.71
161.75
148.85
140.90
140.83
140.38
137.71
137.68
130.13
130.07
128.95
128.71
127.54
127.18
127.10
115.31
115.14
114.33
77.41
77.16
76.91
F
240 230 220 210 200 190 180 170 160 150 140 130 120 110 100 90 80 70 60 50 40 30 20 10 0 −10 −20
f1 (ppm)

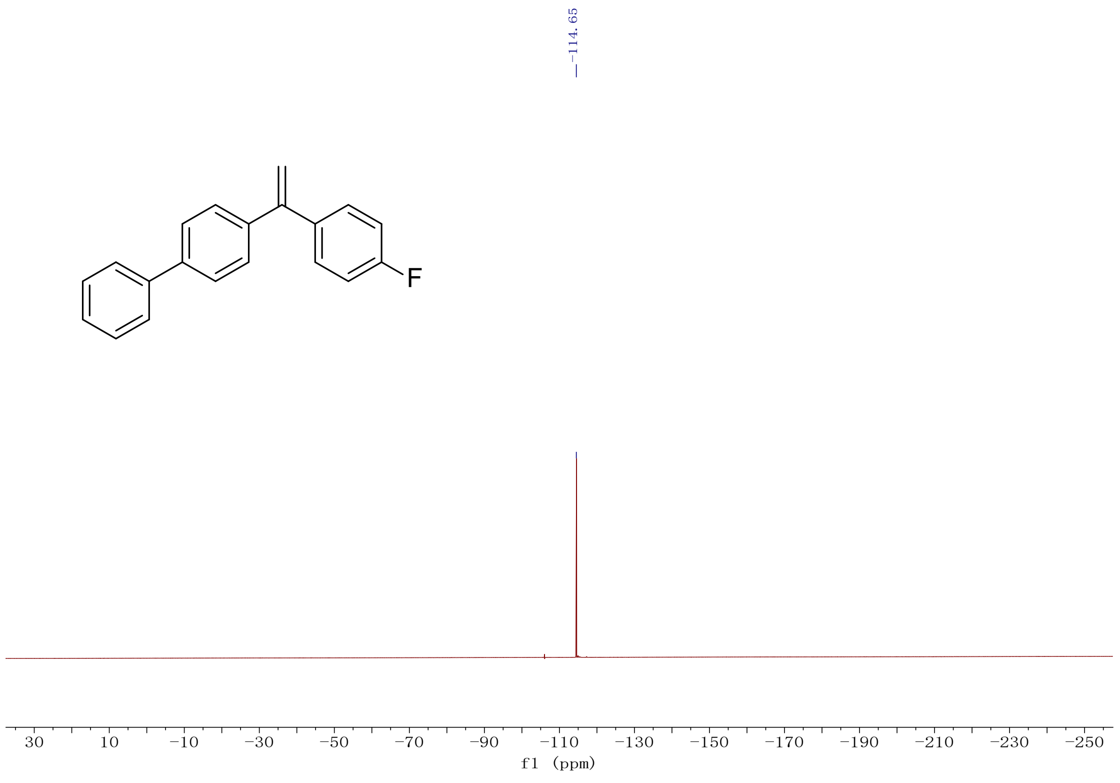
−114.65
F
30 10 −10 −30 −50 −70 −90 −110 −130 −150 −170 −190 −210 −230 −250
f1 (ppm)

Ethane-1,1-diyldibenzene (6)

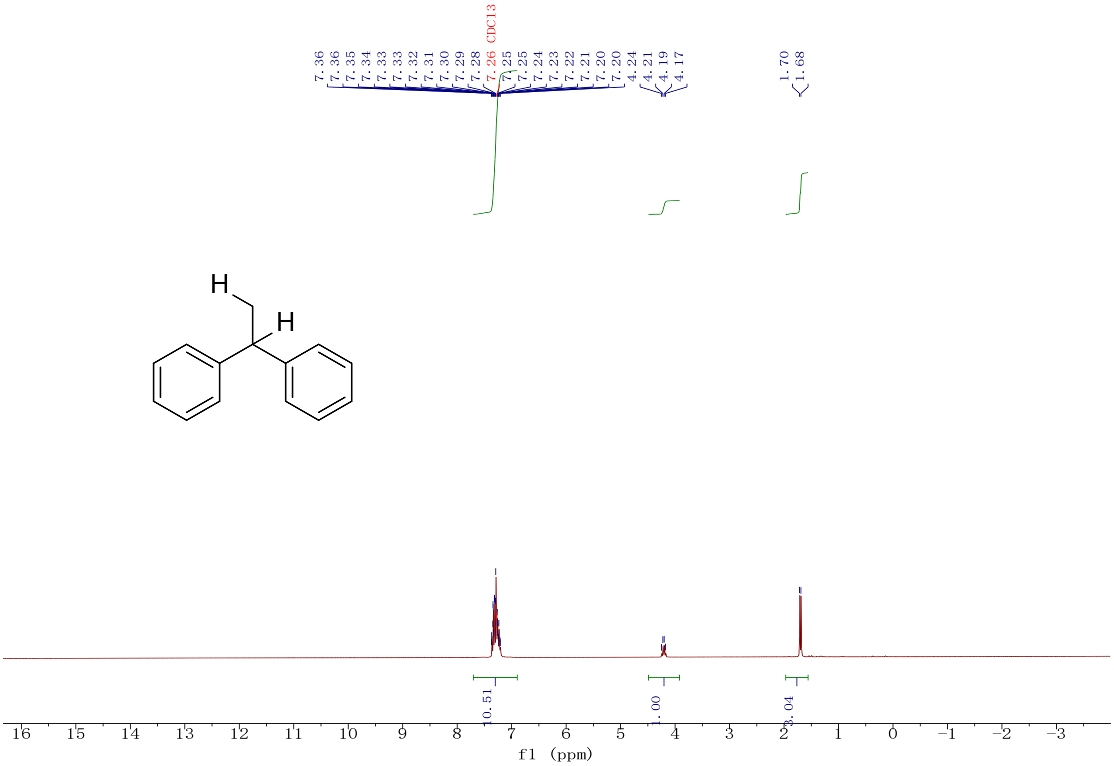

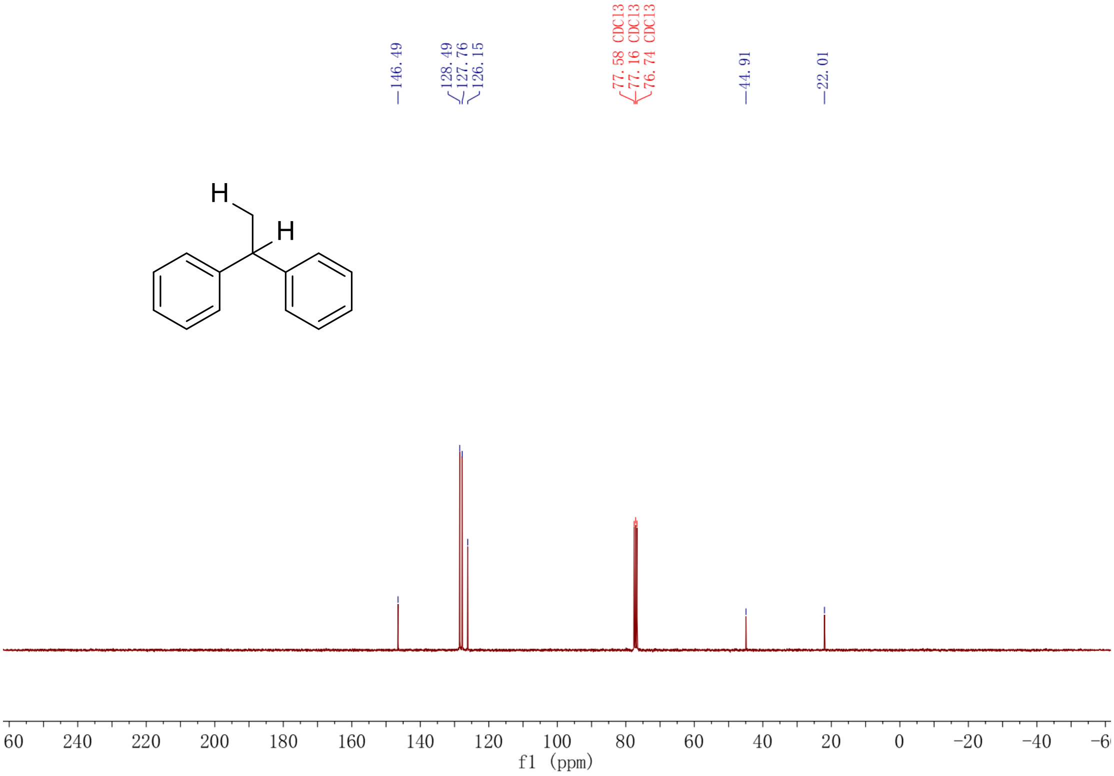

1-Methoxy-4-(1-phenylethyl)benzene (8a)

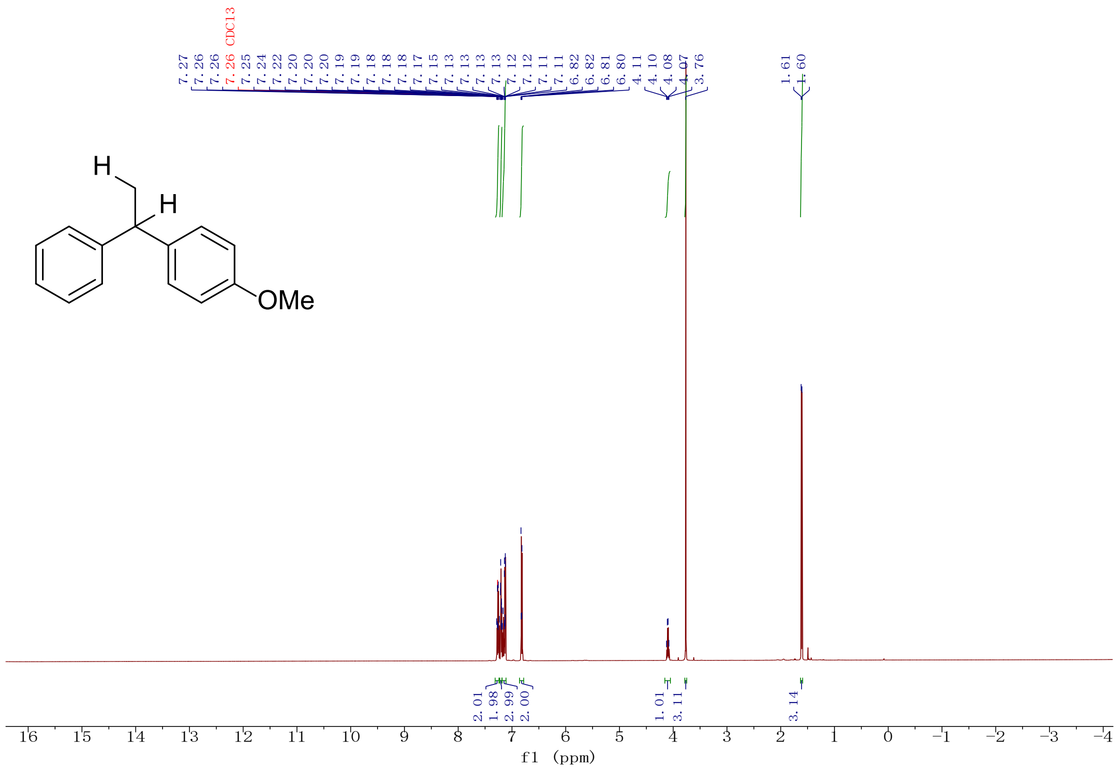

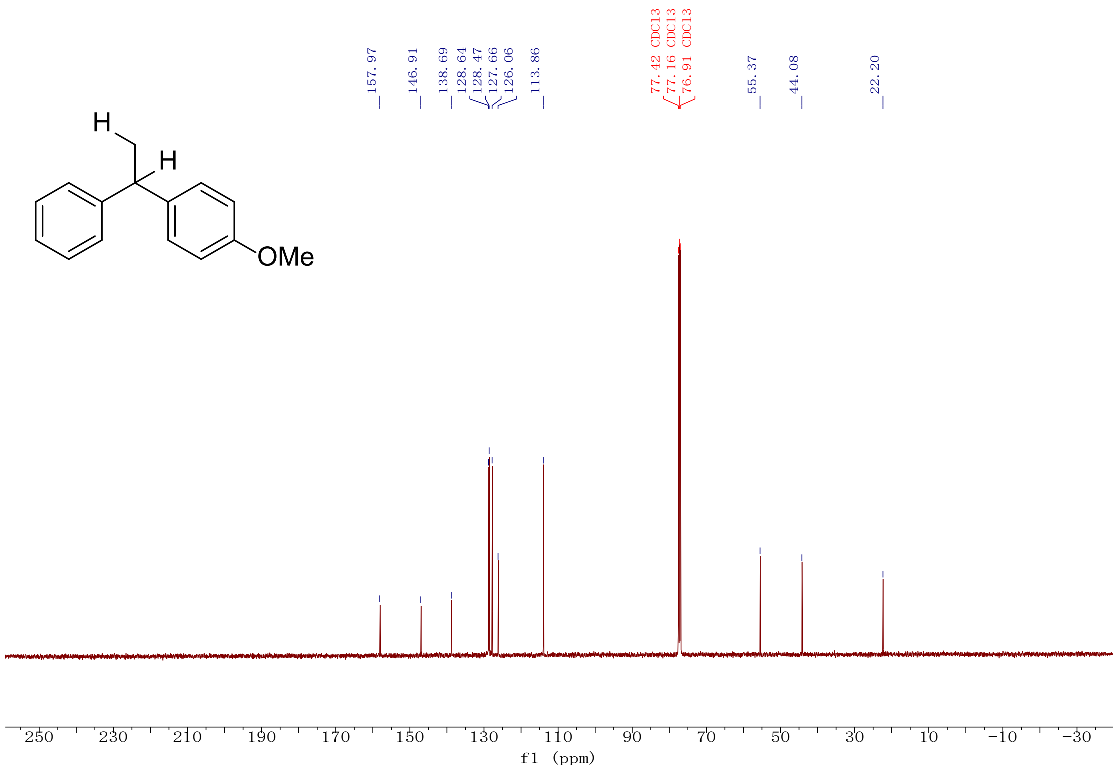

1-Methoxy-2-(1-phenylethyl)benzene (8b)

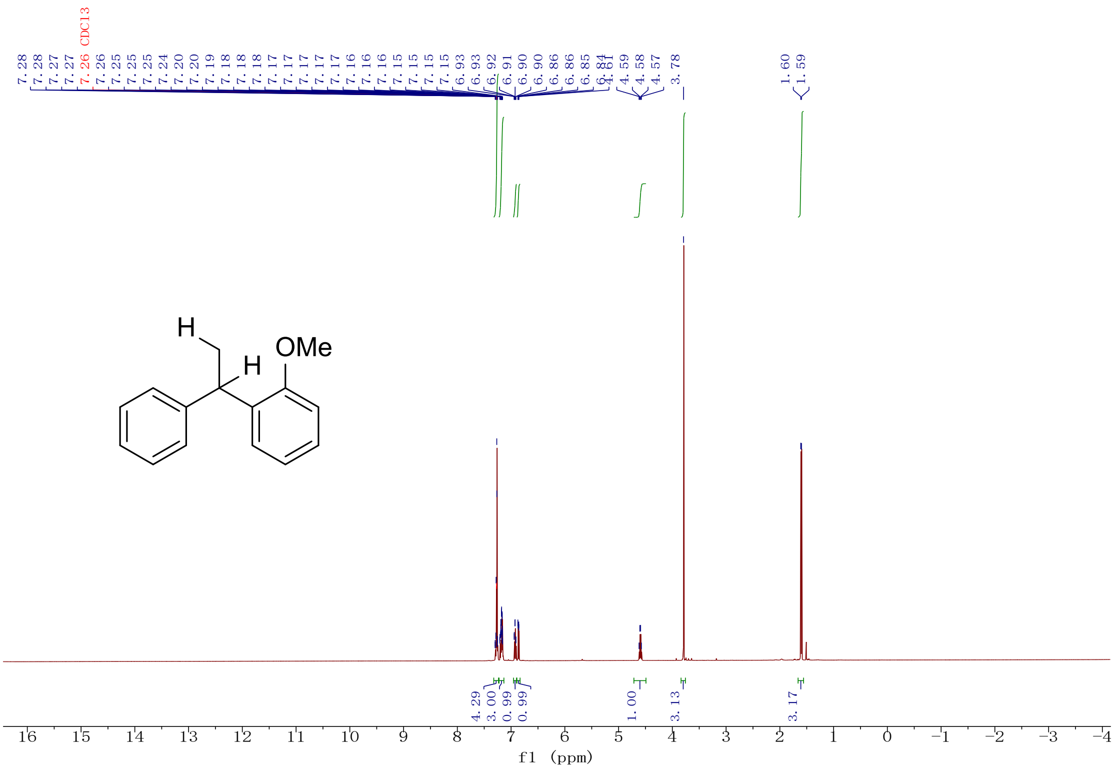

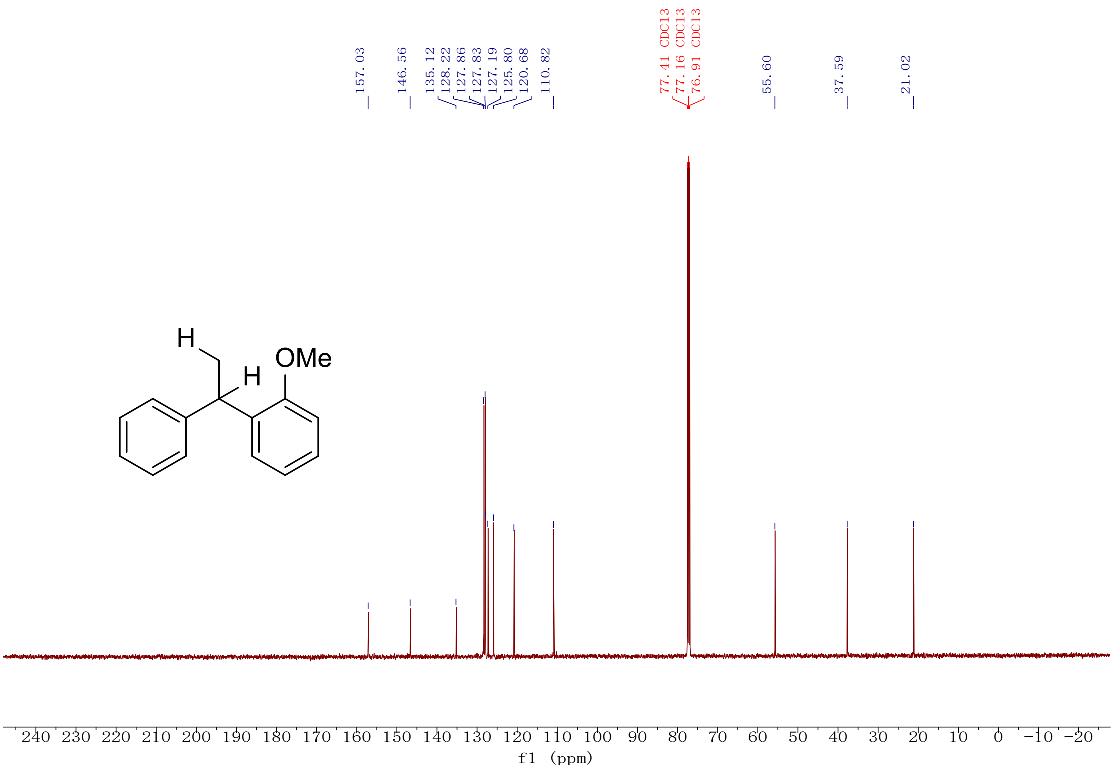

1-Methoxy-3-(1-phenylethyl)benzene (8c)

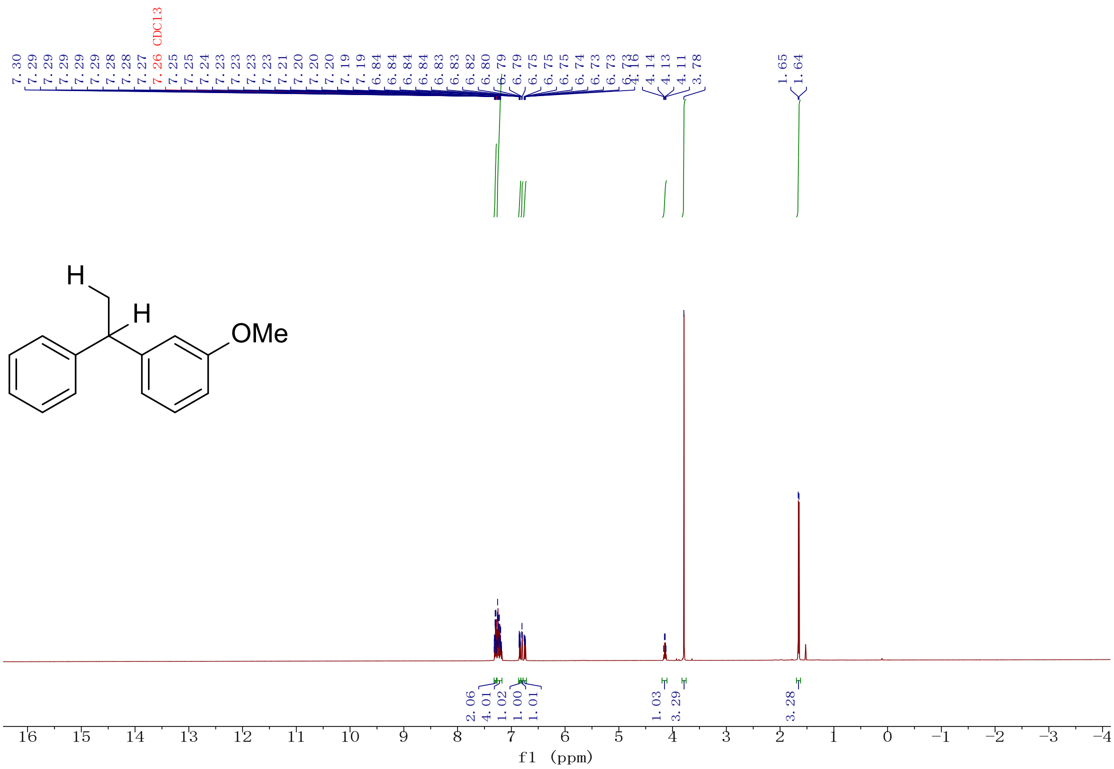

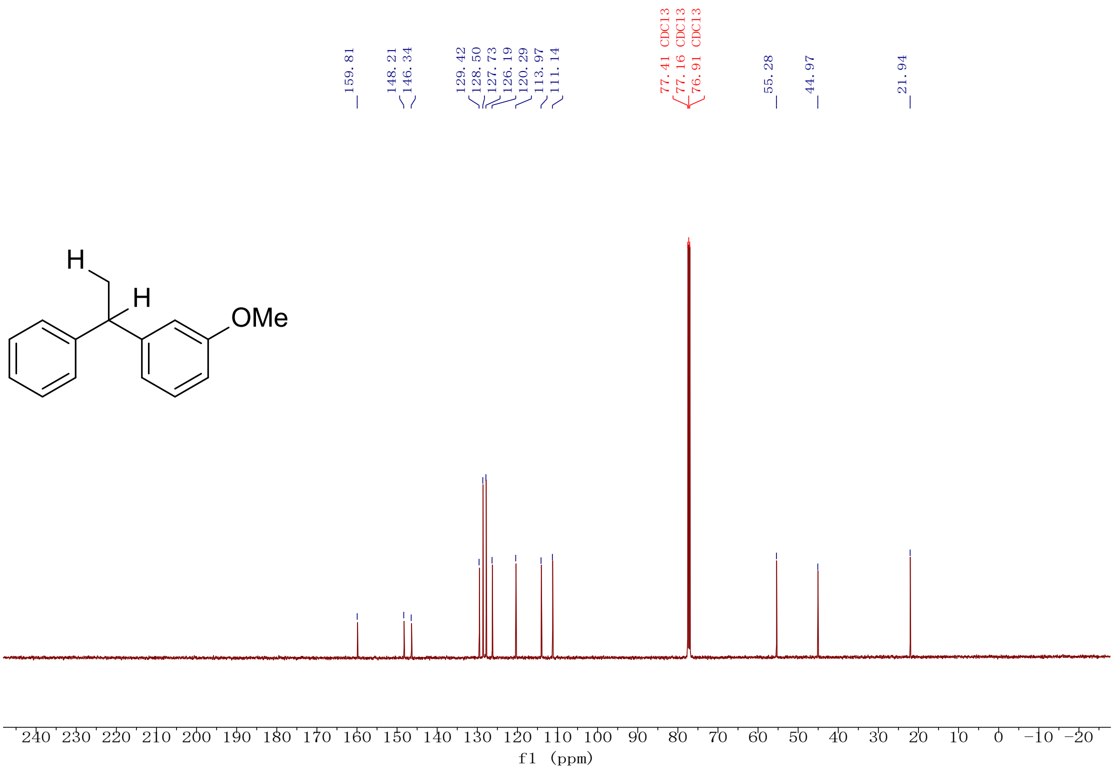

1,2-Dimethoxy-4-(1-phenylethyl)benzene (8d)

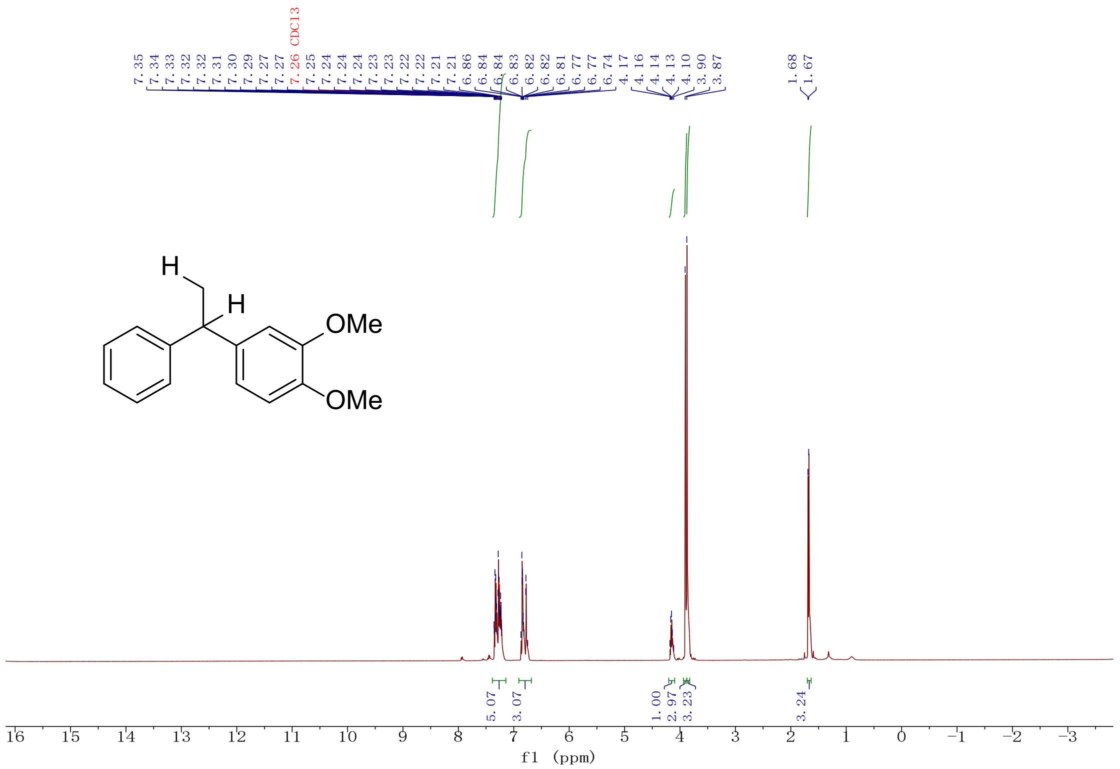

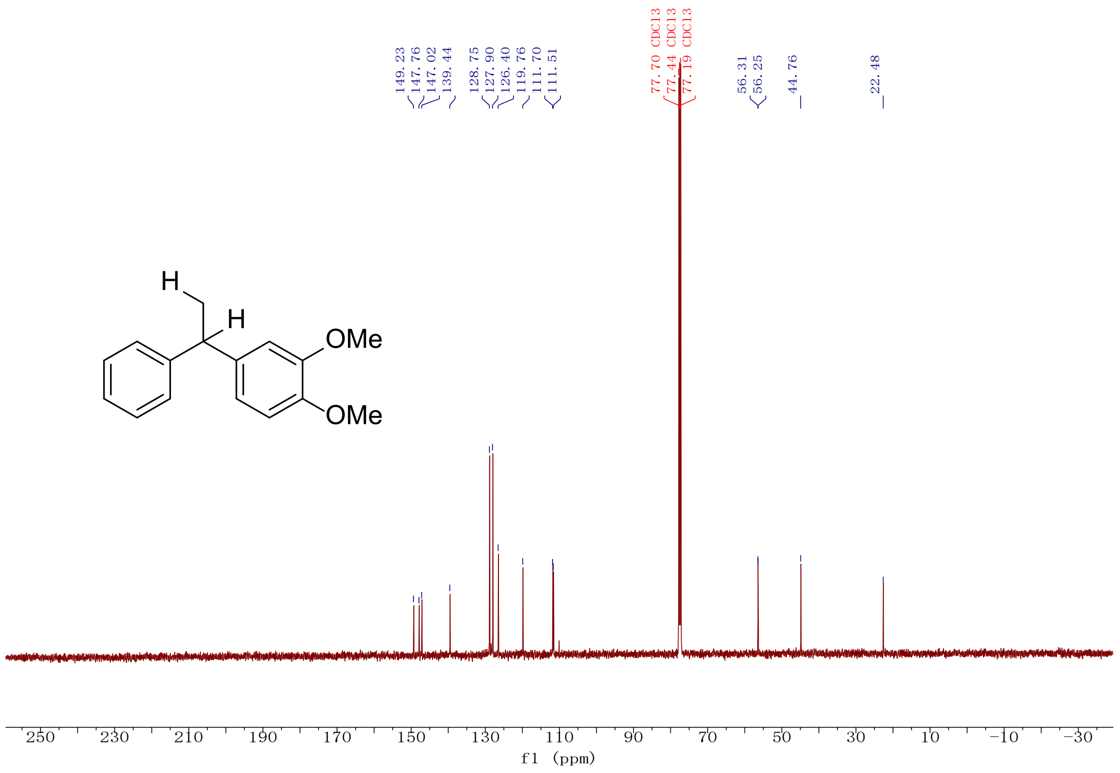

4,4'-(ethane-1,1-diyl)bis(methoxybenzene) (8e)

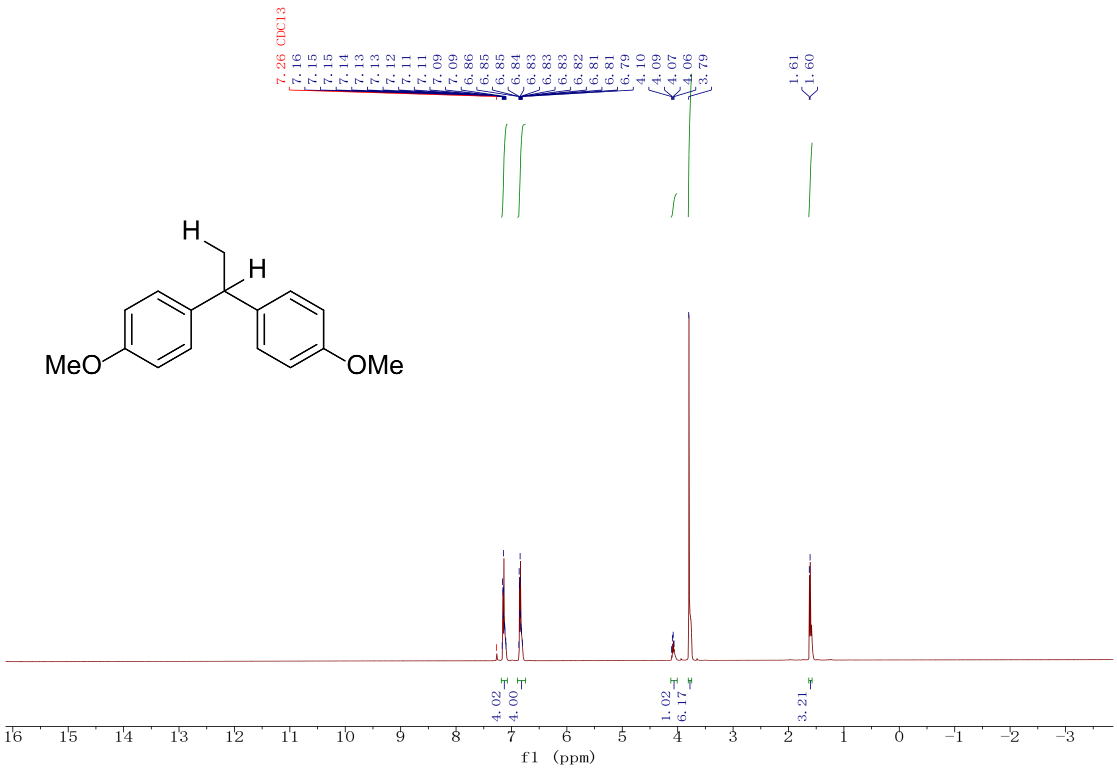

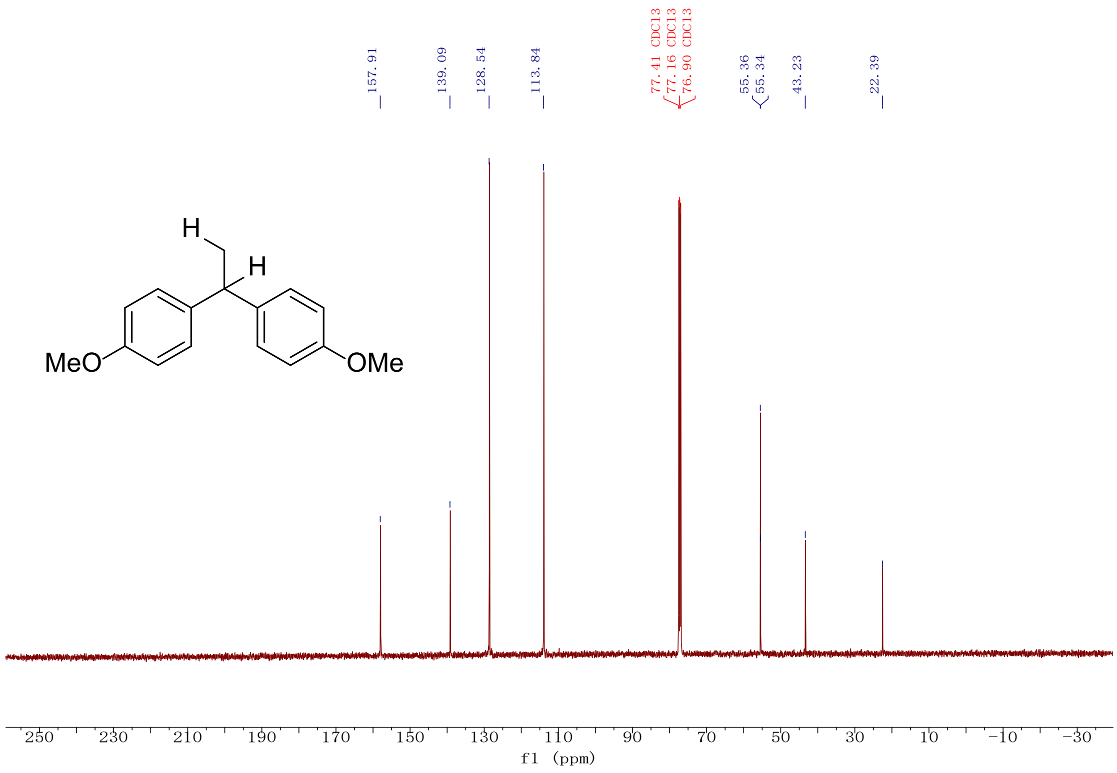

4,4'-(Ethane-1,1-diyl)bis(fluorobenzene) (8f)

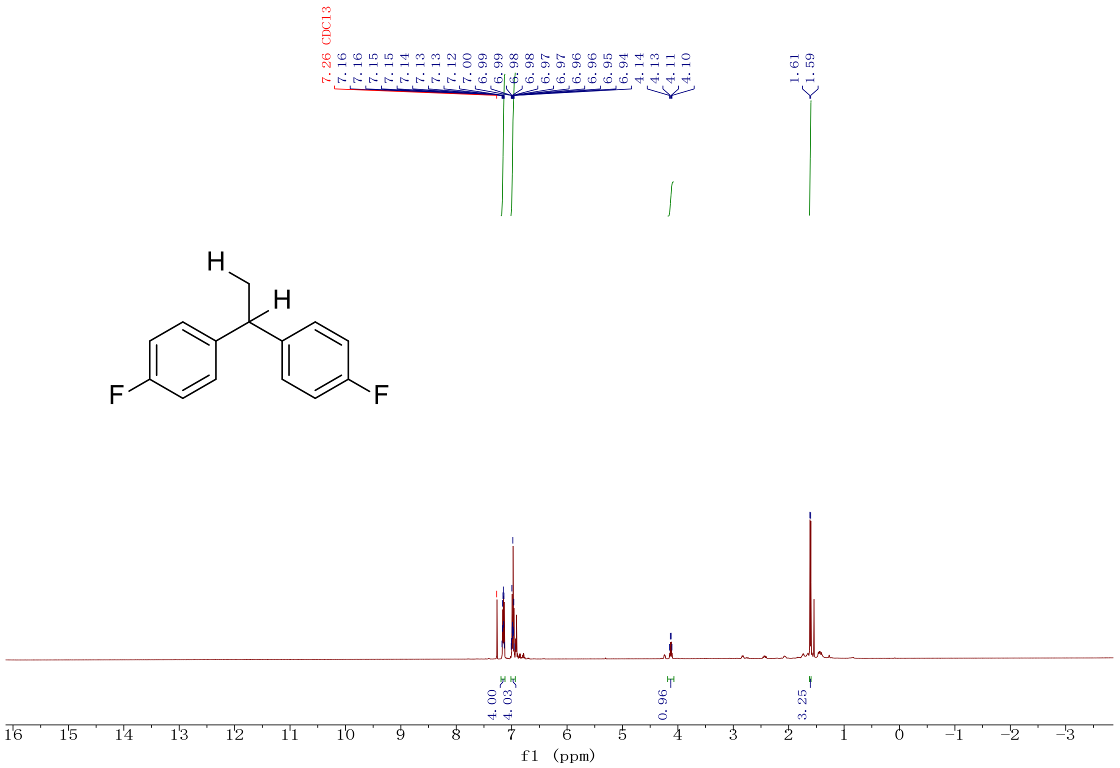

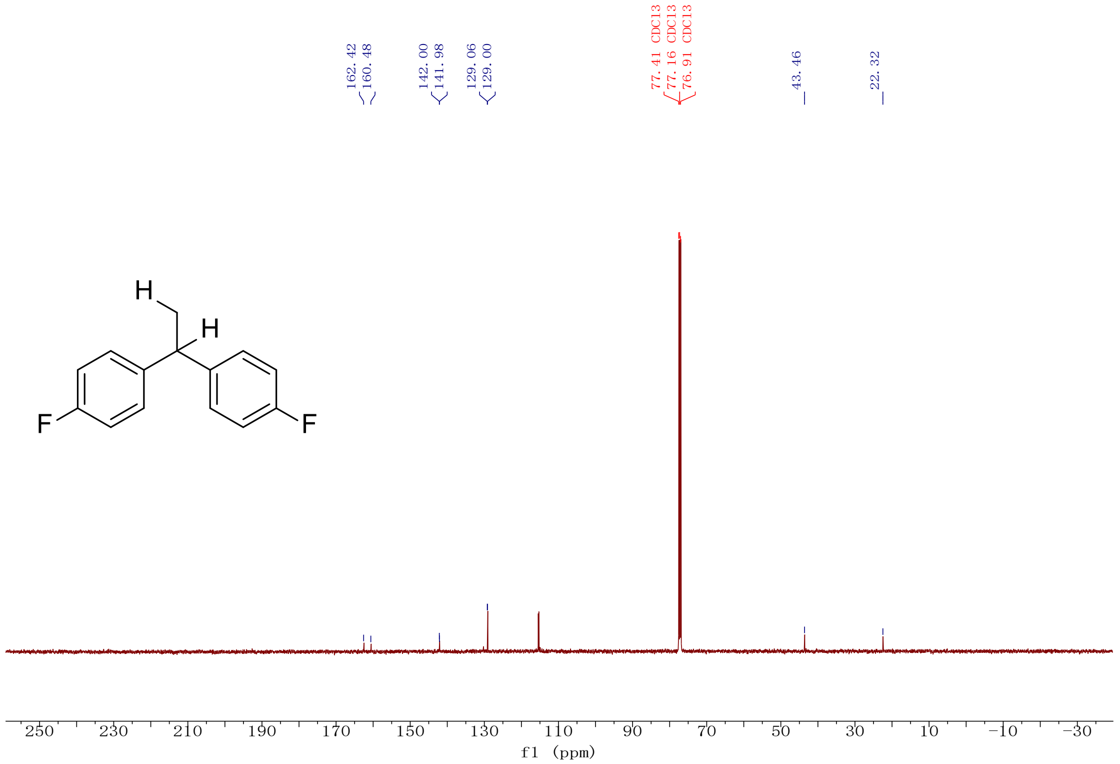

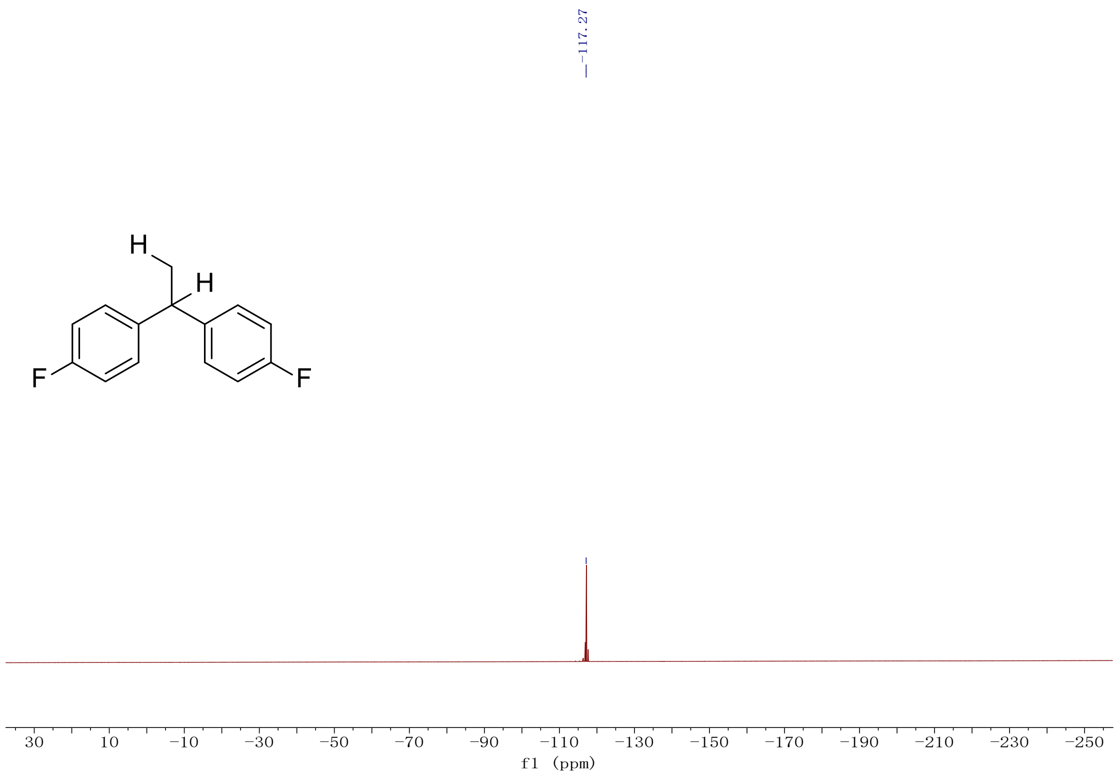

4,4'-(Ethane-1,1-diyl)bis(chlorobenzene) (8g)

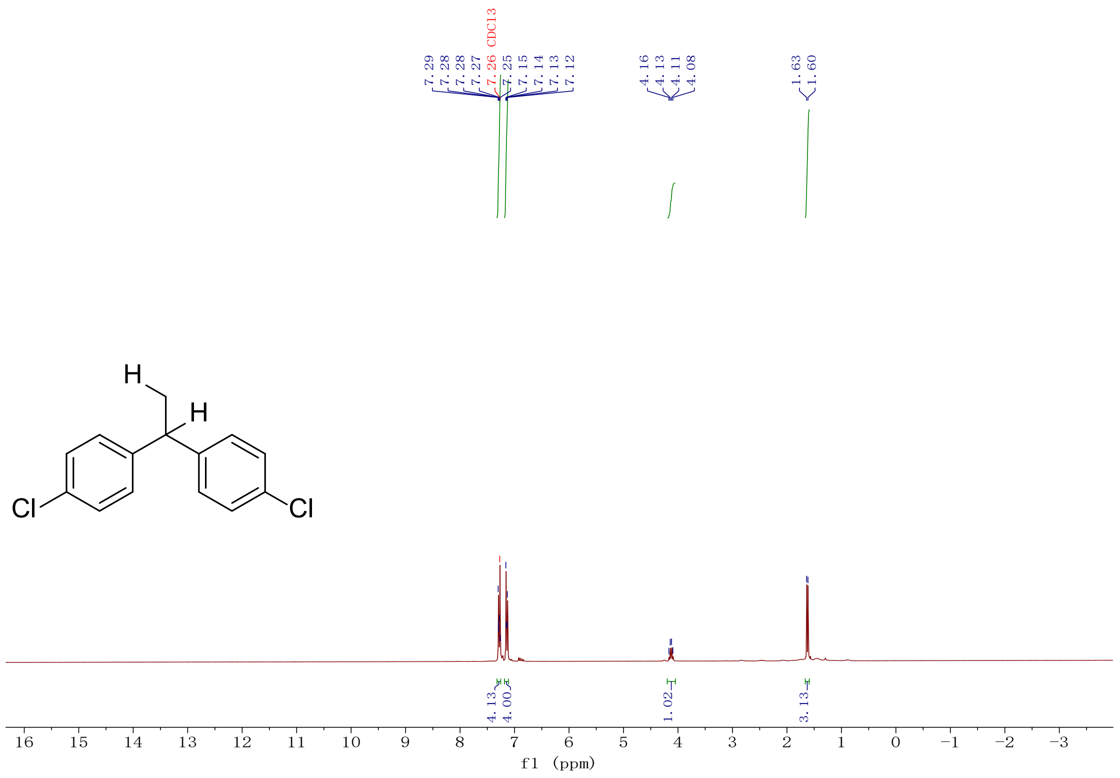

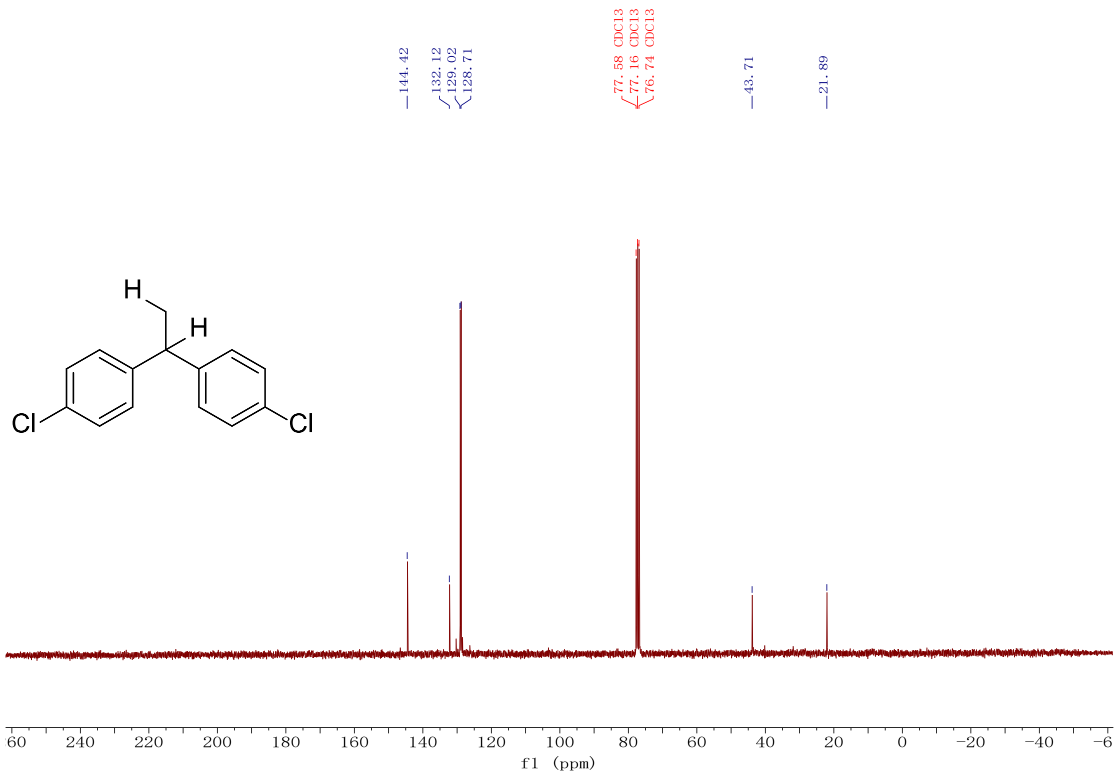

1-Bromo-4-(1-phenylethyl)benzene (8h)

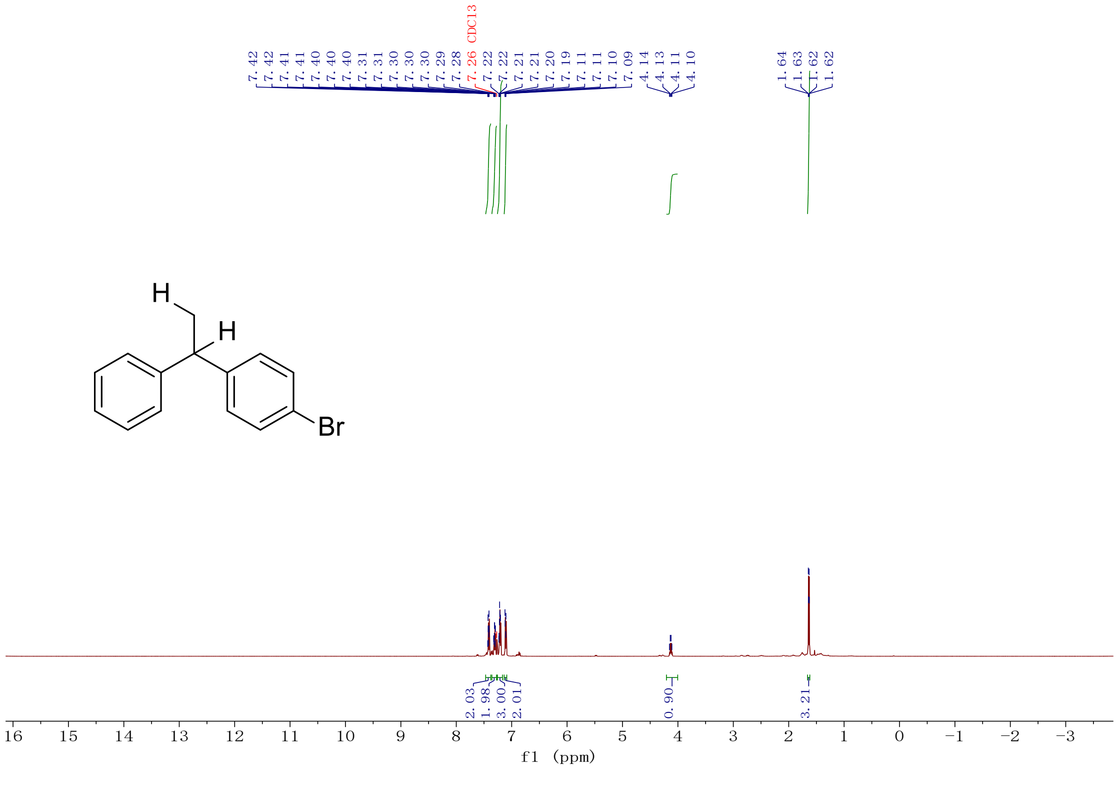

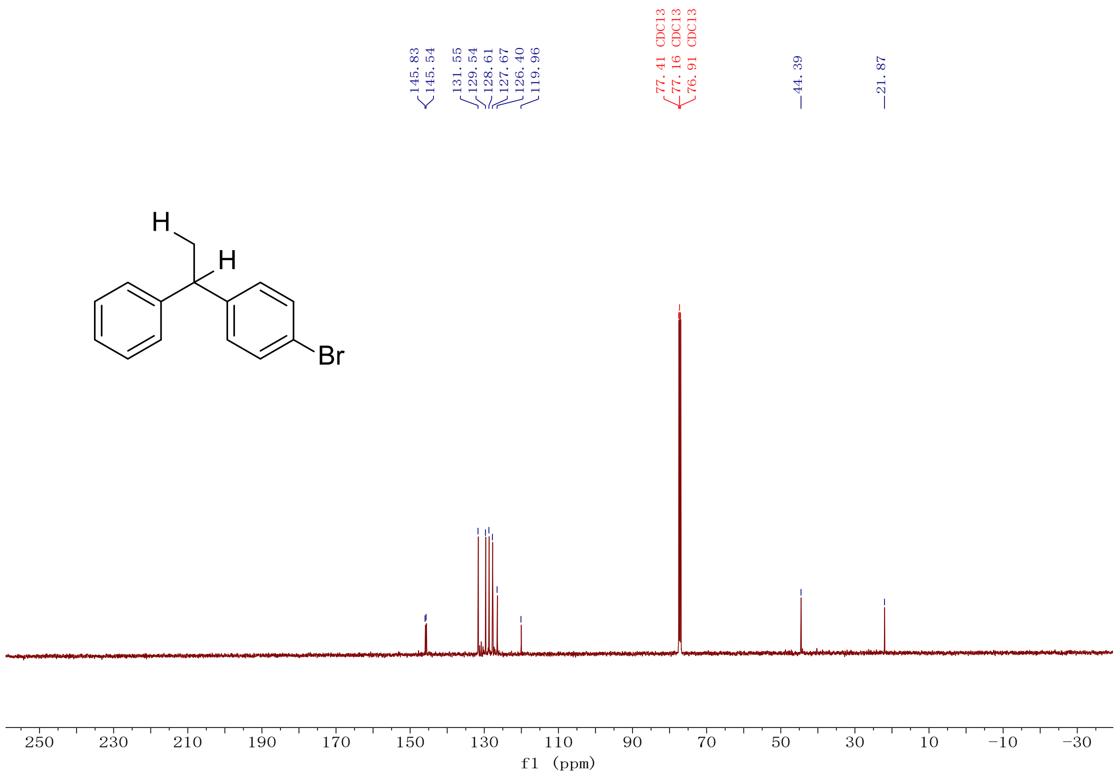

1-(1-Phenylethyl)-4-(trifluoromethyl)benzene (8i)

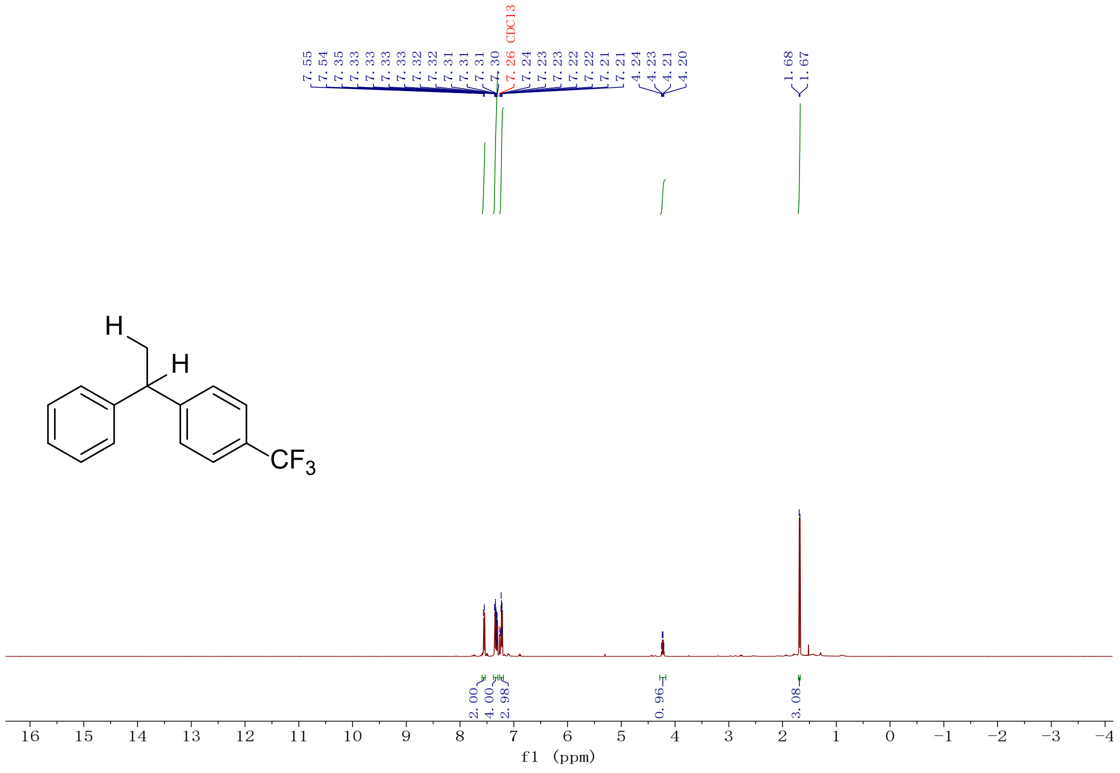

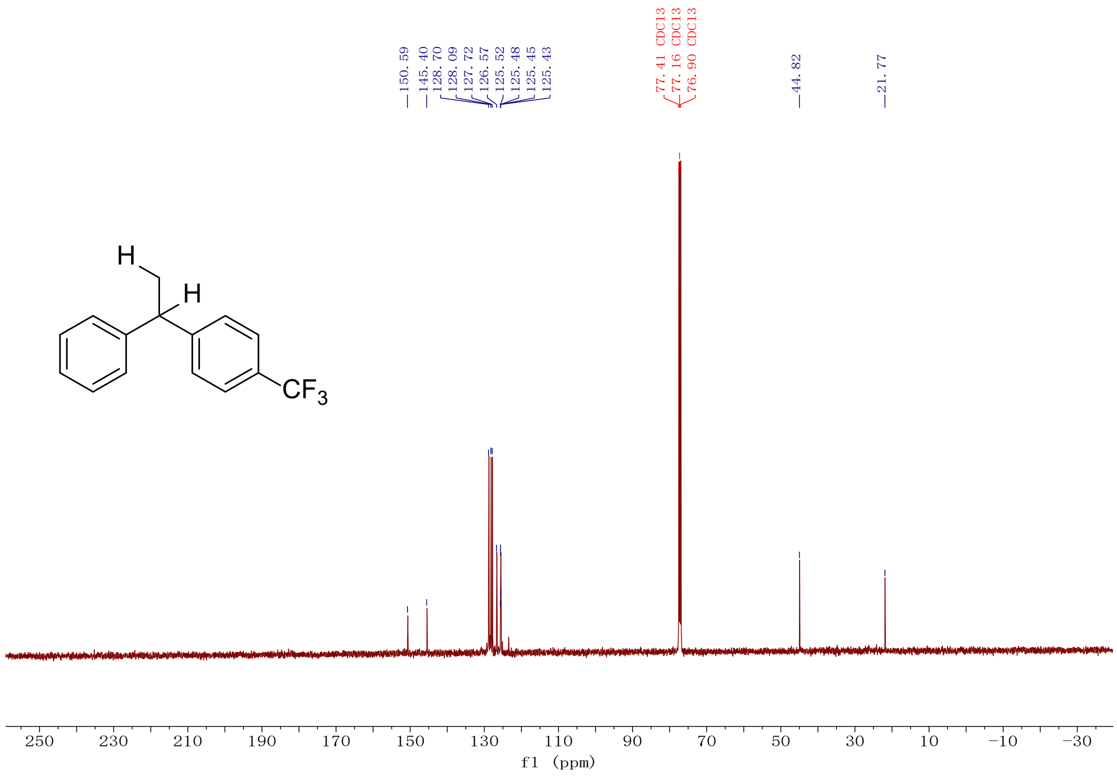
150.59
145.40
128.70
128.09
127.72
126.57
125.52
125.48
125.45
125.43
77.41 CDCl3
77.16 CDCl3
76.90 CDCl3
44.82
21.77
H
H
CF3
250 230 210 190 170 150 130 110 90 70 50 30 10 −10 −30
f1 (ppm)

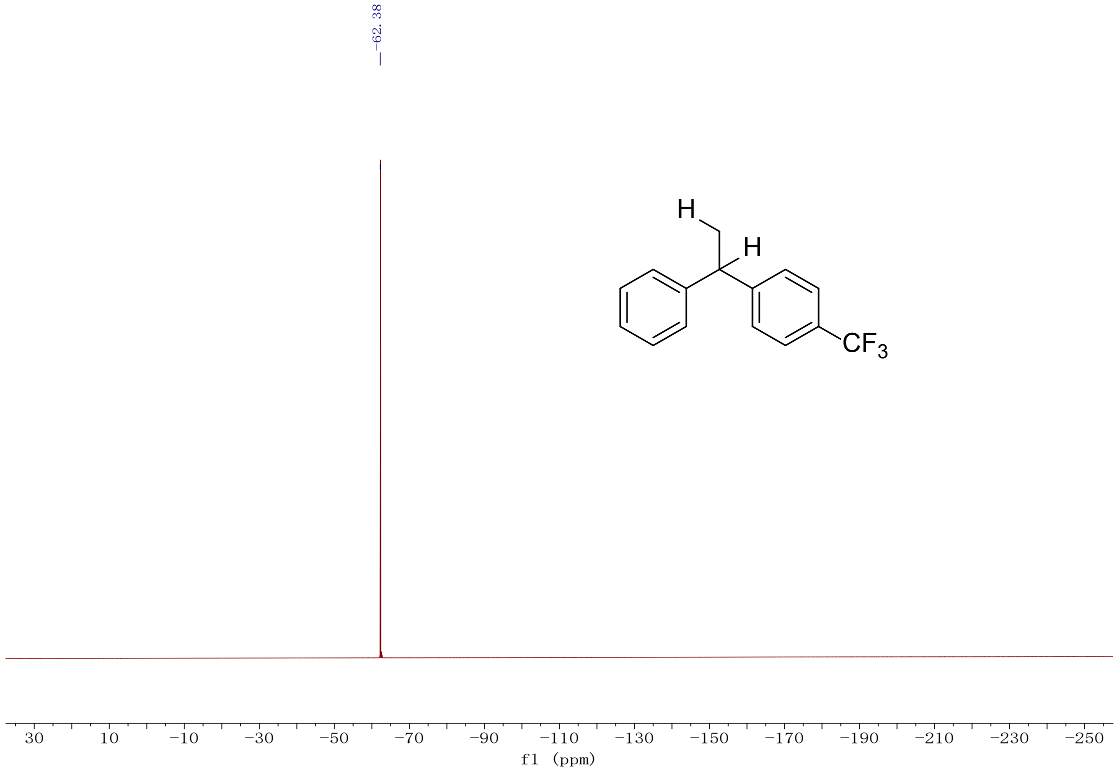
−62.38
H
H
CF3
30 10 −10 −30 −50 −70 −90 −110 −130 −150 −170 −190 −210 −230 −250
f1 (ppm)

4-(1-(4-fluorophenyl)ethyl)-1,1'-biphenyl (8j)

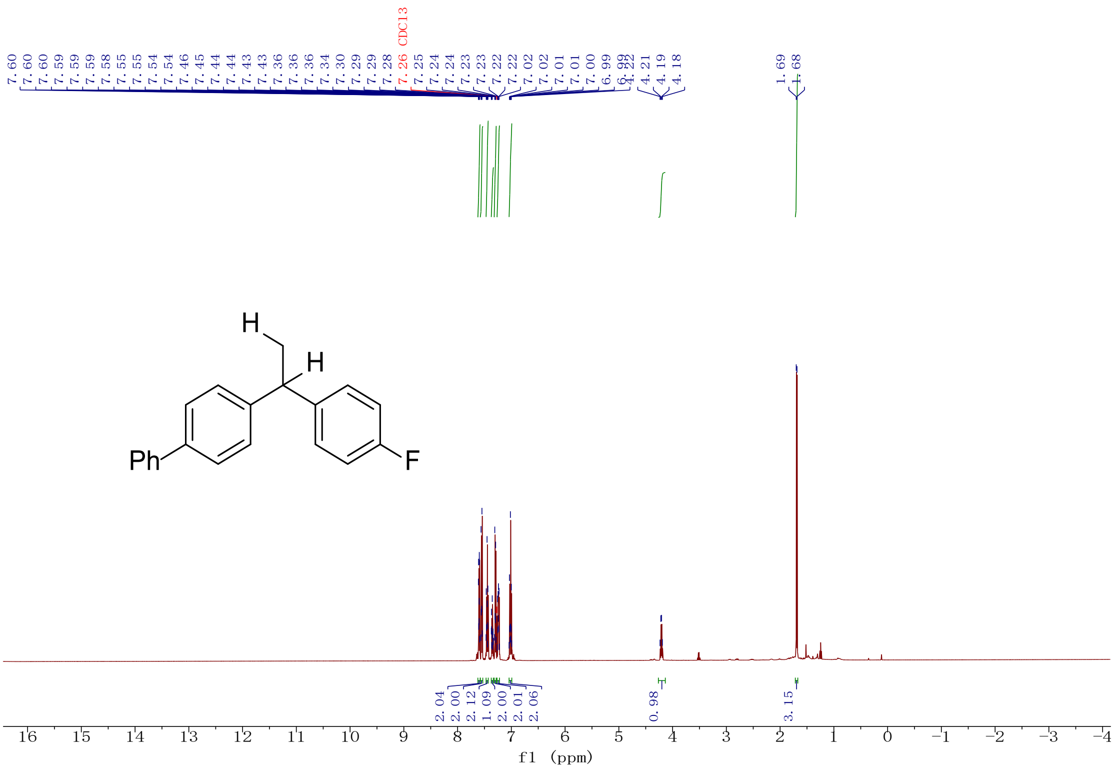

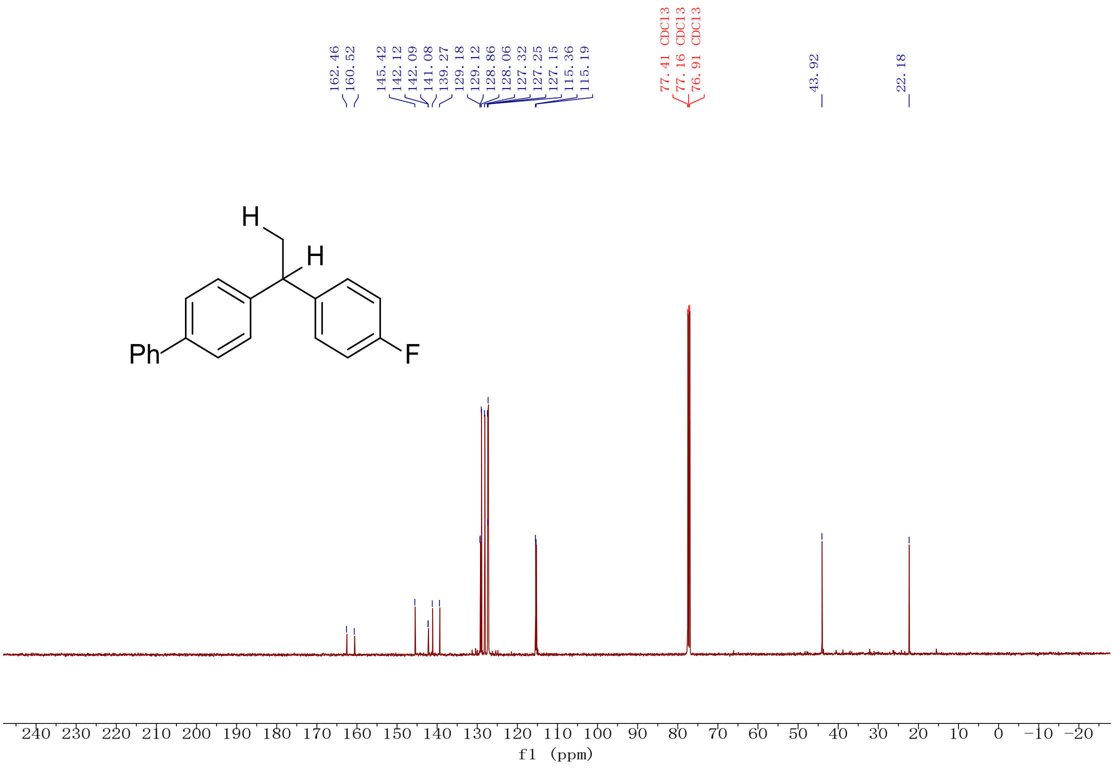

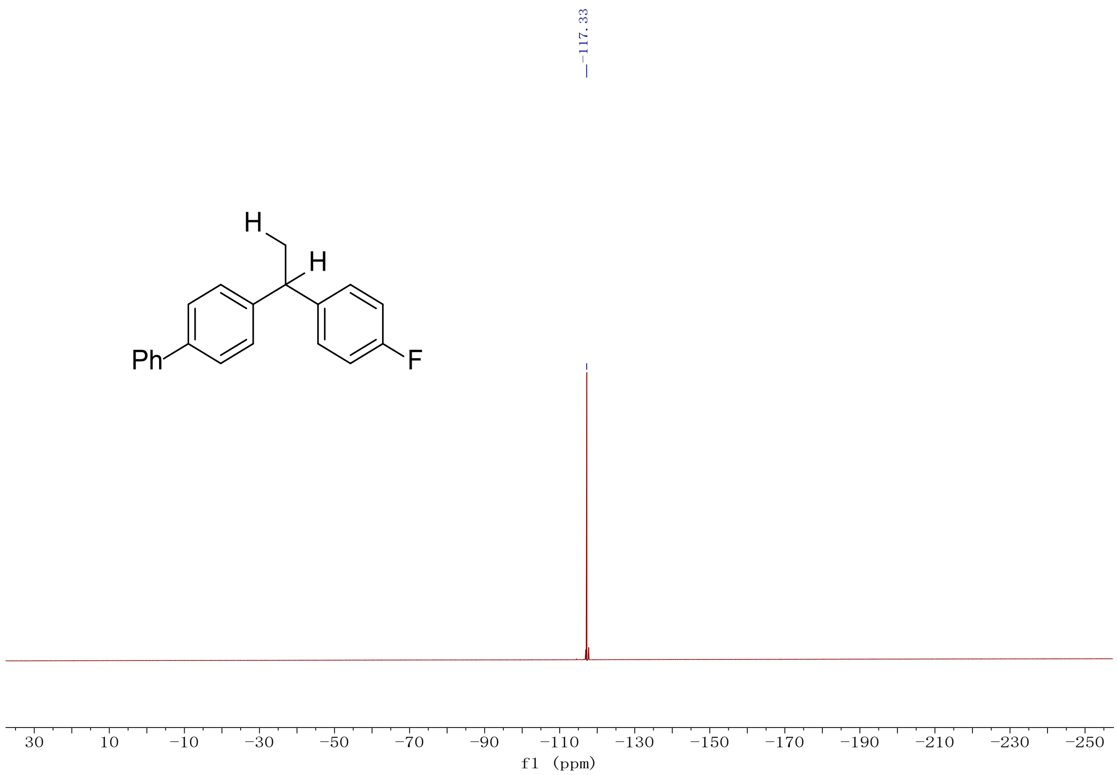

4-(1-Phenylethyl)benzaldehyde (8k)

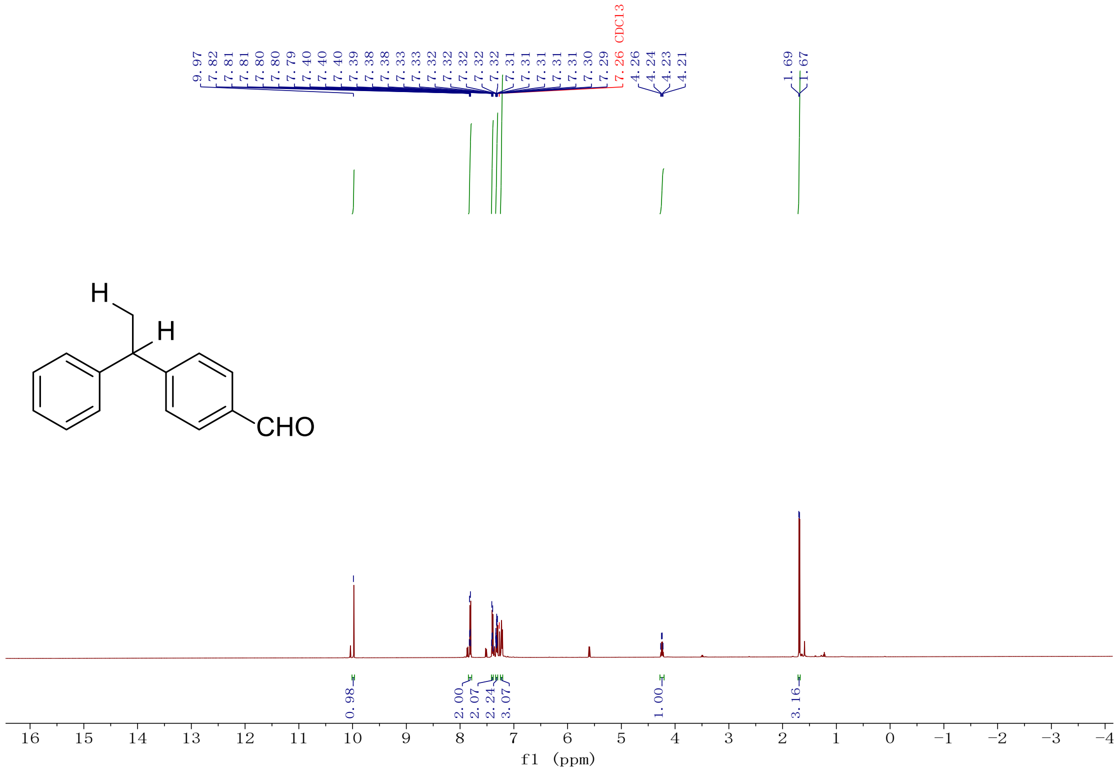

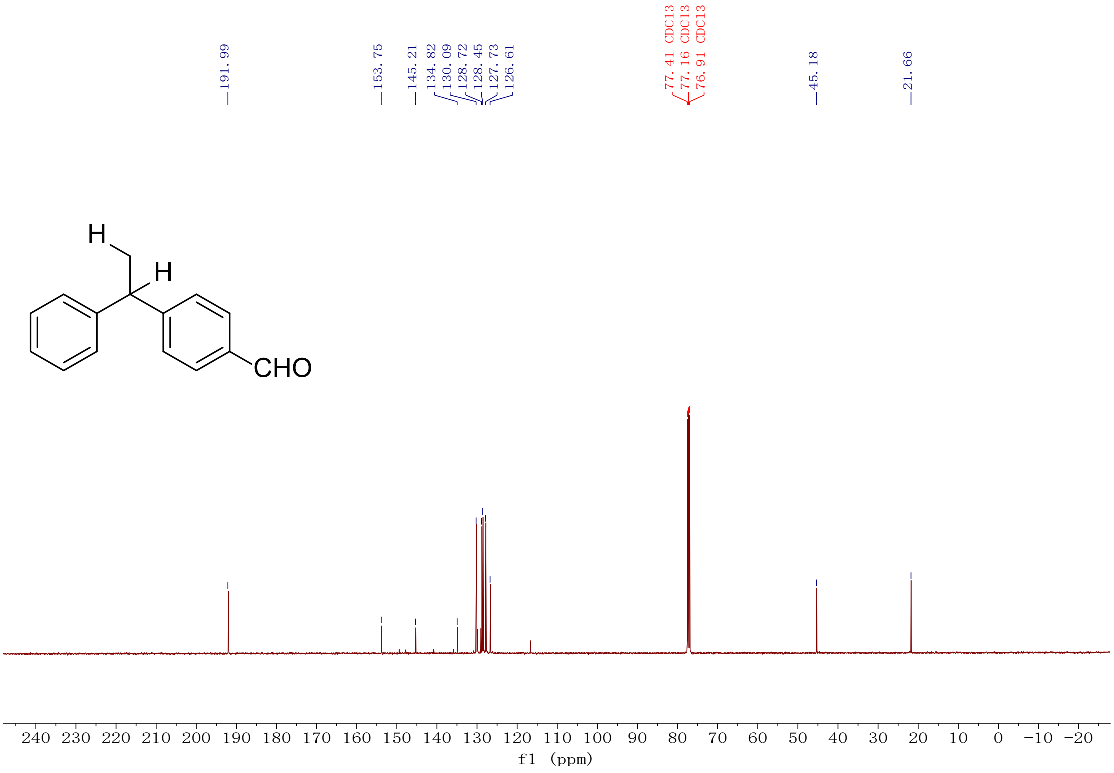

Cyclohexylbenzene (8l)

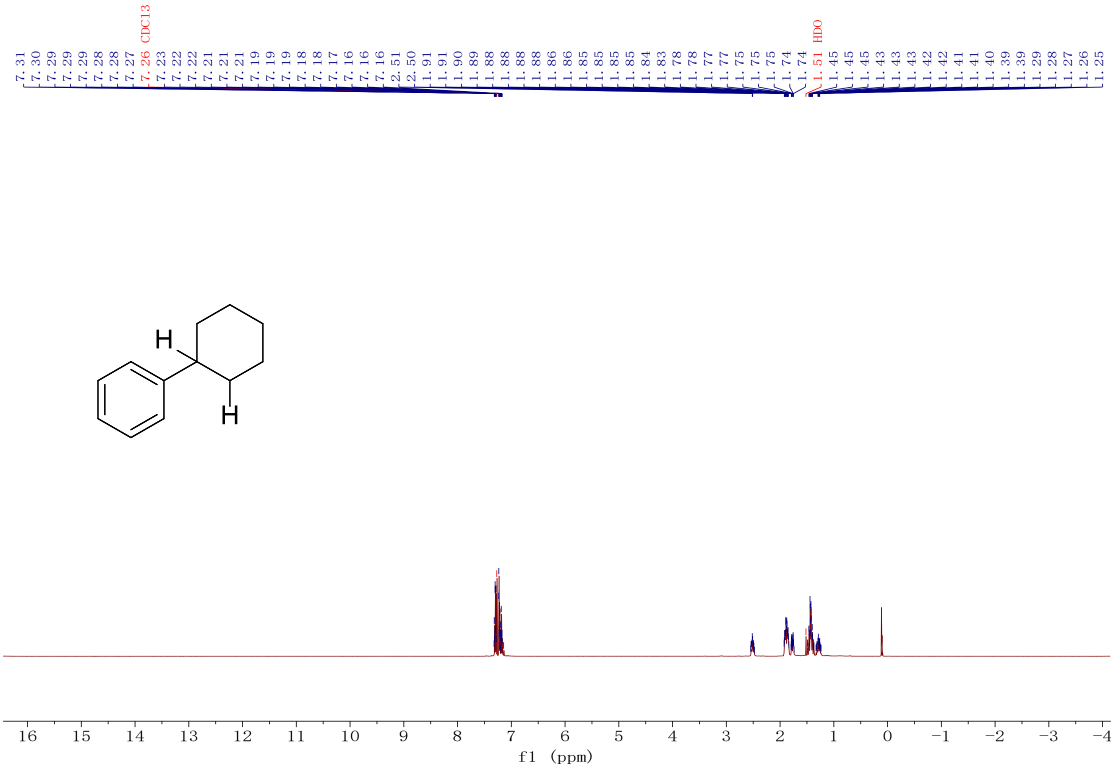

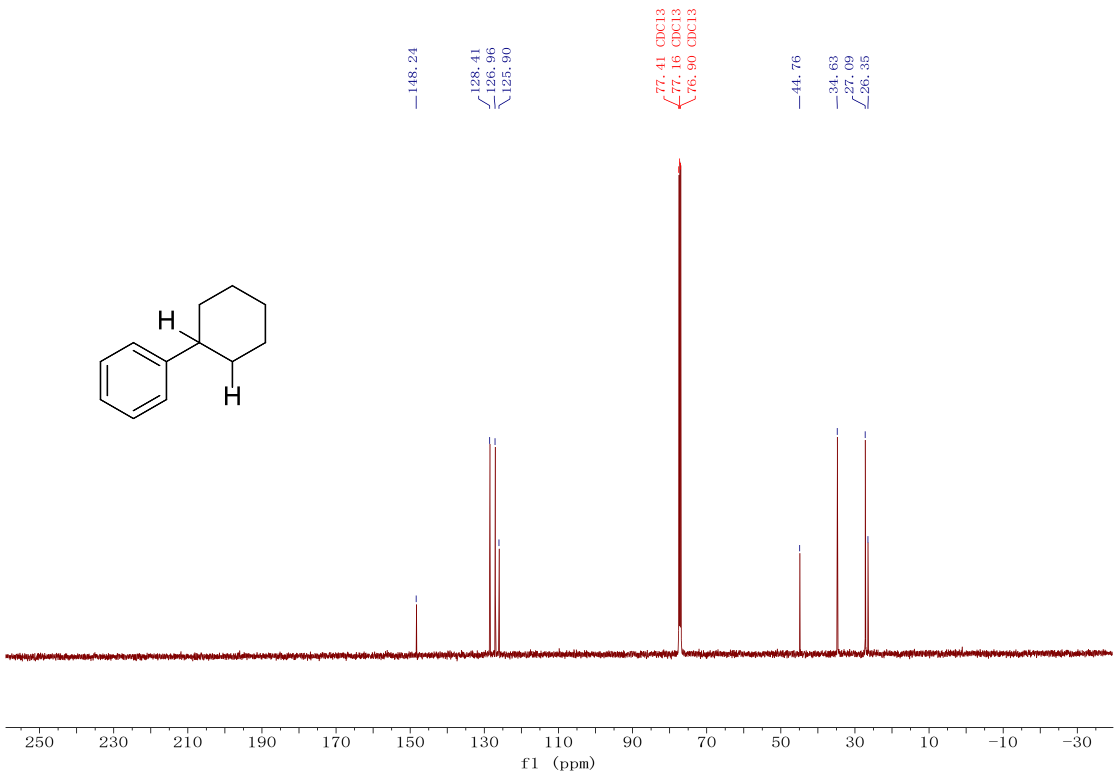

phenylcyclooctane (8m)

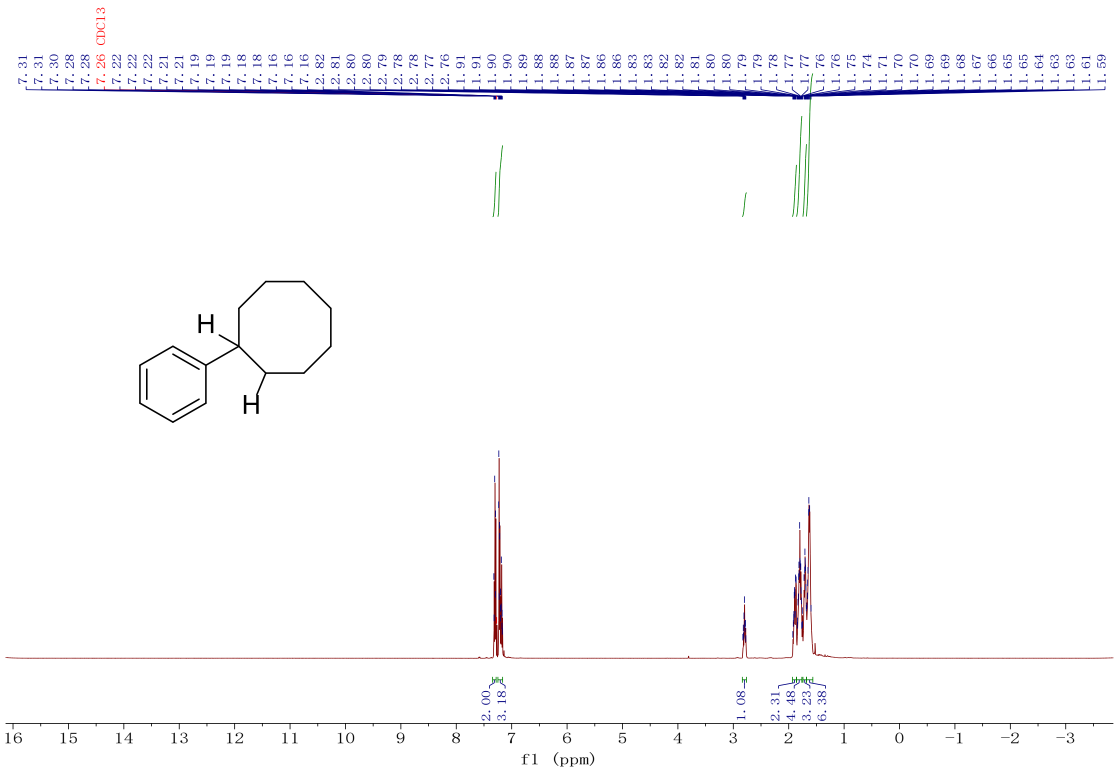

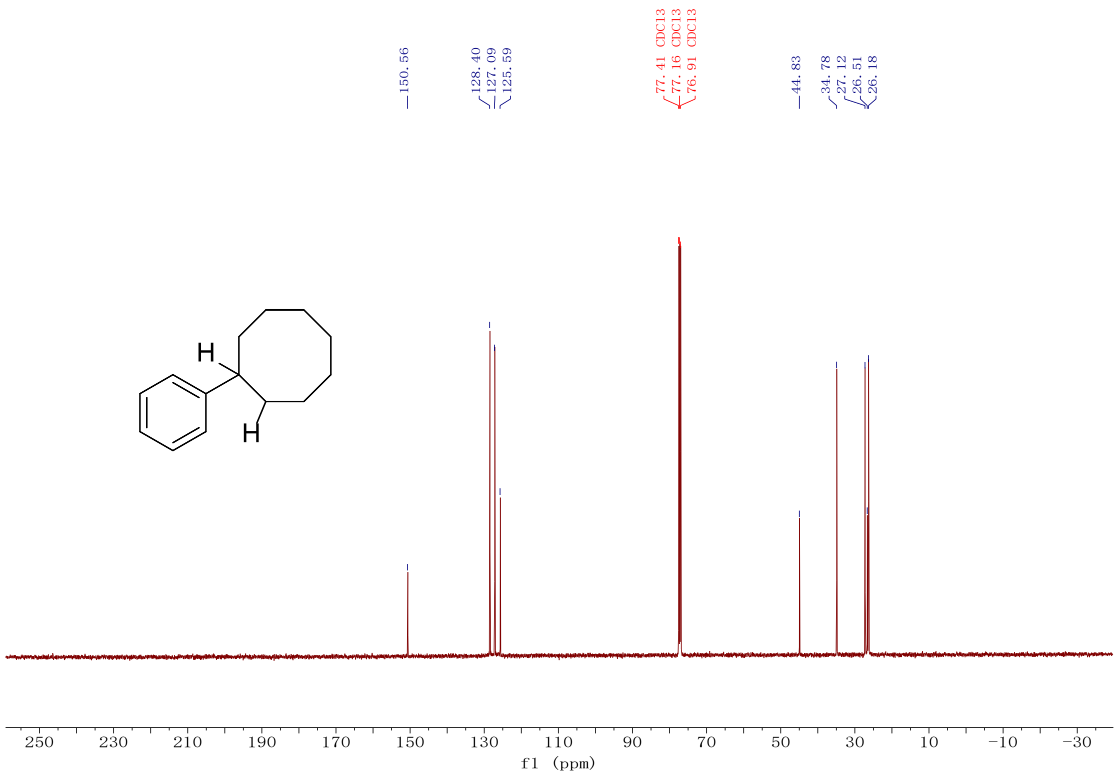

Propane-1,1-diyldibenzene (8n)

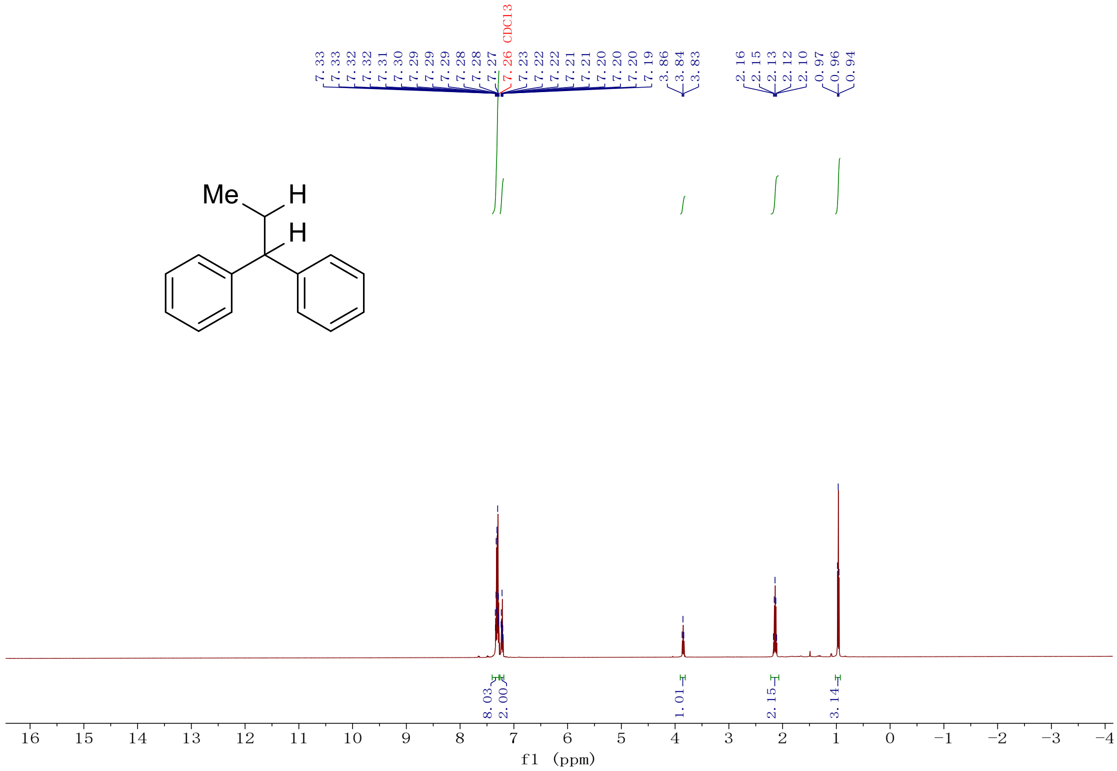

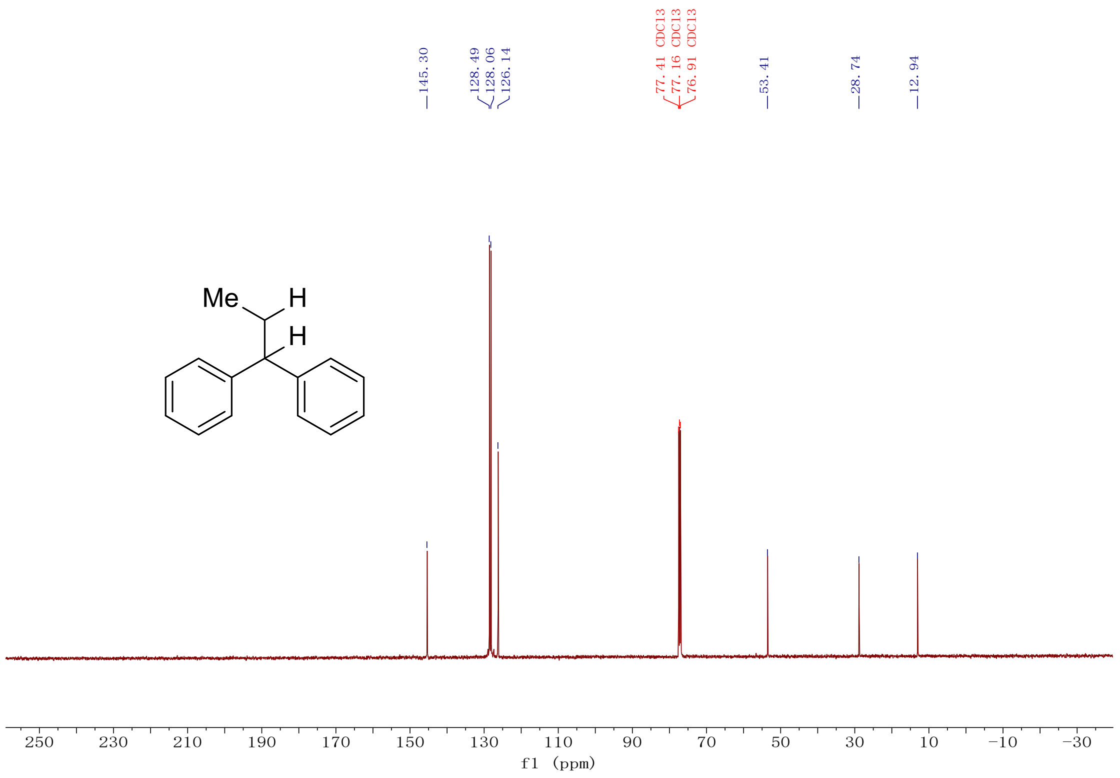

1-methyl-1,2,3,4-tetrahydronaphthalene (8o)

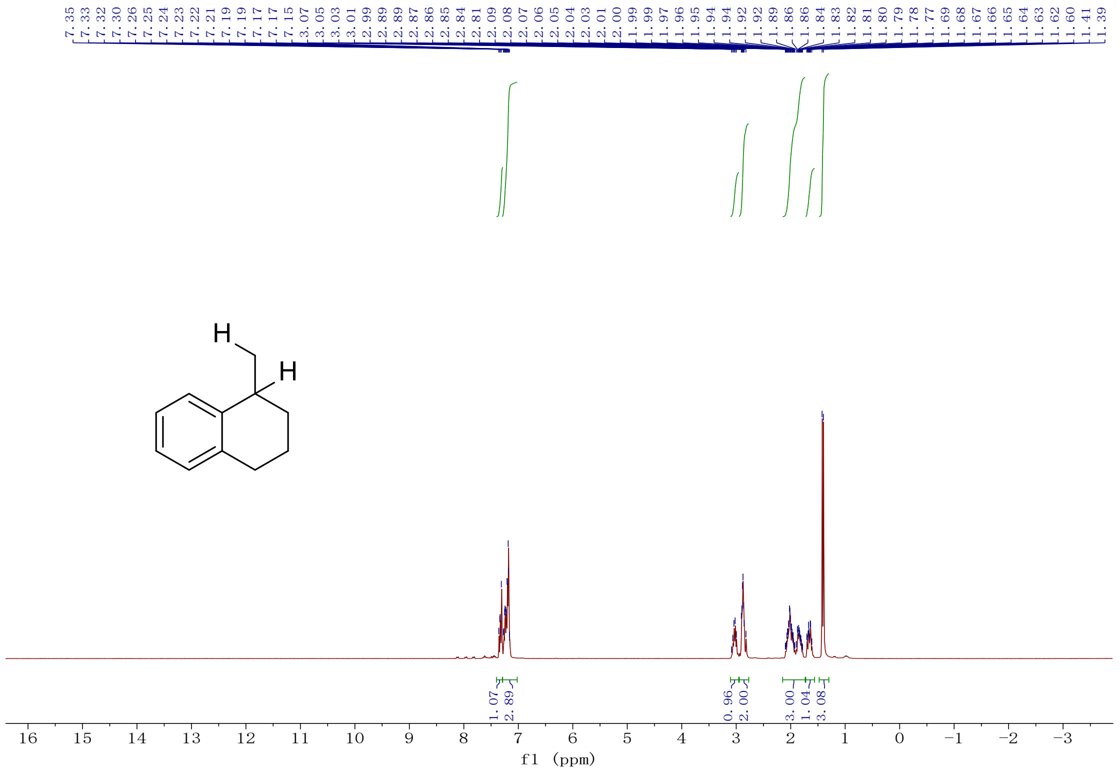

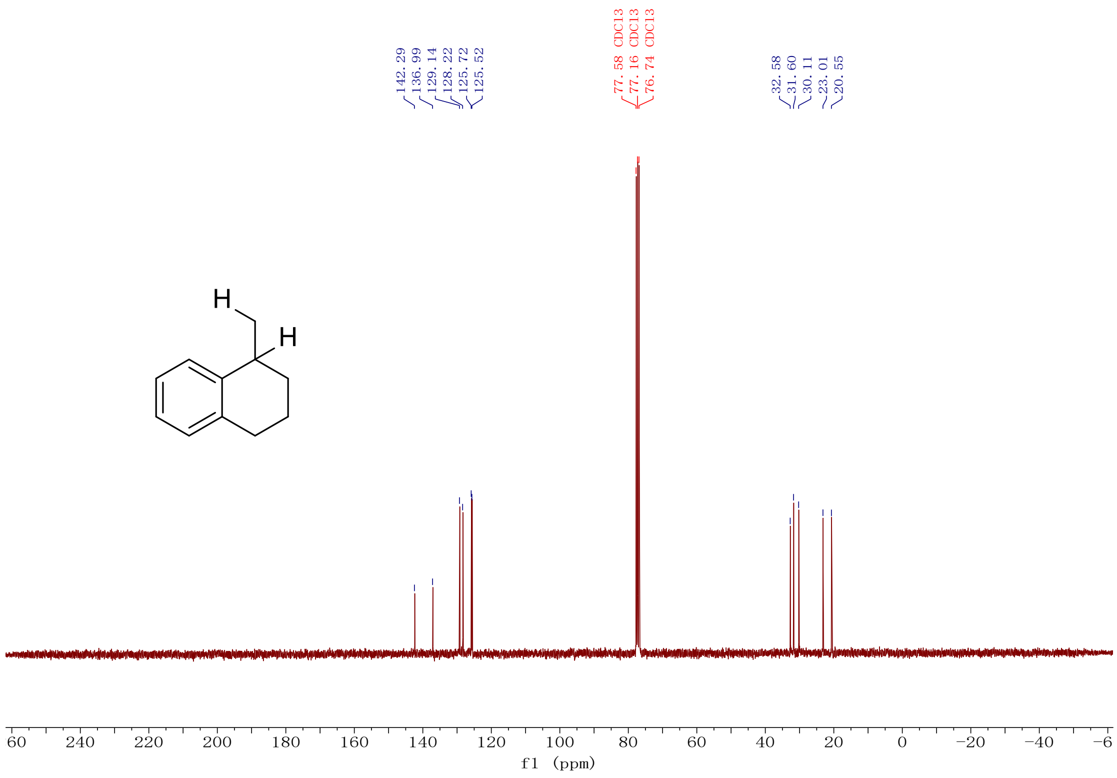

3-Methyldodecane (8p)

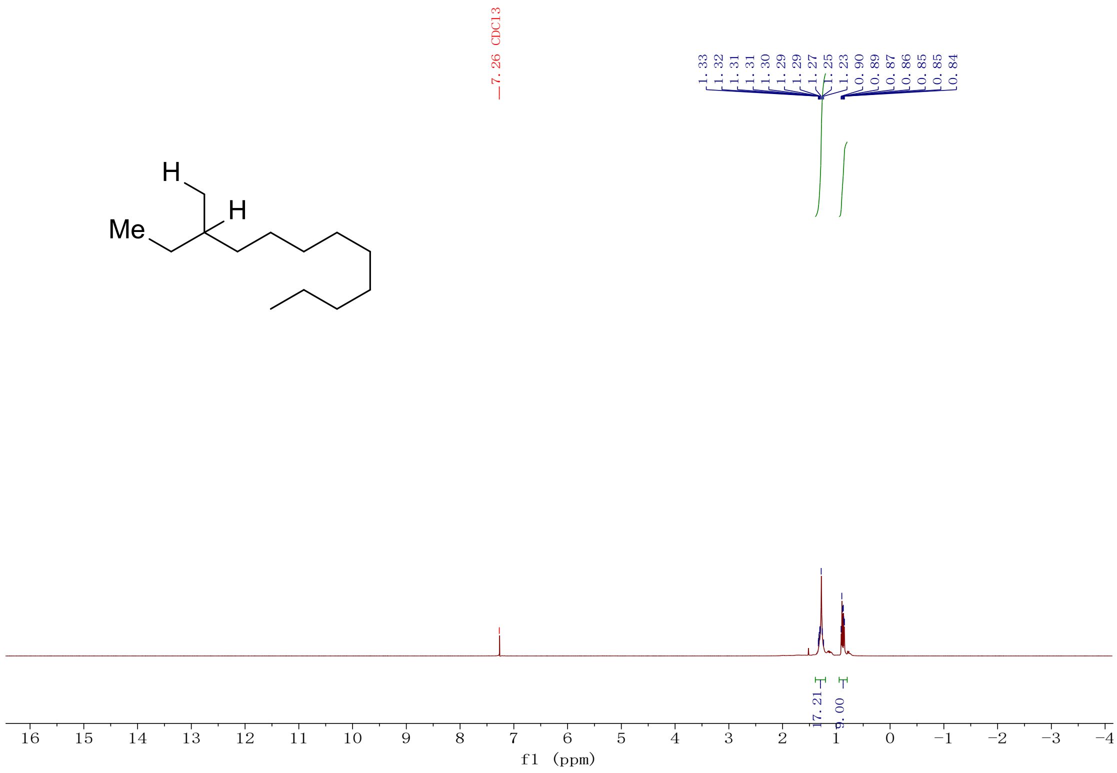

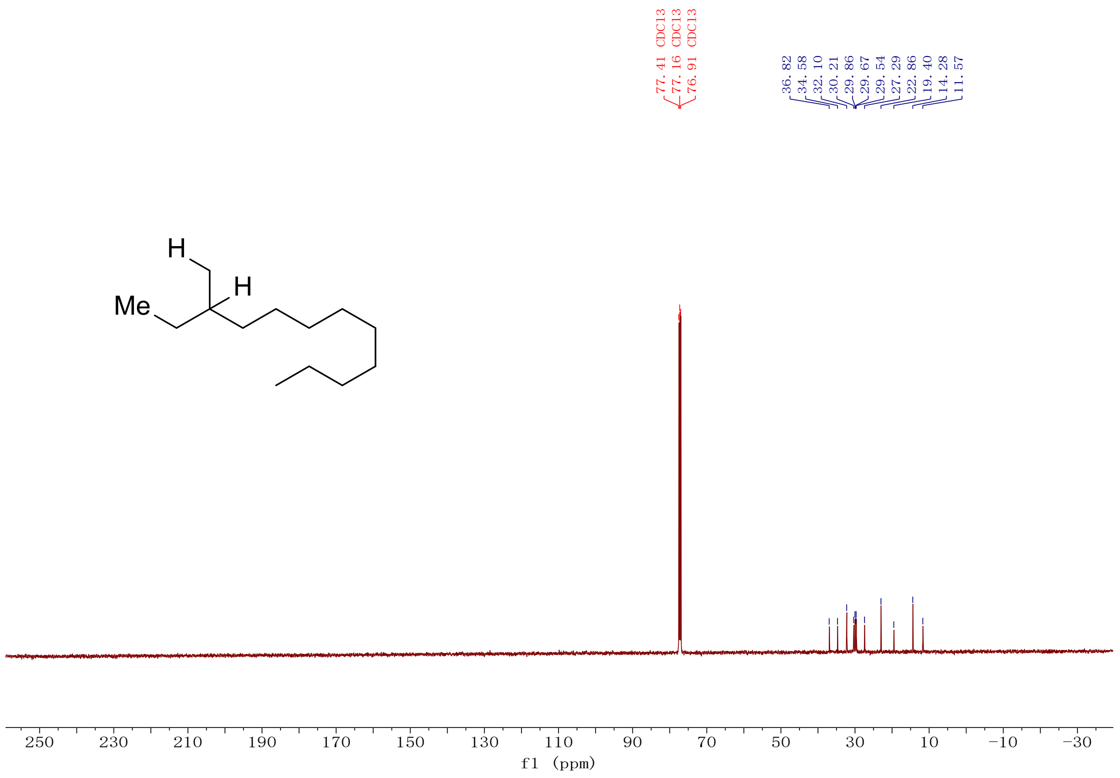

1-Methoxy-4-(1-phenylethyl-1-deutero)benzene (9a)

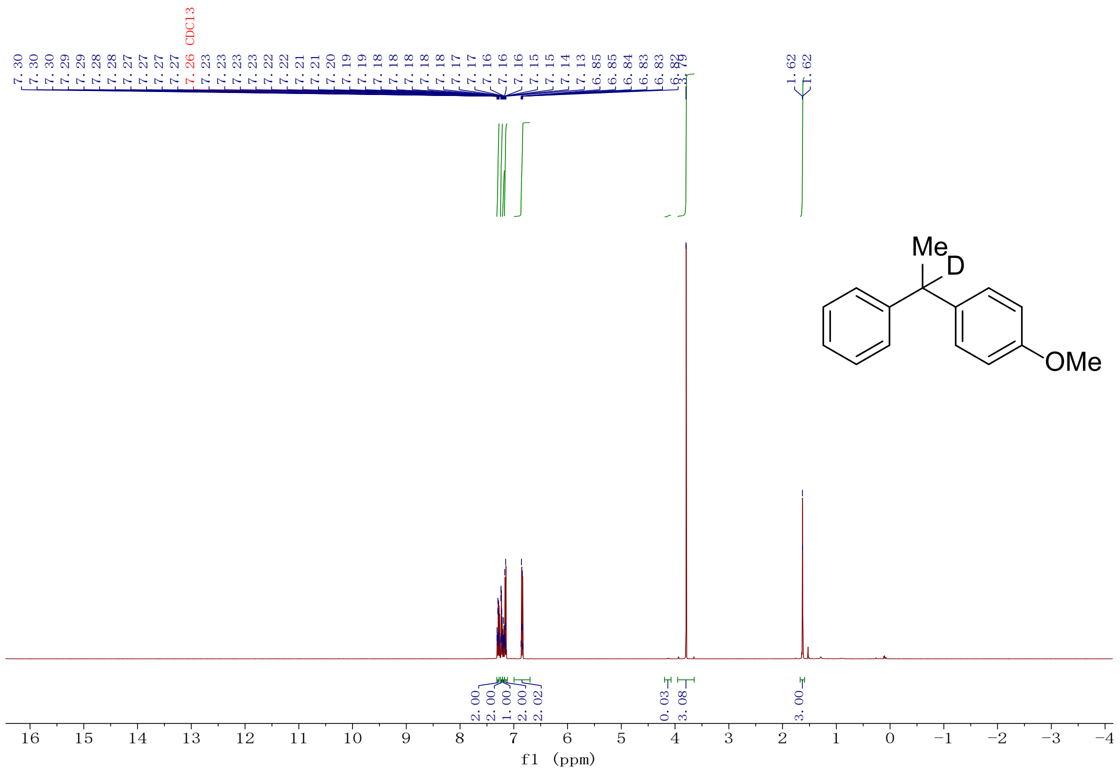

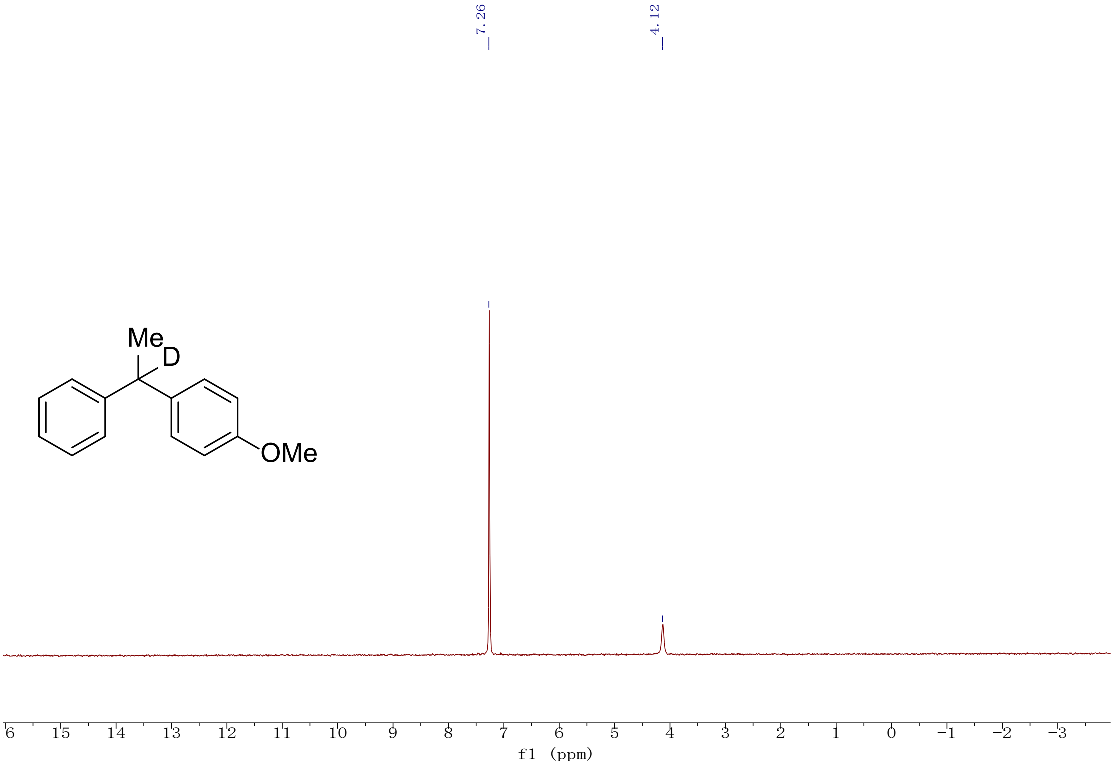
7.26
4.12
Me
D
OMe
6 15 14 13 12 11 10 9 8 7 6 5 4 3 2 1 0 −1 −2 −3
f1 (ppm)

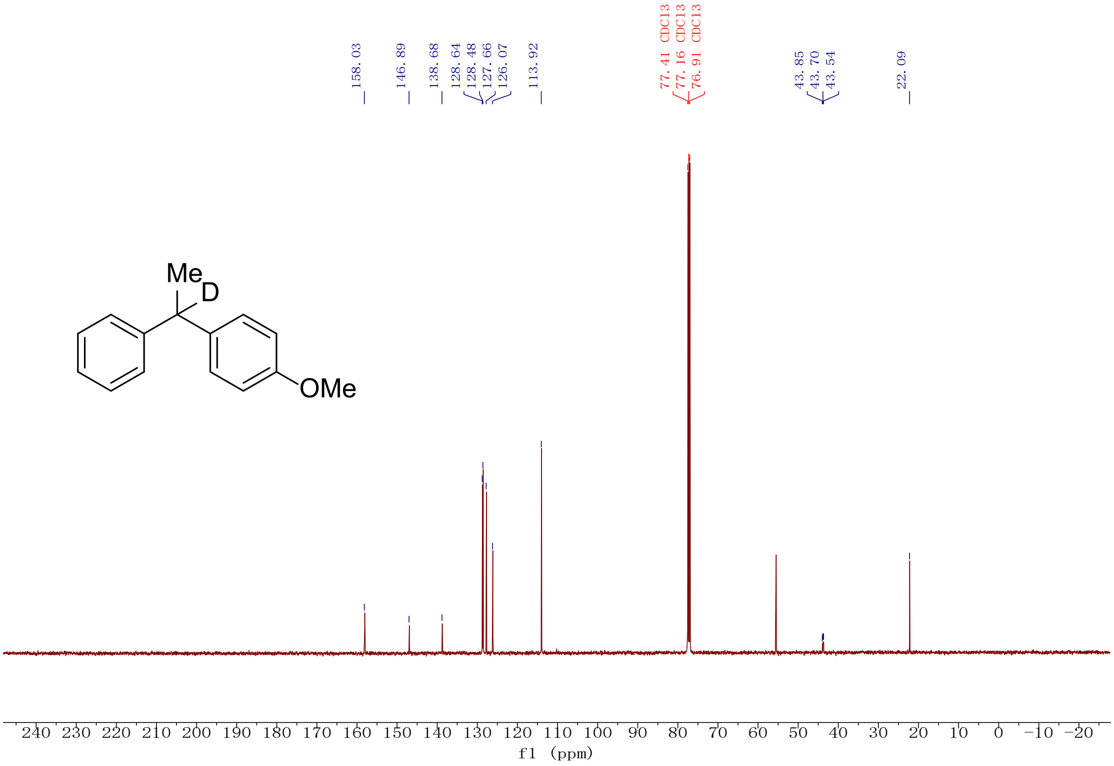
158.03
146.89
138.68
128.64
128.48
127.66
126.07
113.92
77.41 CDCl3
77.16 CDCl3
76.91 CDCl3
43.85
43.70
43.54
22.09
Me
D
OMe
240 230 220 210 200 190 180 170 160 150 140 130 120 110 100 90 80 70 60 50 40 30 20 10 0 −10 −20
f1 (ppm)

4-(1-(4-Fluorophenyl)ethyl-1-deutero)-1,1'-biphenyl (9b)

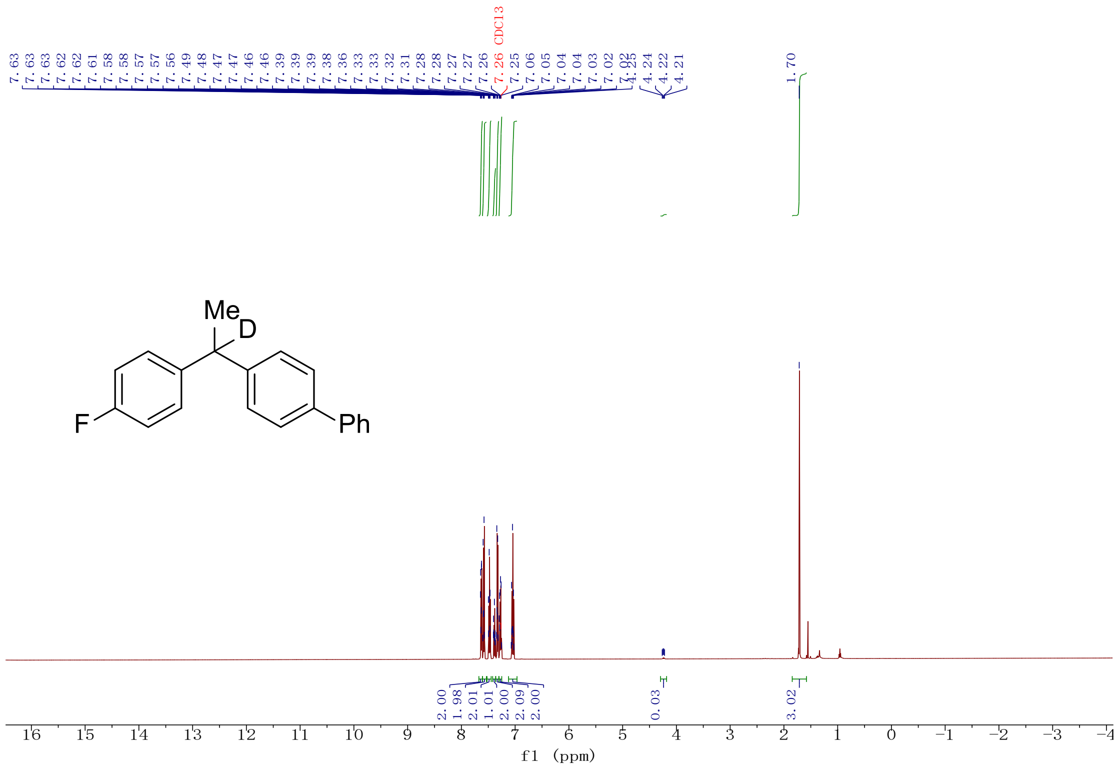

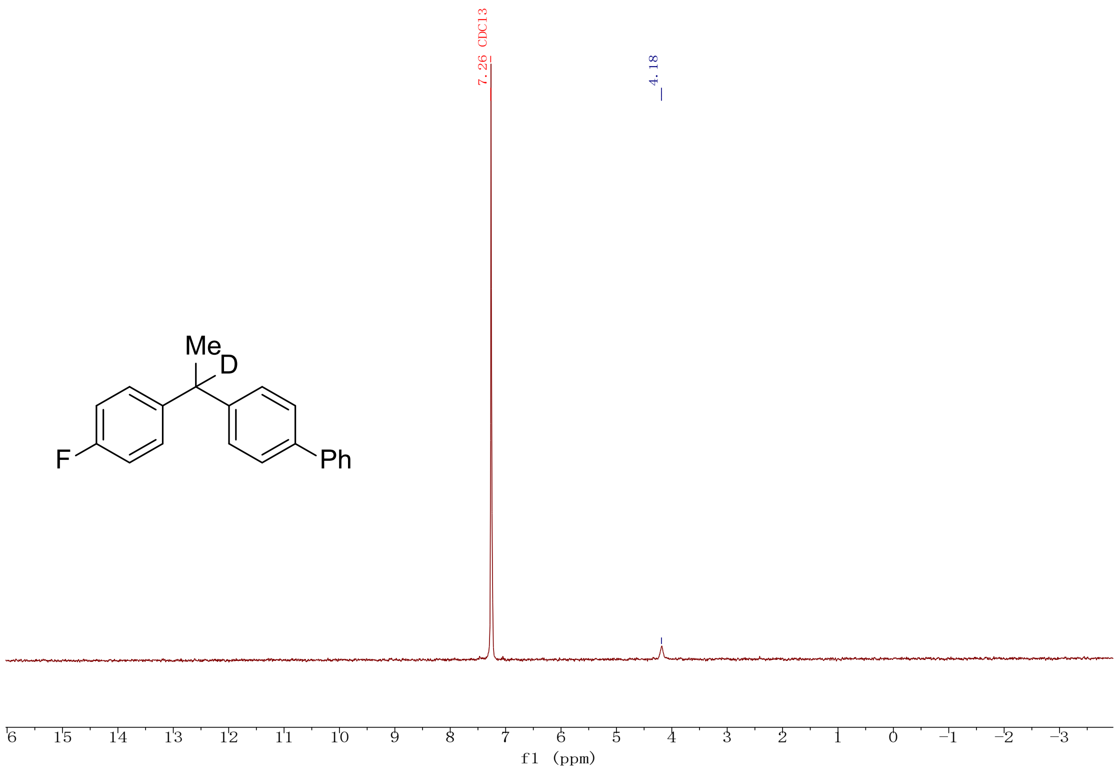

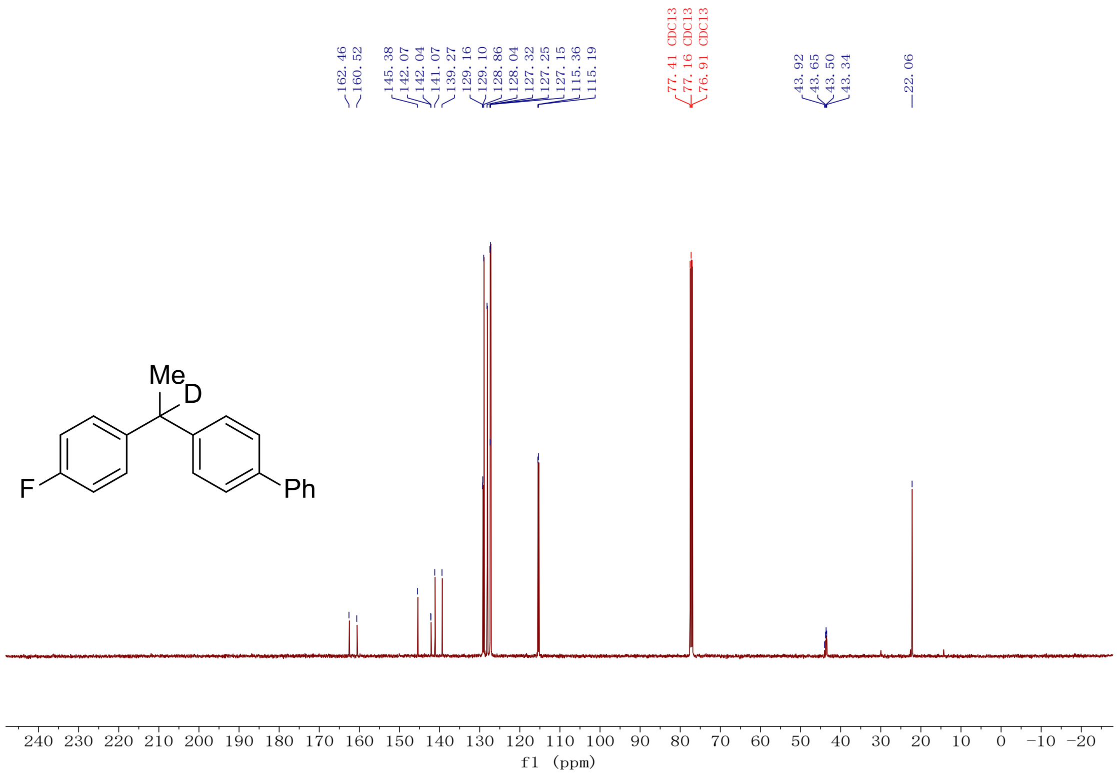
162.46
160.52
145.38
142.07
142.04
141.07
139.27
129.16
129.10
128.86
128.04
127.32
127.25
127.15
115.36
115.19
77.41 CDCl3
77.16 CDCl3
76.91 CDCl3
43.92
43.65
43.50
43.34
22.06
Me
D
F
Ph
240 230 220 210 200 190 180 170 160 150 140 130 120 110 100 90 80 70 60 50 40 30 20 10 0 −10 −20
f1 (ppm)

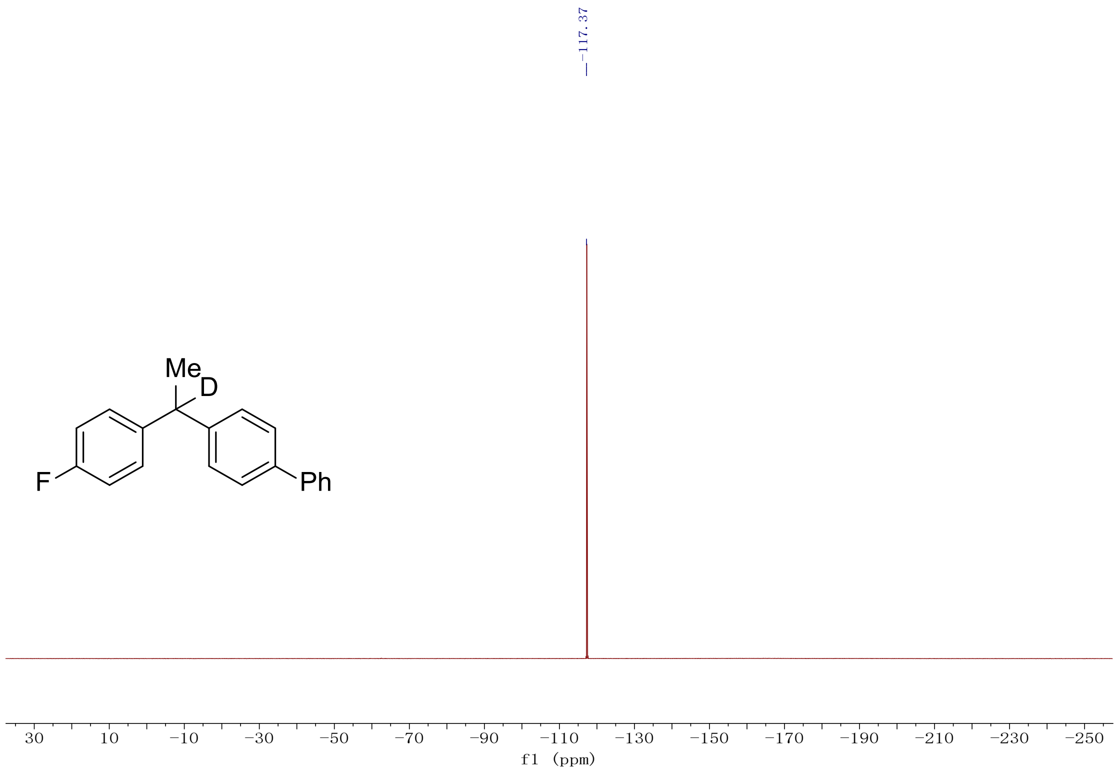
−117.37
Me
D
F
Ph
30 10 −10 −30 −50 −70 −90 −110 −130 −150 −170 −190 −210 −230 −250
f1 (ppm)

(Cyclohexyl-1-deutero)benzene (9c)

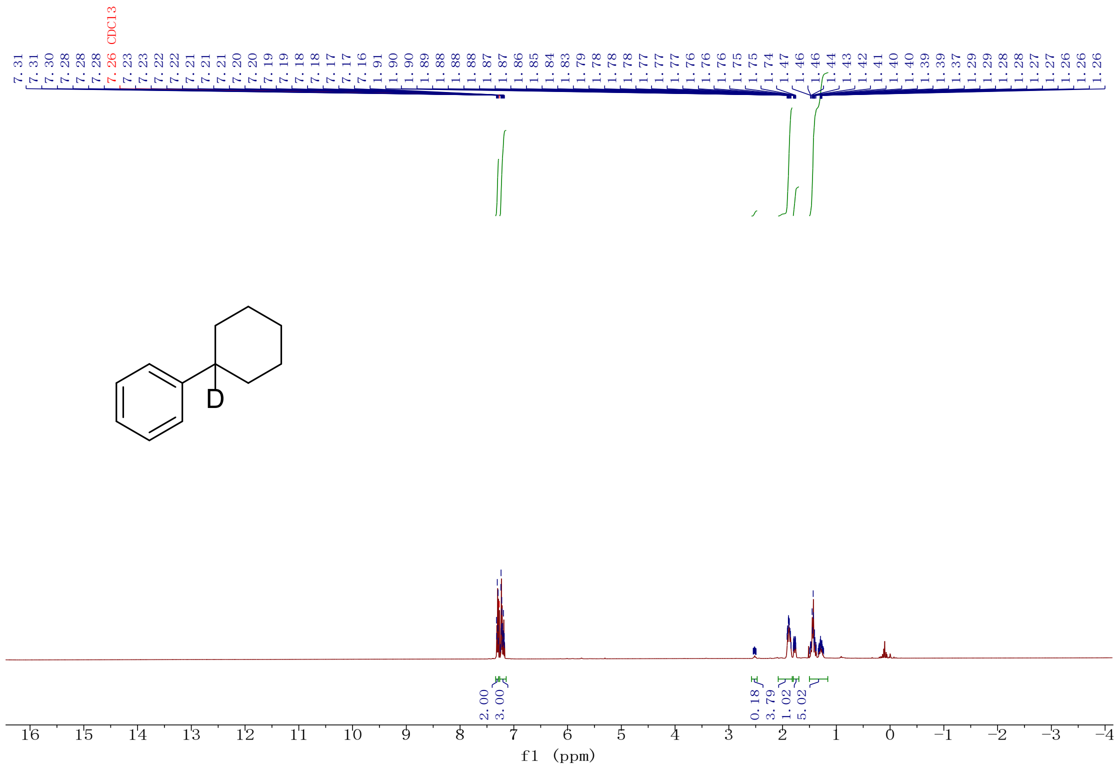

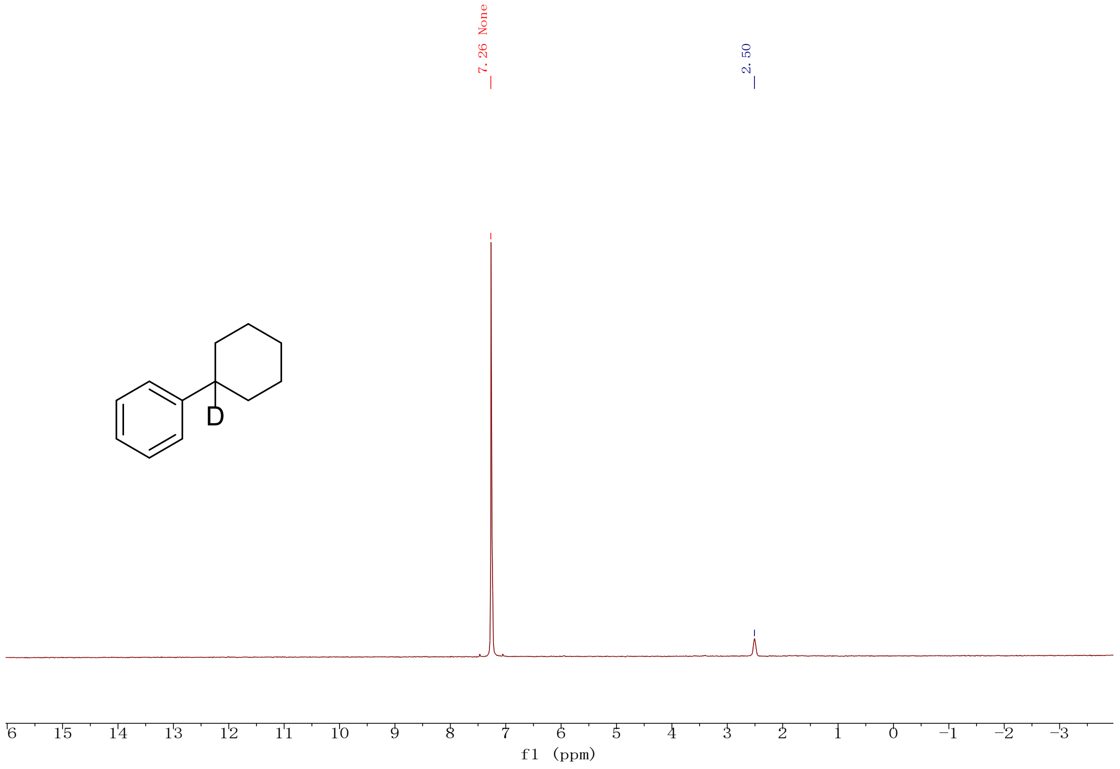

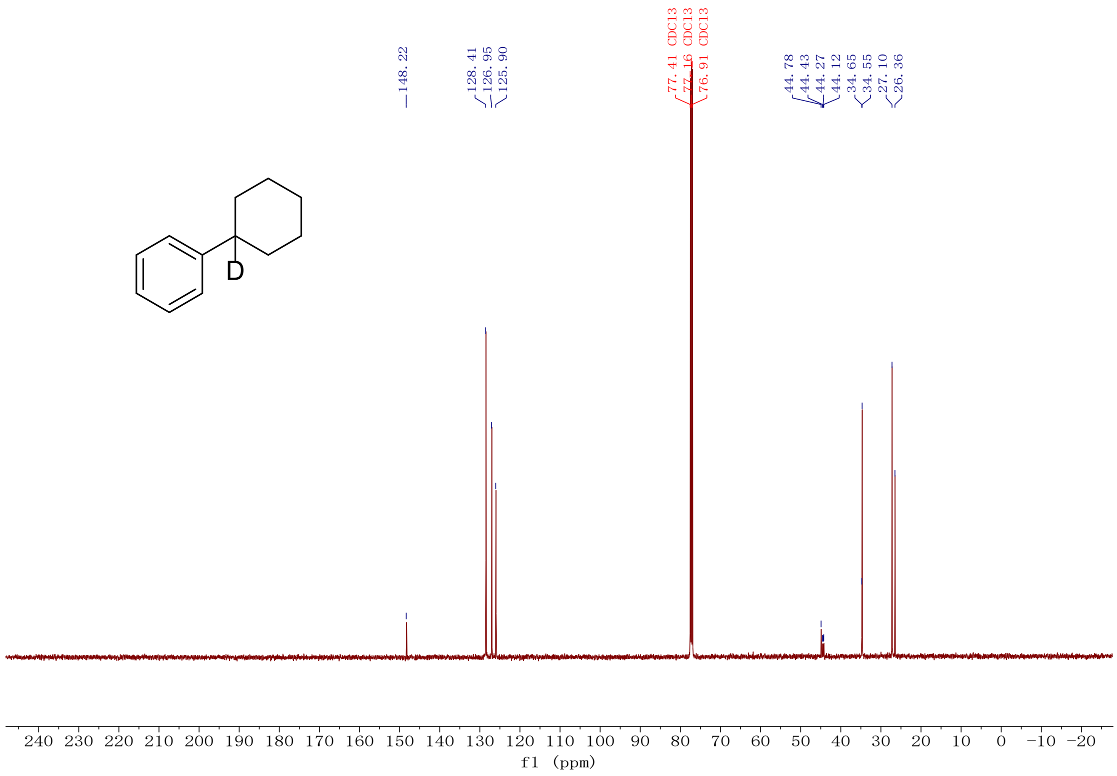

(Propane-1,1-diyl-1- deutero)dibenzene (9d)

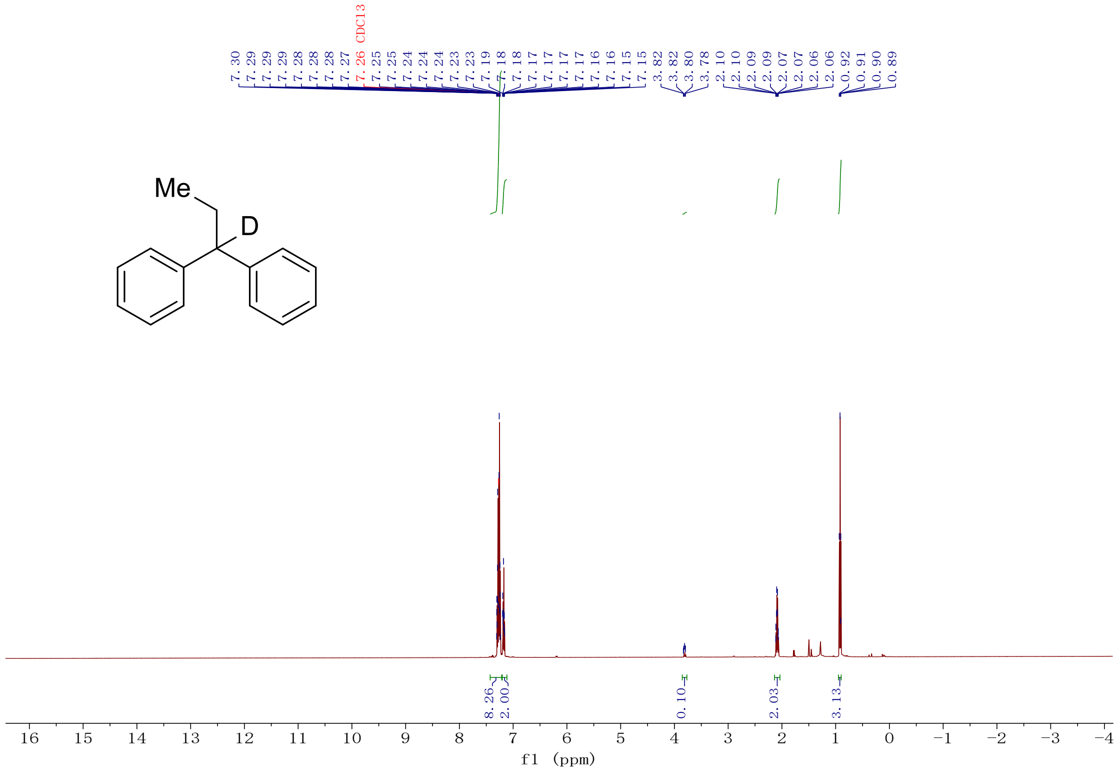

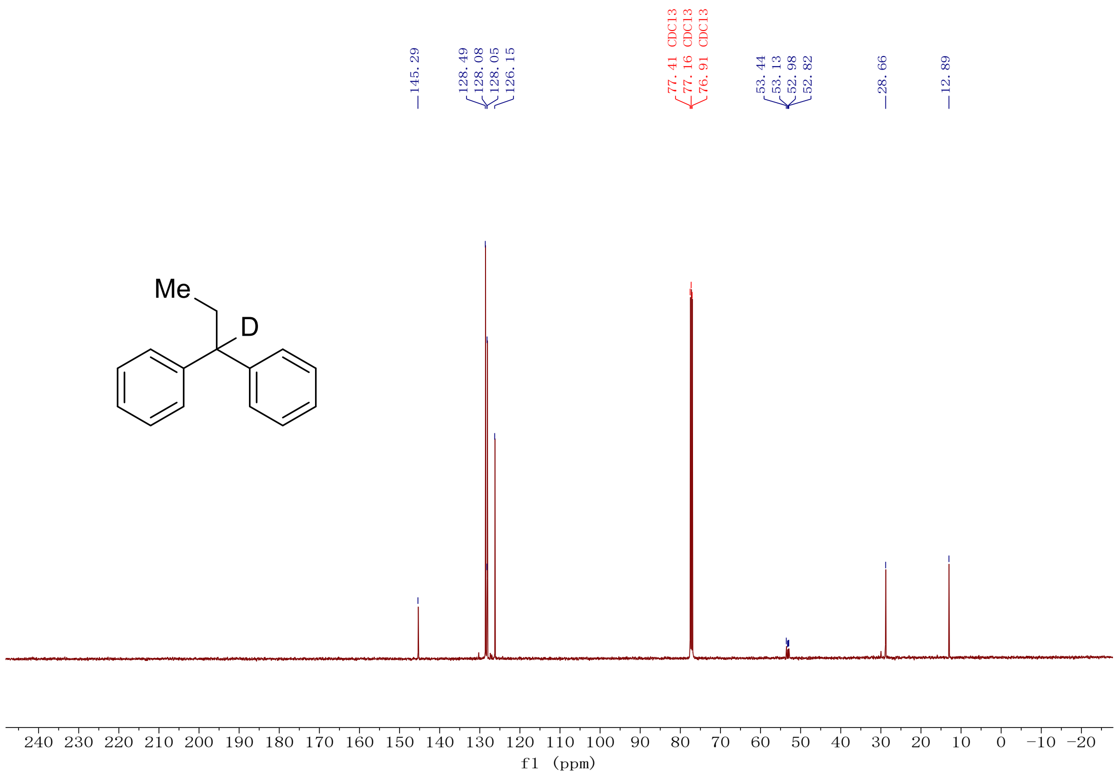
145.29
128.49
128.08
128.05
126.15
77.41 CDCl3
77.16 CDCl3
76.91 CDCl3
53.44
53.13
52.98
52.82
28.66
12.89
Me
D
f1 (ppm)

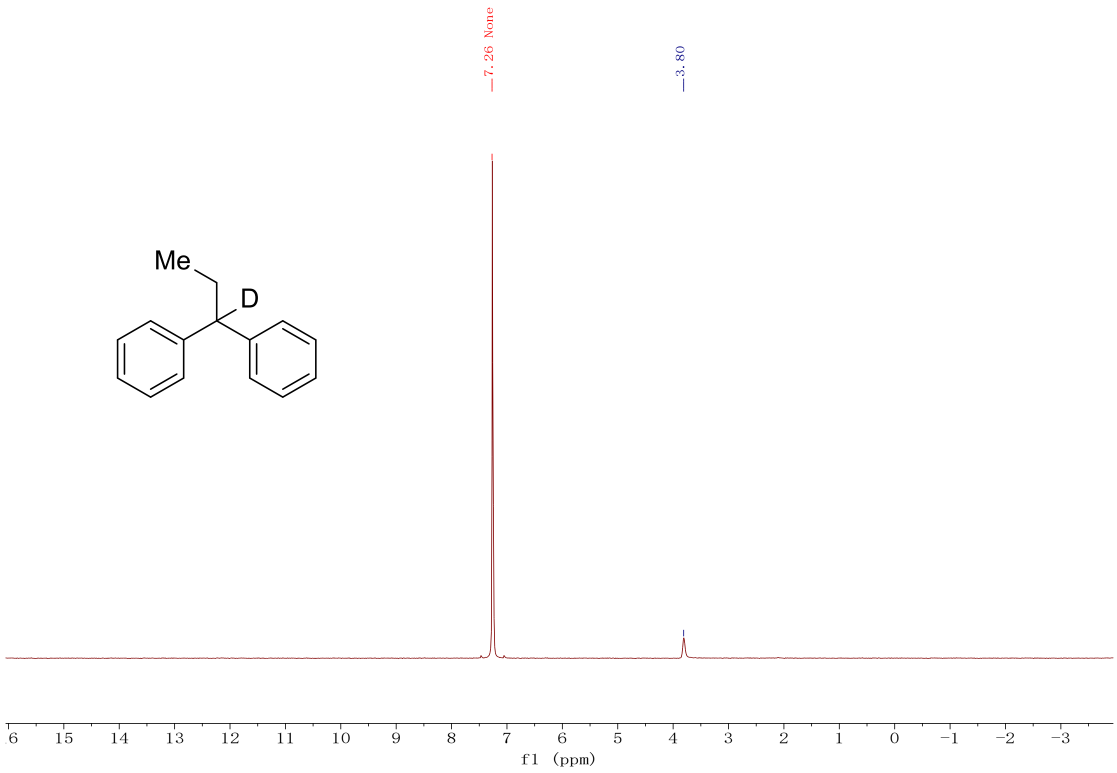
7.26 None
3.80
Me
D
f1 (ppm)

1-Methoxy-4-(2-deutero-1-phenylethyl)benzene (10a)

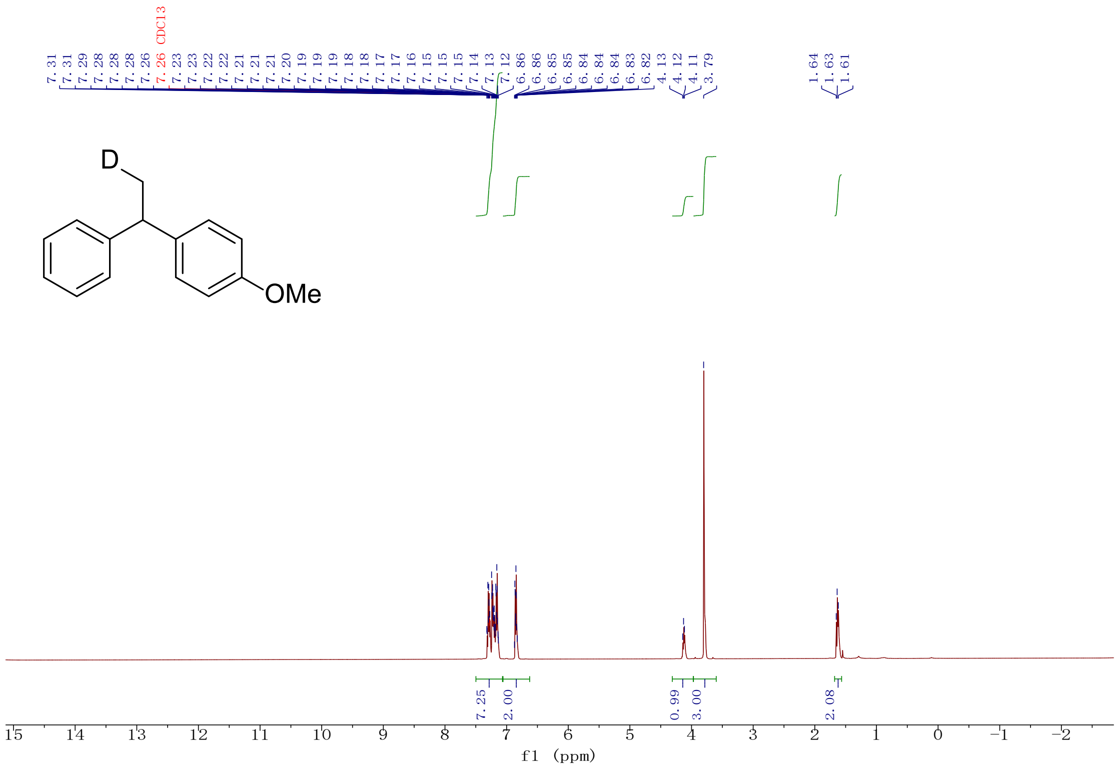

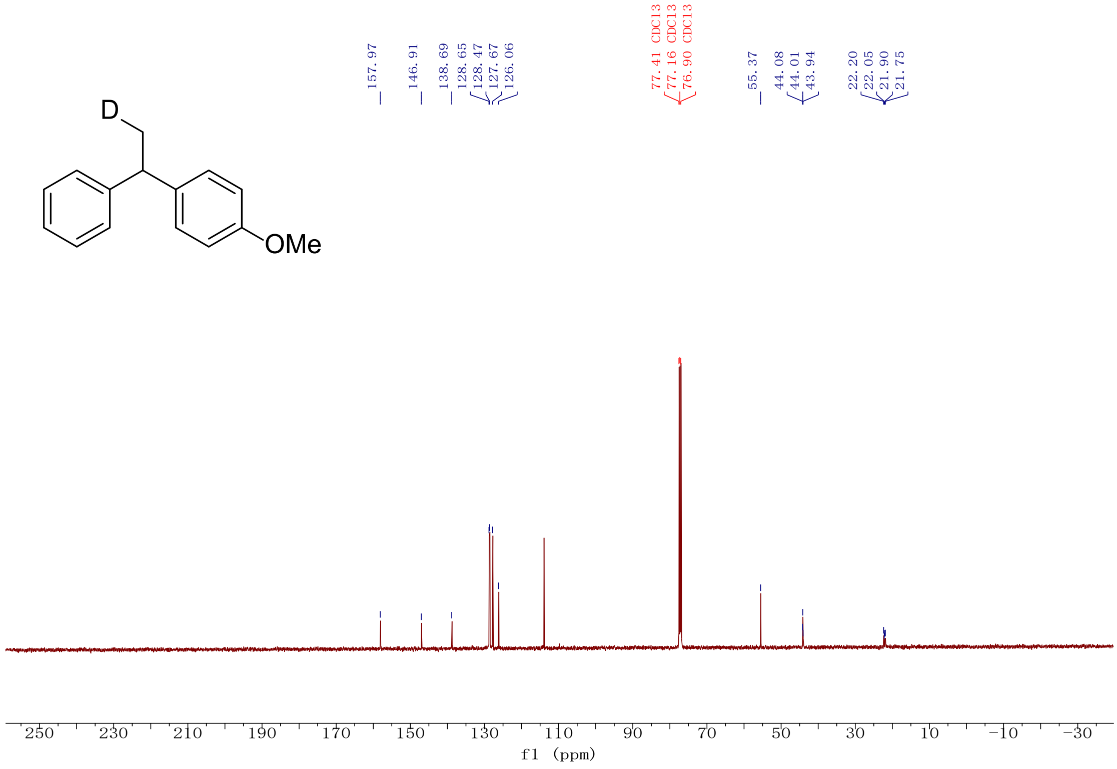

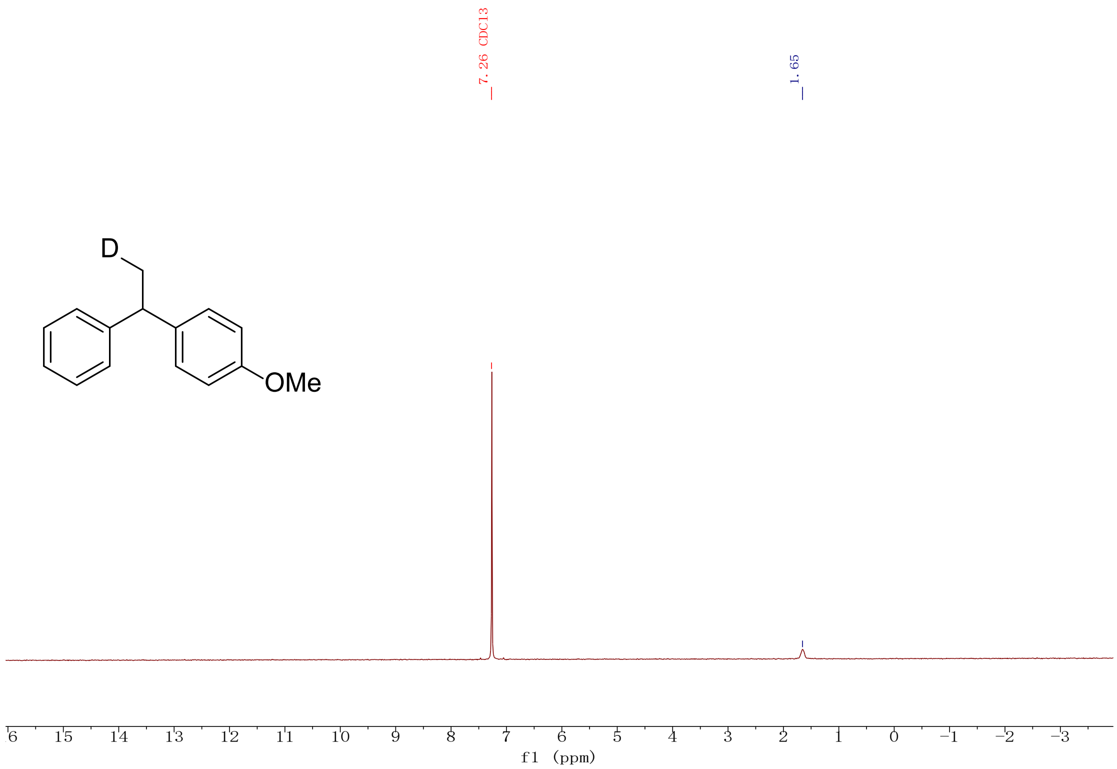

4-(1-(4-Fluorophenyl)ethyl-2-deutero)-1,1'-biphenyl (10b)

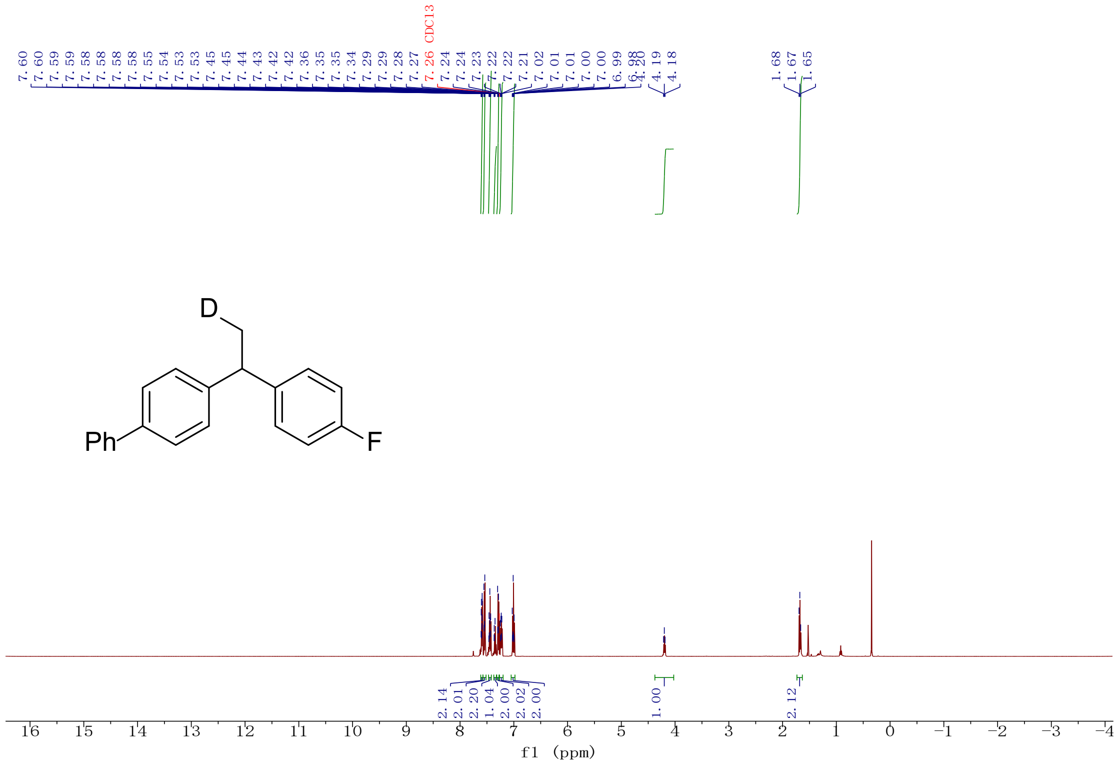

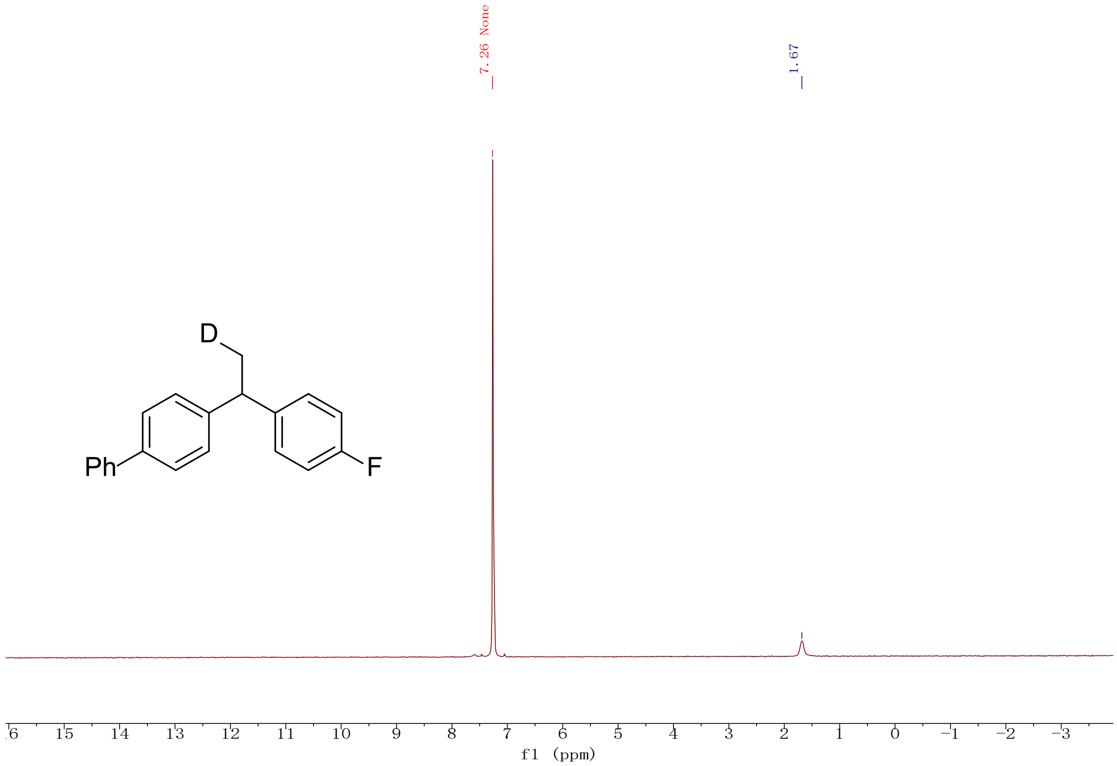
7.26 None
1.67
D
Ph
F
f1 (ppm)

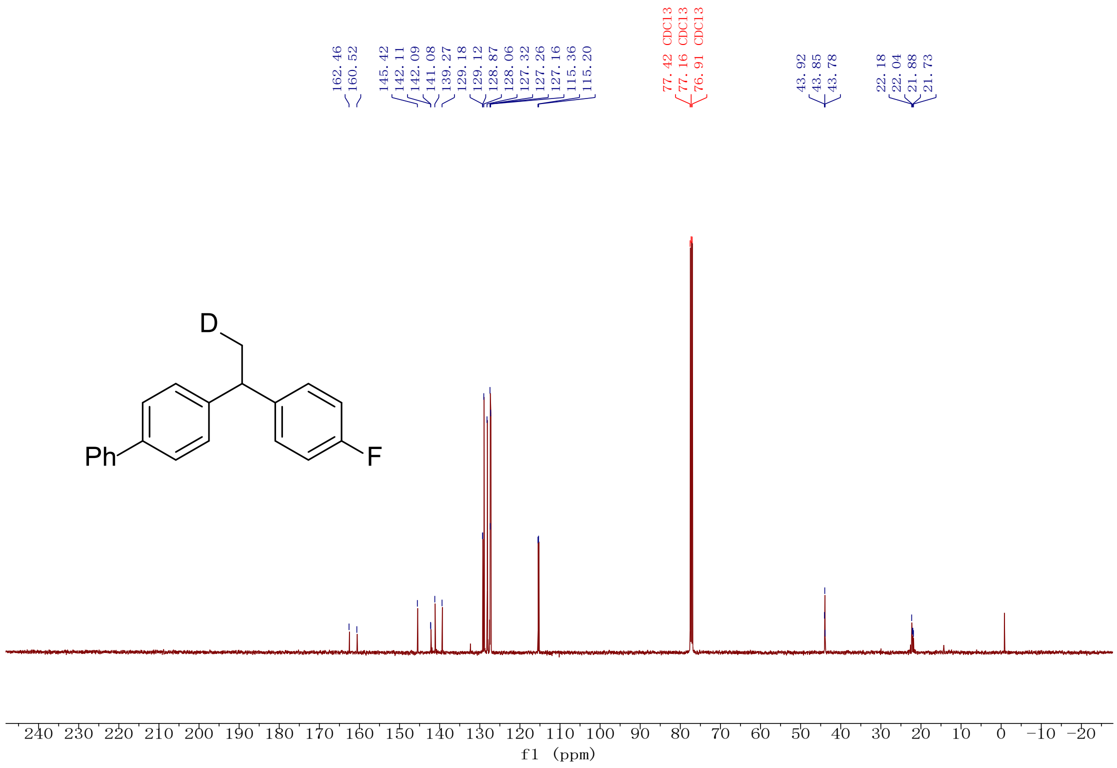
162.46
160.52
145.42
142.11
142.09
141.08
139.27
129.18
129.12
128.87
128.06
127.32
127.26
127.16
115.36
115.20
77.42 CDCl3
77.16 CDCl3
76.91 CDCl3
43.92
43.85
43.78
22.18
22.04
21.88
21.73
D
Ph
F
f1 (ppm)

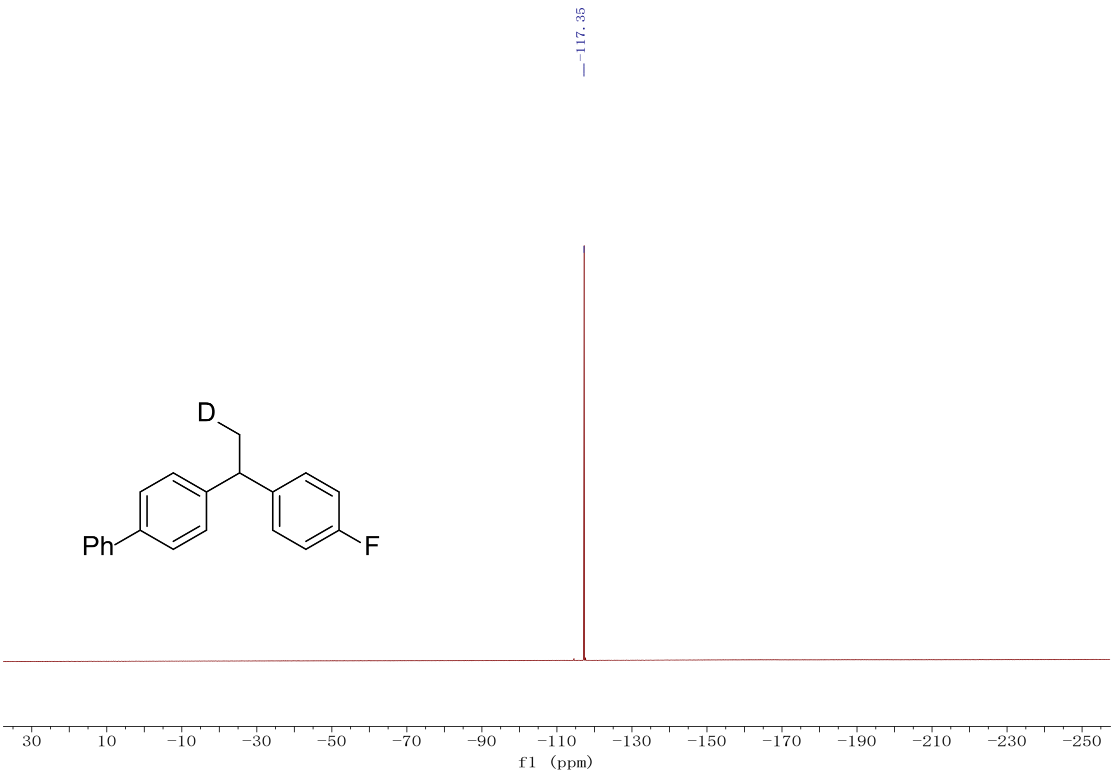

(Cyclohexyl-2-deutero)benzene (10c)

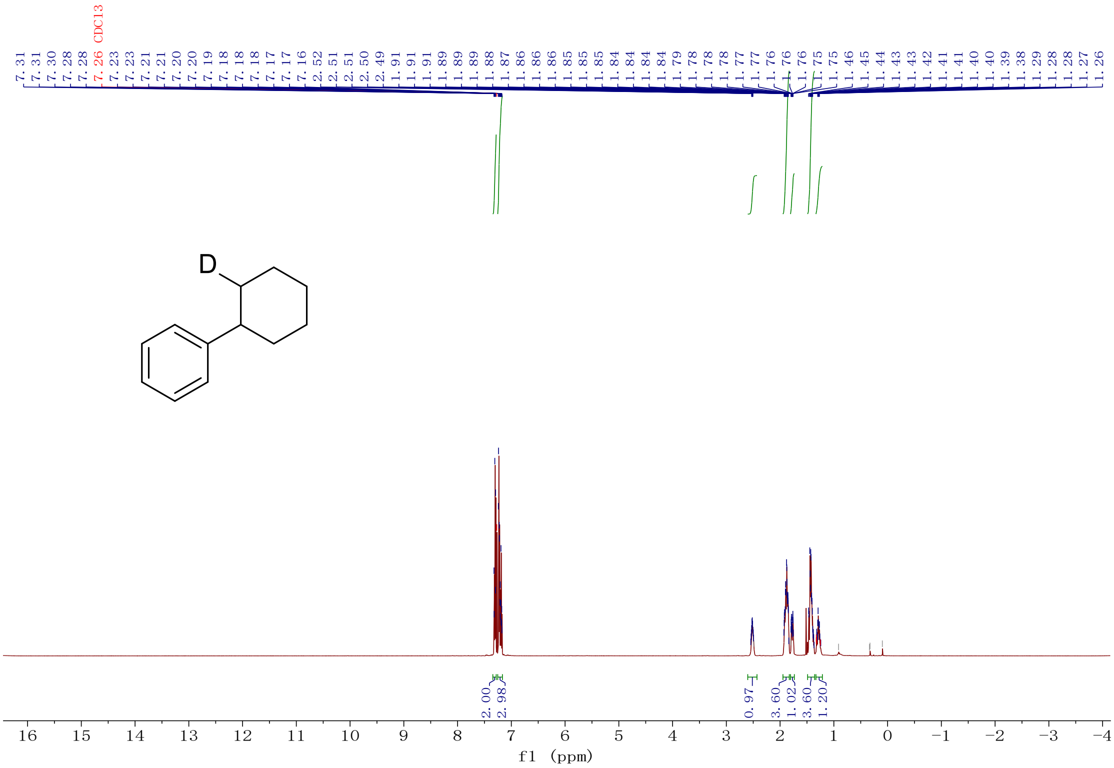

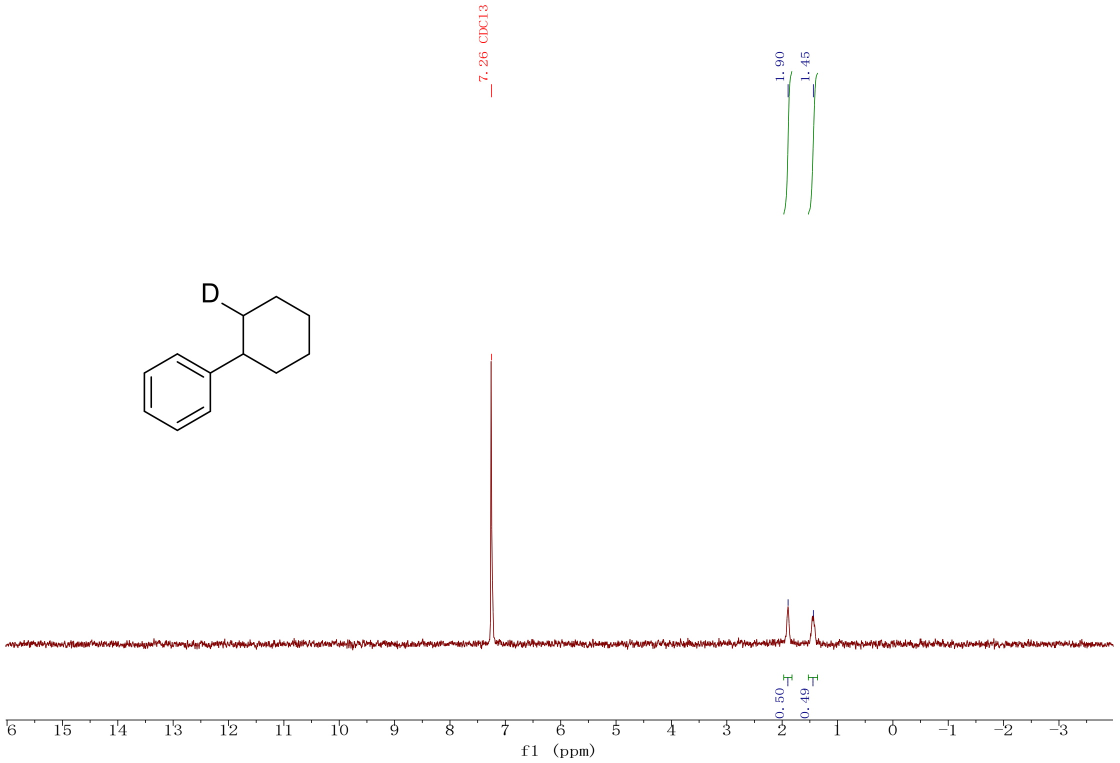
7.26 CDCl3
1.90
1.45
D
0.50
0.49
6 15 14 13 12 11 10 9 8 7 6 5 4 3 2 1 0 −1 −2 −3
f1 (ppm)

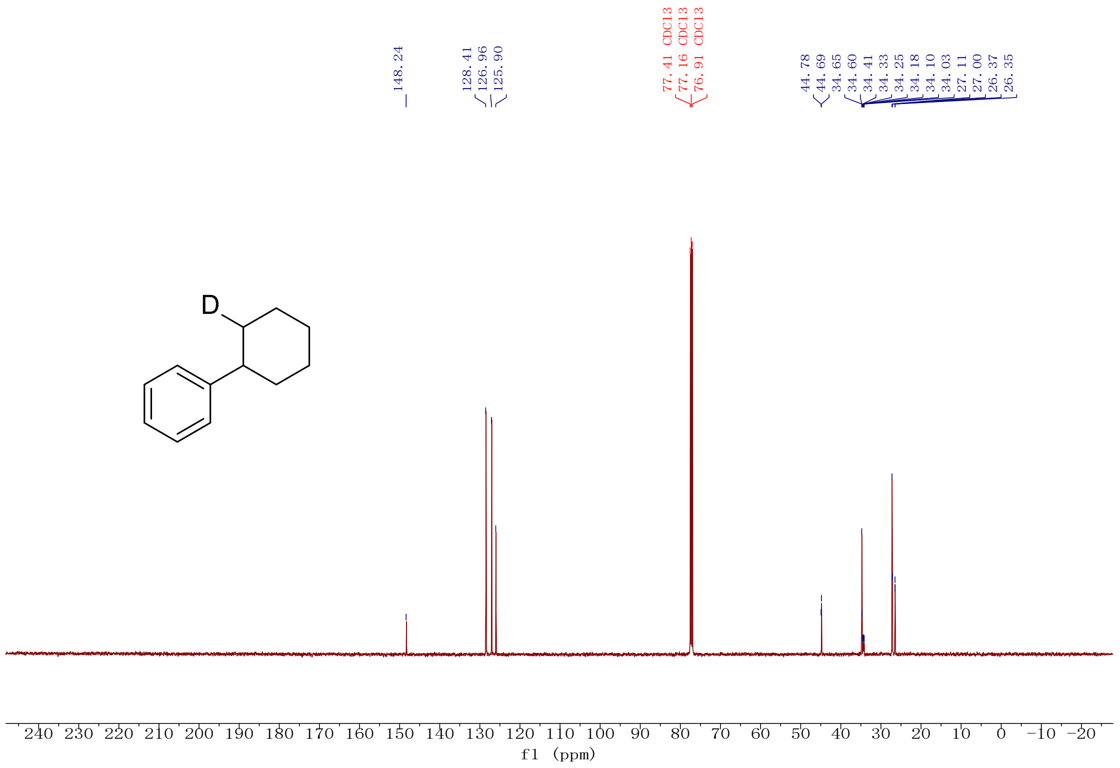
148.24
128.41
126.96
125.90
77.41 CDCl3
77.16 CDCl3
76.91 CDCl3
44.78
44.69
34.65
34.60
34.41
34.33
34.25
34.18
34.10
34.03
27.11
27.00
26.37
26.35
D
240 230 220 210 200 190 180 170 160 150 140 130 120 110 100 90 80 70 60 50 40 30 20 10 0 −10 −20
f1 (ppm)

(Propane-1,1-diyl-2-deutero)dibenzene (10d)

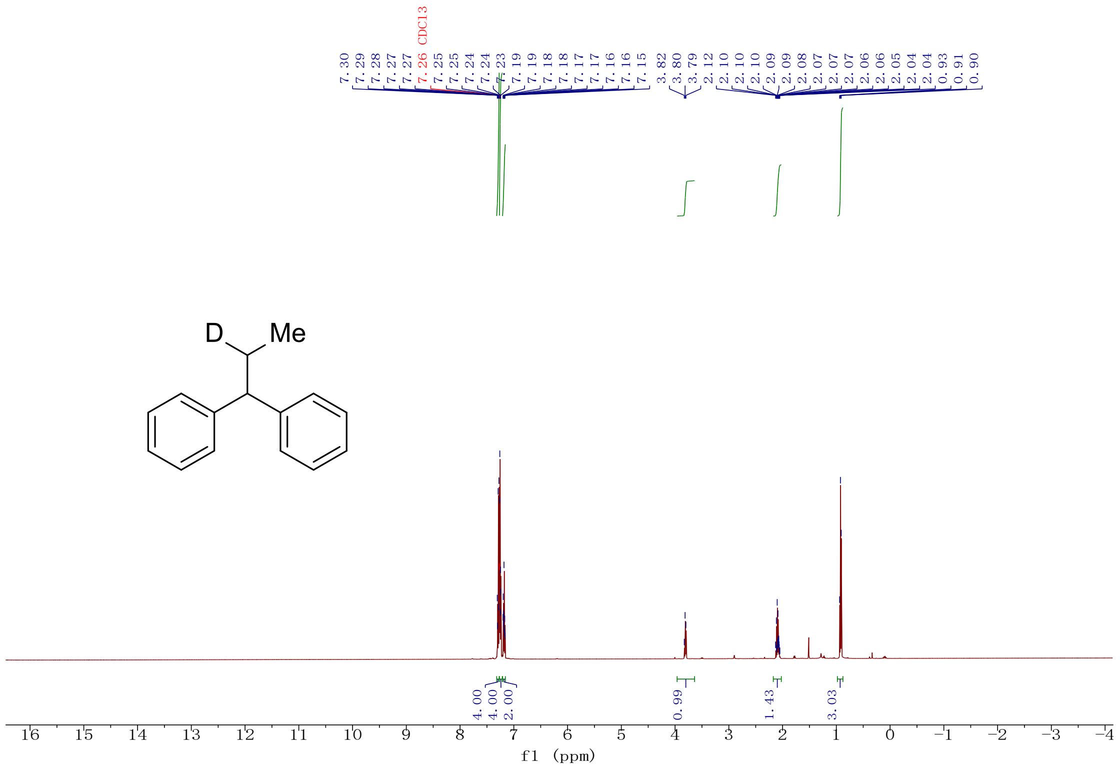

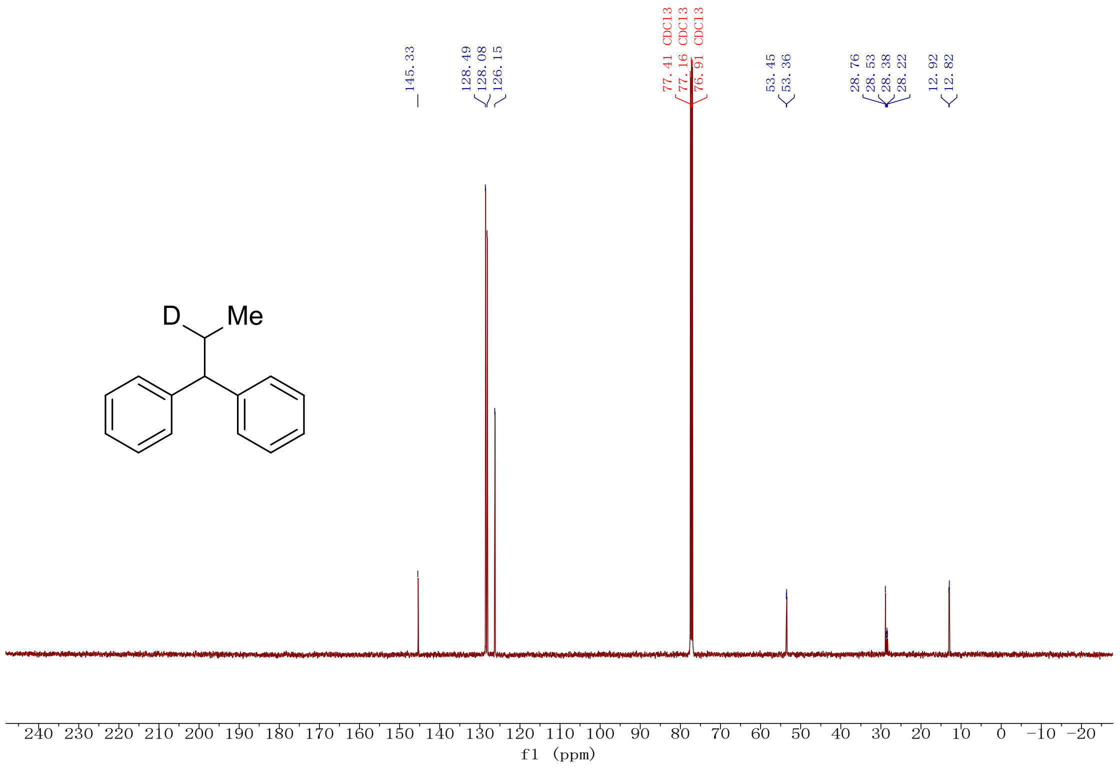

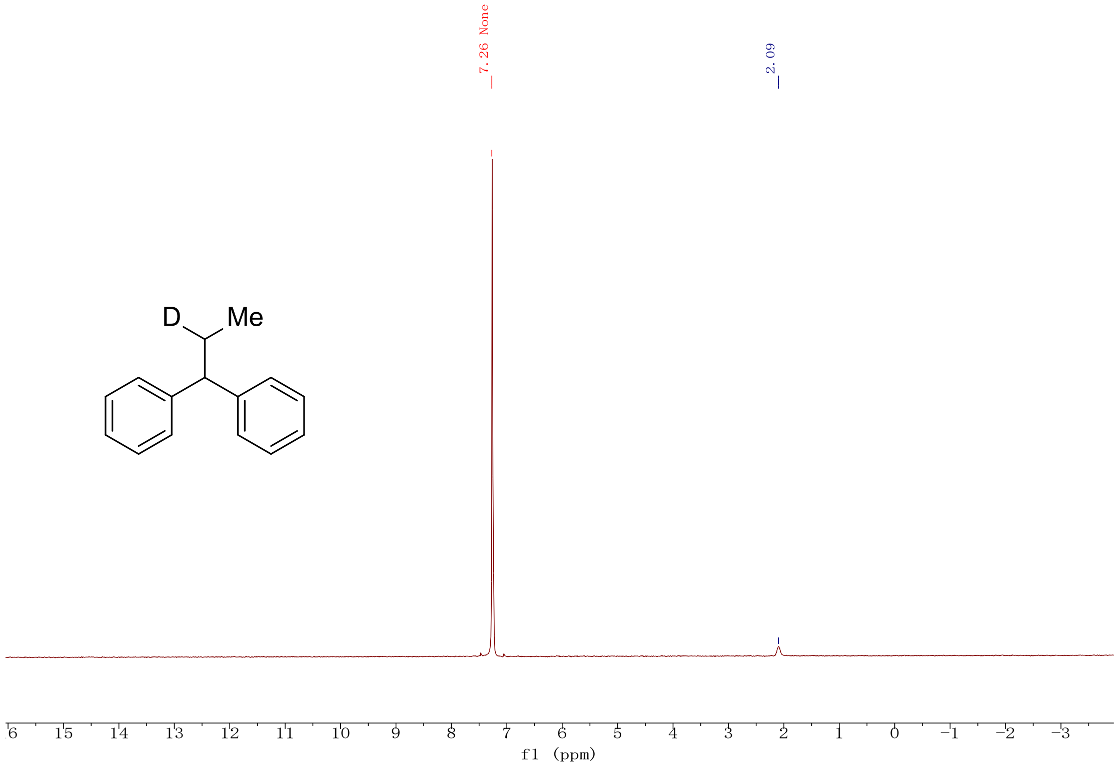
7.26 None
2.09
D
Me
6 15 14 13 12 11 10 9 8 7 6 5 4 3 2 1 0 −1 −2 −3
f1 (ppm)
